# A T2T Genome of the Lobed-Lip Iguazu Joana (*Crenicichla tuca*) Reveals Intrachromosomal Rearrangements and Trans-species Polymorphism in Cichlid Fishes

**DOI:** 10.1093/gbe/evag179

**Published:** 2026-08-11

**Authors:** Mylena Daiana Santander, Henrique Rosa Varella, Diego Baldo, Frederico Henning

**Affiliations:** Laboratorio de Genética Evolutiva “Claudio Juan Bidau”, Instituto de Biología Subtropical (CONICET-UNaM), Facultad de Ciencias Exactas Químicas y Naturales, Universidad Nacional de Misiones, Posadas, Misiones N3300LQF, Argentina; Laboratório de Biologia e Genética de Peixes, Departamento de Biologia Estrutural e Funcional, Instituto de Biociências, Universidade Estadual Paulista, IBB/UNESP, Botucatu, SP, Brazil; Laboratorio de Genética Evolutiva “Claudio Juan Bidau”, Instituto de Biología Subtropical (CONICET-UNaM), Facultad de Ciencias Exactas Químicas y Naturales, Universidad Nacional de Misiones, Posadas, Misiones N3300LQF, Argentina; Laboratório de Genética Evolutiva e Ecológica, Departamento de Genética, Instituto de Biologia, Universidade Federal do Rio de Janeiro, Rio de Janeiro, RJ, Brazil

**Keywords:** ultralong sequencing, genome architecture, speciation, replicated evolution, chromosomal evolution

## Abstract

Genomic architecture is a key factor shaping the landscape of linkage disequilibrium between divergently selected traits. By facilitating the detection of intrachromosomal variants, long-read data fueled recent interest in the role of structural variants in adaptive radiation. The Neotropical cichlid genus *Crenicichla* provides a compelling system for replicated adaptive radiation in riverine environments in which at least two lineages show repeated ecomorphological evolution. The lack of South American cichlid reference genomes has thus far limited investigation of chromosomal evolution across cichlid radiations and precluded testing the role of structural variants in *Crenicichla* adaptive radiation. Here, we generated a complete assembly of *Crenicichla tuca* (*iguassuensis* group) collected from the type locality. To investigate structural variants within *Crenicichla*, we produced ultra-long reads for *Crenicichla missioneira*. We then conduct comparative genomics across cichlids using published chromosome-level genomes. Despite karyotypic stability in *Crenicichla*, intrachromosomal structural variants are abundant and enriched in repetitive genomic regions. Comparisons with other cichlid radiations indicate the predominance of intrachromosomal rearrangements. Chromosomes LG03, LG23, LG10, and LG11 show signatures of chromosomal instability, which seem to be associated with highly repetitive DNA composition and enrichment of immune-related genes. Structural variants polymorphic in both *Crenicichla* species are consistent with the maintenance of trans-species polymorphism through balancing selection on immune-related genes. Our results add to the growing evidence that structural variation is widespread, even when overall chromosomal morphology remains unchanged. Our approach combining field-based extractions with PacBio HiFi and ONT UL proved sufficient to generate a T2T assembly and holds great potential for evolutionary and conservation genomics in biodiversity hotspots.

Significance Cichlid fishes are among the most species-rich and phenotypically diverse vertebrates. Recent evidence shows structural variants impact ecological speciation, but the comparative genomics of cichlid radiations has so far lacked South American genomes. As a result, the importance of intrachromosomal variants has been demonstrated in only a few radiations. Their prevalence in Neotropical cichlids and broader patterns of chromosomal evolution remain unassessed. We present a complete genome assembly of *Crenicichla tuca*, an emblematic species from a riverine adaptive radiation and representative of the recurrently evolved lobed-lip trait in cichlids. We detected structural variants on individual-to-intercontinental scales by comparison to ultralong reads from a key species of a neighboring adaptive radiation as well as chromosome-level genomes from Central American and African cichlids. We established the correspondence between chromosomes from different radiations, showing that intrachromosomal structural variation is abundant at multiple levels and associated with repeats. Particular chromosomes are unstable across cichlid radiations, and structural polymorphisms are shared across radiations, suggesting the persistence of trans-species polymorphism through balancing selection. The assembly includes complete centromeres and rRNA clusters and was cost-effectively obtained using long and ultralong reads with on-site UHMW-DNA purification protocols. These data highlight the power of long-read sequencing to reveal hidden variation in understudied biodiversity hotspots.

## Introduction

Genomic architecture, i.e. the physical arrangement of functional elements on the genome (Koonin 2009), plays a fundamental role during speciation and adaptation by influencing divergence under gene flow (Butlin 2005). Rearrangements affect recombination rates throughout the genome with significant impacts on speciation and adaptation. For example, inversions reduce recombination in heterozygotes by disrupting chromosome pairing and generating unbalanced gametes (Kirkpatrick 2010). Suppression of recombination promotes linkage disequilibrium within the rearrangement, enabling the joint transmission of co-adapted alleles or supergenes (Thompson and Jiggins 2014; Faria et al. 2019b; Gutiérrez-Valencia et al. 2021). Although the concept of supergenes was introduced long ago (Darlington and Mather 1949) and ever since has been advanced as key to understanding genetic polymorphism in nature (e.g. Ford 1965), genotyping structural variants (SVs) is challenging, which has limited the analyses of their role in adaptation and speciation of natural populations. Theory and recent studies suggest structural rearrangements underlie several cases of repeated adaptation and ecotype maintenance (Faria et al. 2019a; Gompert et al. 2025). For these reasons, the recent development of long- and ultra-long sequencing technologies and the ability to reveal population-level structural variation (SV) in nature has been met with enthusiasm (Ho et al. 2020; Mérot et al. 2020).

Cichlid fishes form replicated adaptive radiations at multiple timescales, making them an excellent model for investigating the genomics of adaptation and speciation (Henning and Meyer 2014). Particularly so under the “chronosequence” framework, in which different timepoints in the speciation continuum are investigated in extant species pairs (but see Stankowski and Ravinet 2021).

Despite varying in chromosome numbers from 2*n* = 32 to 60 (Poletto et al. 2010), cichlids are somewhat conserved regarding karyotypic evolution. Modal karyotypes are characteristic of the major lineages, with Neotropical cichlids typically exhibiting the cichlid ancestral state of 2*n* = 48, with variation in chromosome morphology and size. Most African Pseudocrenilabrinae have a slightly reduced complement (2*n* = 44), resulting from interchromosomal rearrangements in this lineage (Feldberg et al. 2003; Poletto et al. 2010; Paiz et al. 2024).

The advent of high-resolution genomic methods has revealed an abundance of intrachromosomal SVs in cichlids (Conte et al. 2019), and new results suggest large inversions could constitute supergenes influencing key traits (Behrens et al. 2025) and depth-related adaptation in Lake Malawi cichlids (Blumer et al. 2025). Whether SVs are a widespread mechanism in cichlid adaptive radiations remains an open question as reference genome assemblies and long-read data from South American adaptive radiations are currently lacking. This has also limited the investigation of chromosomal evolution across cichlids.

### Joanas: Emerging Models for the Genomics of Ecological Speciation

*Crenicichla*, known locally as joanas, joaninhas (Portuguese), or juanitas (Spanish), are well-suited models for replicated ecological speciation in riverine environments (Lucena and Kullander 1992; Piálek et al. 2015). The lobed-lipped joana, *Crenicichla tuca*, is one of 47 species within *Crenicichla* (Varella et al. 2023). Rapid and replicated ecomorphological evolution is pronounced within joanas (Burress et al. 2023), comprising two parallel adaptive radiations, the *missioneira* (Lucena and Kullander 1992) and the *iguassuensis* species groups (Piálek et al. 2015), from the Uruguay and Iguazu River basins, respectively.

**Fig. 1.**
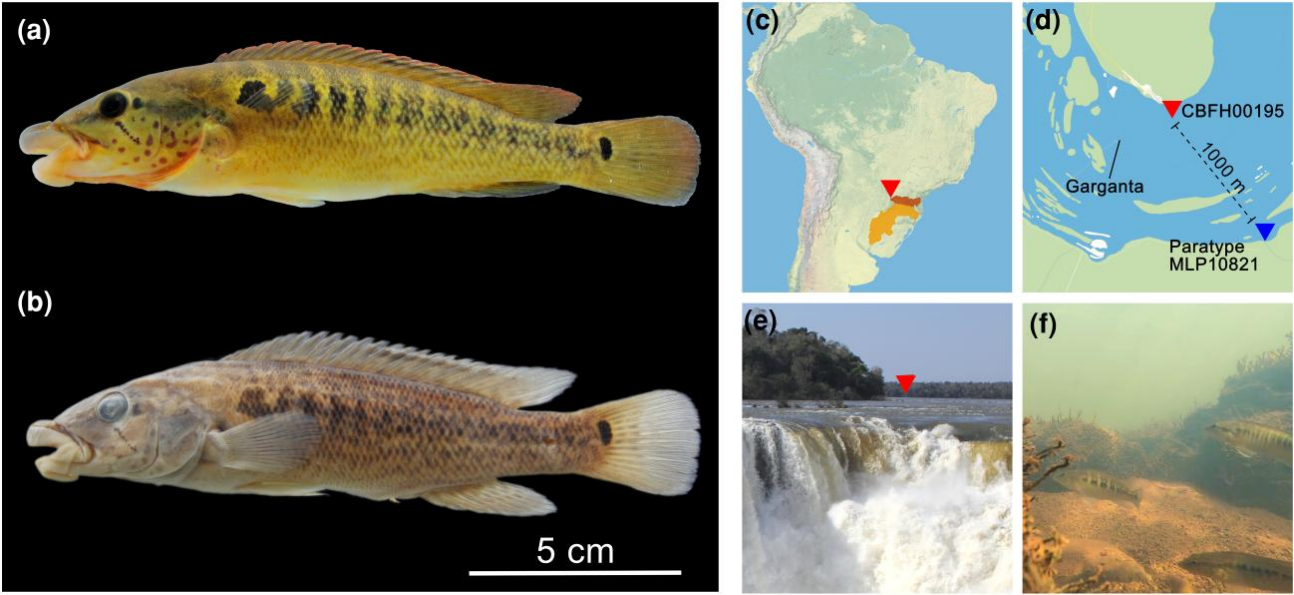
The *Crenicichla tuca* specimen used for reference genome building. Mature female photographed immediately after capture (a), specimen voucher after preservation (individual tag CBFH00195) (b). Distribution of the Iguazu and Uruguay River basins (c), inhabited by the *C. iguassuensis* and *C. missioneira* species groups (*red* and *yellow*, respectively). Map of the locality in the main channel of the Iguazu River (d), where the sequenced specimen and paratypes (MLP 10819) were collected. Photographs of the sampling site show its position relative to the escarpment at Garganta do Diabo, Iguazu Falls (e), and an underwater view f) of the site where *C. tuca* coexists with other congeners (−25.692998, −54.432864).

*C. tuca* is distinguished by its enlarged lobed lips (Fig. 1), a distinctive morphological trait and key innovation that evolved repeatedly in all known cichlid adaptive radiations (Henning and Meyer 2014; Burress 2015), including in the *missioneira* group of joanas (*Crenicichla tendybaguassu*). Lobed lips facilitate access to rocky crevices (Baumgarten et al. 2015; Henning et al. 2017) and are associated with resource partitioning among joanas adapted to shallow rapids with rocky substrates (Piálek et al. 2019; Burress et al. 2023; Varella et al. 2023).

The modal and presumably ancestral cichlid karyotype of 2*n* = 48 (Feldberg and Bertollo 1985) was recorded throughout *Crenicichla*, including lobed-lip joanas (Mizoguchi et al. 2007). No chromosomal differences were found between sexes or between the two radiations (Paiz et al. 2024). Minor variation in chromosome morphology and molecular cytogenetics suggests that chromosomal evolution in joanas is mediated by inversions (Paiz et al. 2024), but these remain elusive (Frade et al. 2019). The development of long-read sequencing will allow detecting SVs and ultimately, testing the relation between genomic architecture and the evolution of the parallel ecomorphologies in joanas.

### Toward Telomere-to-Telomere Genome Assemblies in Non-model Organisms

The production of complete, telomere-to-telomere (T2T) genomes is the final objective of genome sequencing and allows for full characterization of SVs, as illustrated by the human T2T genome (Nurk et al. 2022). Nanopore ultra-long reads (ONT UL), in which a substantial proportion of reads exceed 100 kb and run N50 values above 50 kb, resolve the most problematic genome portions, such as rDNA clusters (Rautiainen 2024). Provided that sufficient coverage of UL reads is obtained, the combination of HiFi and ONT UL alone might remove the necessity for additional scaffolding techniques, thereby reducing associated challenges toward obtaining chromosome-level scaffolding (see Yamaguchi et al. 2021). Despite this potential, obtaining suitable DNA and successful ONT UL sequencing remain technically challenging (Dahn et al. 2022). While PacBio HiFi or Nanopore long-read sequencing requires high-molecular-weight DNA with fragments above 50 kb (HMW-DNA), ONT UL requires ultra-high molecular weight DNA (UHMW), in which most fragments exceed 100 kb and can extend into the megabase range.

Here, we generated the first T2T assembly of a cichlid, the lobed-lip Iguazu joana (*C. tuca*), based on a wild-caught specimen captured in the vicinity of the paratype locality (Fig. 1), and generated ultra-long reads for *Crenicichla missioneira* from the replicated adaptive radiation of the *missioneira* group. Given the proposed role of structural variation in speciation, we tested whether the apparent karyotypic conservatism in *Crenicichla* radiations corresponds at a finer scale. We then leveraged the ultra-long reads and T2T assembly to characterize rDNA clusters bridging genomics with previous cytogenetic research while rigorously assessing assembly completeness. We extended the characterization of structural variation and established cross-species chromosomal homology across all major groups of cichlids by comparing genomes between continental radiations (i.e. African vs. Neotropical cichlids), intra-generic (i.e. *C. missioneira* and *C. iguassuensis* species complexes), and within species (i.e. between haplotypes of the *Crenicichla* species).

## Results

Sequencing of *C. tuca* using ONT UL resulted 27 Gb of sequence, of which 7 Gb were contained in reads above 100 kb (N50 = 65 kb, median quality = 20.2, longest read = 610 kb; Fig. S1). PacBio HiFi resulted in 29 Gb of HiFi reads (N50 = 13 kb, median quality = 28.8; Fig. S1). *C. missioneira* sequencing resulted in 18 Gb of ONT data (N50 = 76 kb, median quality = 21.1 and longest read = 826 kb; Fig. S1).

The lobed-lip joana assembly resulted in the 24 expected pseudochromosomal sequences, each flanked by telomeric repeats, as well as the mitochondrial genome. Together, these account for 99.9% of the assembly, which totals 839.8 Mbp and includes highly repetitive satellite sequences (e.g. centromeres) and ribosomal RNA clusters. Standard assembly statistics indicate high contiguity, base and structural correctness, k-mer and gene completeness, and absence of contamination (Table 1; Figs. S2, S3, and S4). The remaining 0.1% consists of three unassigned contigs with repetitive sequences (Table 1; Table S1). Hereafter, pseudo-chromosomal sequences are named according to the LG nomenclature based on the original Tilapia linkage maps and homology to *Amphilophus citrinellus*, assessed by whole-genome alignment.

**Table 1:** Contiguity, correctness, and general statistics of the *Crenicichla tuca* genome

*Contiguity*	
No. of sequences	27 + MT
Total scaffold length (bp)	839,745,755
Scaffold to chromosome ratio	1.13
Scaffold N50/L50	36.3/11
Scaffold N90/L90	29.8/21
Accumulated length (Mb)	839.74
Correctness	
K-mer completeness (%)	99.90
Mapping rate (%)	99.99
Split-read rate (%)	2.55
Depth	34.86
Structural error	48
Expansion	28
Collapse	17
Haplotype switch	1
Artifactual inversion	2
Small-scale assembly error/Mbp	10.92
QV	43.14
Other statistics	
GC content (%)	41.08
Repeat content (%)	37.38

While 22 pseudo-chromosomal sequences were assembled in the first assembly step of PacBio HiFi and Nanopore UL reads using hifiasm-UL, only two chromosomes (LG6 and LG14) required additional scaffolding using UL reads with ntLink and manual curation (Note S1). Duplicated haplotigs and artifactual contigs were removed during the duplicate purging step with purge_haplotigs software.

Gene annotation resulted in a high BUSCO score and OMArk completeness (Figs. S2 and S3). There was no evidence of contamination, and annotation was consistent with other fish gene sets (Fig. S3). TOGA predictions included 22,455 “intact,” “probably intact,” and “uncertain lost genes” and showed a 0.097 mono-to-multi ratio. BRAKER (33,966 genes) and GALBA (26,240 genes) results were provided here to allow comparison with previously published cichlid genomes but were considered overestimations due to several observations (Note S2).

Both telomeres are present in all 24 pseudochromosomes and are particularly evident in 22 chromosomal sequences in which there is a high terminal density of TTAGGG repeats (Table S1). LG8 and LG10 presented only a slight increase of TTAGGG repeats in one distal region. We confirmed that all chromosomes end on or close to telomeric or subtelomeric regions by exploring the distribution of TTAGGG repeats in the UL reads covering chromosome borders (Note S1). Interstitial telomeric sequences were detected in LG14 extending for 443 kb, with mean density comparable to distal regions (33 repeats/kb).

Putative centromeres were identified by detecting overlaps between large satellite arrays and decreases in sequence entropy and complexity. Most chromosome-level sequences (19) showed a single putative centromeric region. Multiple noncontiguous signals hindered the localization of five centromeres. Finally, 19 chromosomes presented both clearly identifiable centromeres and telomeres, and their morphology was approximated by estimating their centromeric index (Table S1). Most assessed chromosomes were acrocentric/telocentric (17), while the remaining were metacentric/submetacentric (2).

### Characterization of rDNA Clusters

The location of ribosomal cytogenetic markers (5S, 5.8S, 18S, and 28S gene clusters) was used to assign pseudochromosomes to chromosomes and integrate genomic data with previous cytogenetic work. In *C. tuca*, LG5 likely harbors the minor ribosomal cluster, as it showed the highest number of complete 5S rRNA copies (696). *A. citrinellus* presented 5S copies in all chromosomes and in 10 unplaced scaffolds but presented the highest copy number in LG5 (44 copies) and in the unplaced JACBYM010000415 (45 copies). Similarly, in *Oreochromis niloticus*, minor rRNA clusters were identified on LG22 (72 copies) and LG23 (82 copies) but also presented many copies in multiple chromosomes and unplaced scaffolds (e.g. 123 copies in unplaced MKQE02000026) (Table S6). The large rRNA cluster or the nucleolar organizing region (NOR) is likely located on *C. tuca* LG14, as it has more complete copies of all major subunit genes (18S, 28S, 5.8S) and shows a large tangle in the assembly graph (see below and Note S1). We could not identify the chromosome that harbored the main cluster in any of these species, as most copies were partial, and complete copies were found only on unplaced scaffolds.

**Fig. 2.**
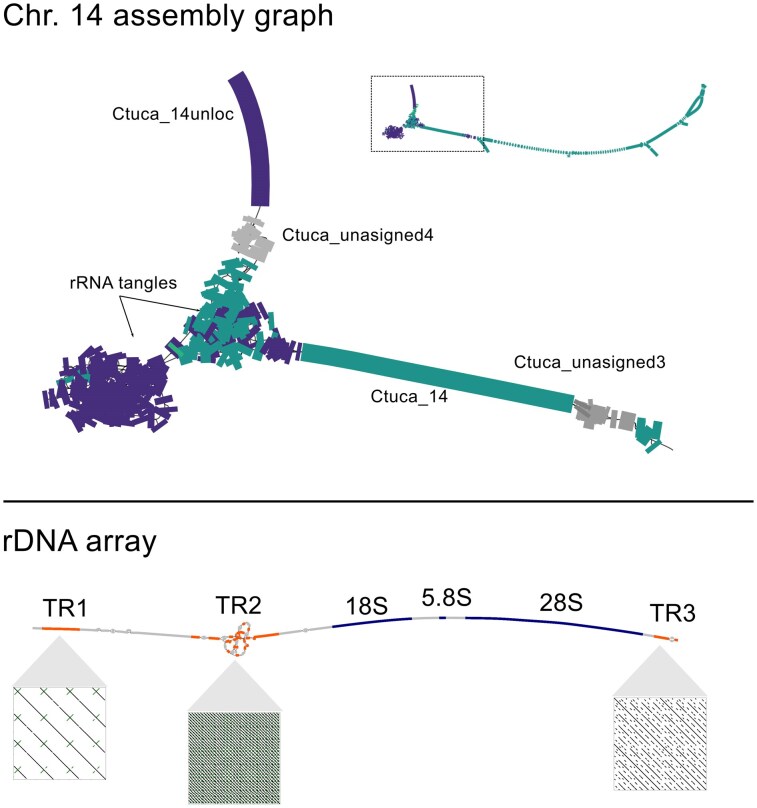
Recovery of *Crenicichla tuca* rDNA arrays and repeat units. Chromosome LG14 assembly graph highlighting rDNA tangles and contigs used during assembly manual curation (top). Bandage plot of the variant graph (bottom) built with nine rDNA morphs detected by Ribotin, with ribosomal genes in blue and within-morph tandem repeat regions (TR1–TR3) in orange. Insets (dot plots) show the internal structure of these repeats. See Note S1 for manual curation details.

Chromosome LG14 was fragmented into four parts by the presence of the rDNA array (Fig. 2): the main contig (38 Mbp) corresponding to hifiasm raw result; a ∼6 Mbp scaffold resulting from the scaffolding of two smaller contigs; and two short unassigned contigs (3 and 4). We found copies of 18S, 5.8, and 28S in the 38 Mbp contig (*n* = 7), 6 Mbp scaffold (*n* = 11), and in the unassigned 4 (*n* = 2). These observations were used to manually curate and scaffold the LG14 (Note S1) by including a refined model of the rDNA array obtained by resolving ribosomal tangles of the assembly graph.

A consensus sequence for the rDNA repeat unit was recovered, totaling 14,577 bp and harboring complete copies of 18S, 5.8, and 28S, internal- and external-transcribed spacer and intergenic regions, and tandem repeats (TRs; Fig. 2). We obtained nine rDNA morphs (i.e. variants of the repeat unit) of sizes ranging from 13,401 to 15,700 bp and genomic coverages (18 to 190×). Most variation was concentrated in the within-morph TR regions (Fig. 2). The final model for the ribosomal cluster was built by concatenating the different morphs at different copy numbers (estimated from coverage), achieving a total of 45 repeat units. The relative position of the different morphs within the array could not be determined.

### Heterozygosity and Divergence Within *Crenicichla*

SNP heterozygosity was estimated from ONT for both *Crenicichla* species, and additional estimations were performed for *C. tuca* using HiFi reads by variant calling. Based on ONT reads, *C. missioneira* heterozygous SNPs represent 0.14% of the genome, and in *C. tuca*, 0.09% (Fig. S5). Calling estimates from HiFi and ONT were concordant but lower than k-mer-based estimates (0.13% to 0.14%). Coalescent rates varied among chromosomes in both species, with a lower median rate in *C. missioneira* over the last 100 kyr (Note S3; Fig. S6). Chromosome LG3 in *C. tuca* and LG23 in *C. missioneira* showed lower coalescent rates compared to the remaining chromosomes. There were nine million homozygous SNPs between *C. missioneira* and *C. tuca*, representing 1.2% of nucleotide substitutions since the split of both lineages, a pattern shown by all chromosomes (Table S2).

### Comparative Genomics Across Cichlids

**Fig. 3.**
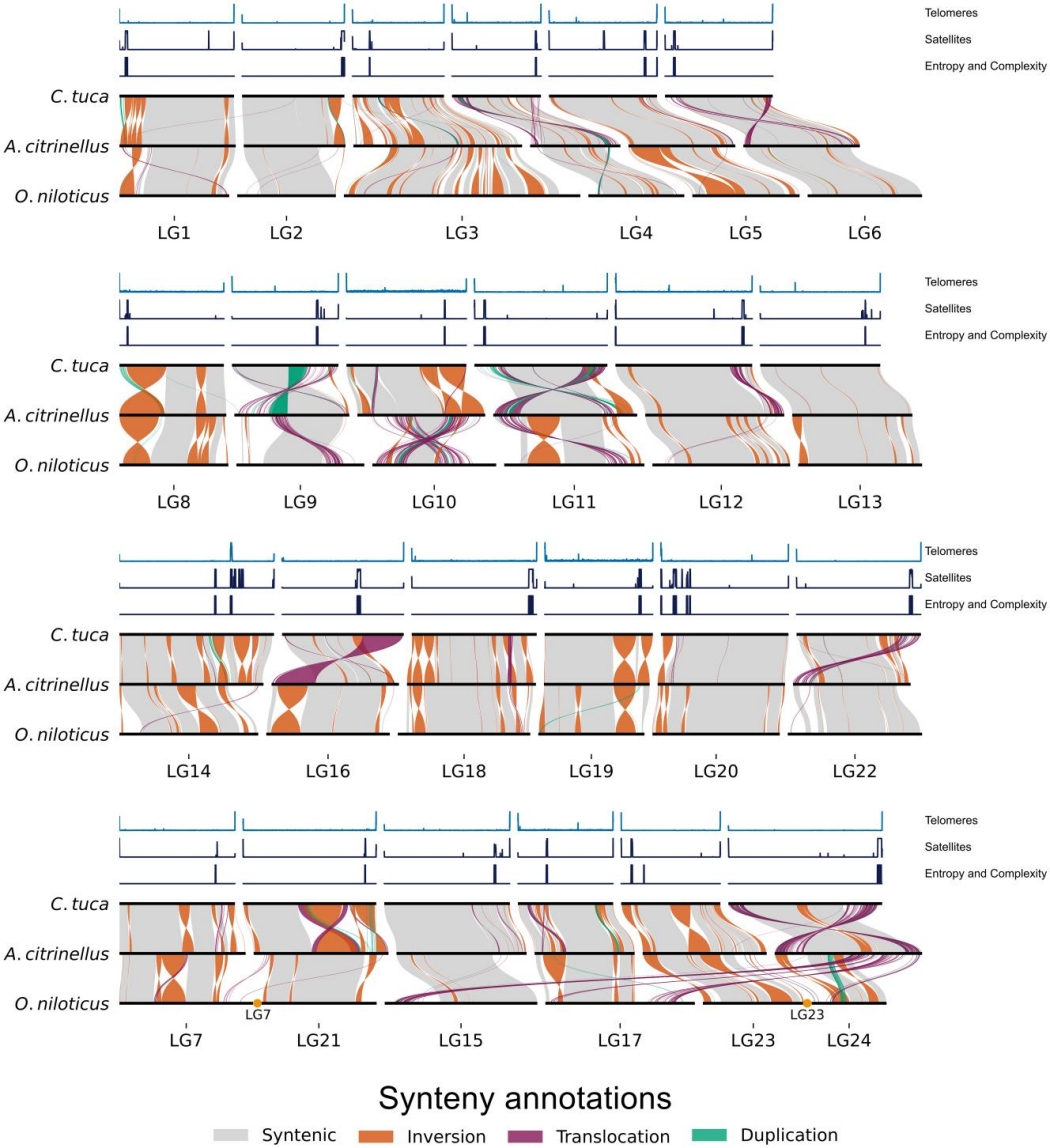
Shared synteny between the lobed-lip joana (*Crenicichla tuca*), Midas cichlid (*Amphilophus citrinellus*), and Nile tilapia (*Oreochromis niloticus*). Alignment-based structural rearrangements longer than 10 kb are shown. Tracks include information about satellite density, telomeric sequences, and reductions of entropy and complexity, calculated in bin sizes of 10 kb; *A. citrinellus* linkage groups were used as a reference for chromosome numbering. Yellow marks at the bottom indicate chromosomes involved in complex rearrangements in tilapines, and their corresponding numbering.

The comparison of chromosome-level genomes from representatives of the two Neotropical radiations (lobed-lip joana vs. Midas cichlid) revealed a high number of intrachromosomal compared to interchromosomal rearrangements. Chromosomes LG5, LG12, LG13, and LG15 were particularly conserved structurally, while in the remaining chromosomes, conserved syntenic blocks were interrupted by inversions, and less frequently by translocations and duplications (Fig. 3). Most *C. tuca* centromeres overlapped with rearrangements, and some lay within major inversions on LG8 and LG16. In total, 268 inversions were detected between both species with an average span of 18 Mb (min: 83 kb, max: 42 Mb; Table S3). Interchromosomal rearrangements mainly involved small sections (<10 kb), with no evidence of major structural differences. Most *C. tuca* chromosomes exhibit minor DNA gains or losses (<8%) relative to *A. citrinellus*, except for LG3 and LG23, which are 45% and 19% shorter than in Midas, respectively (Table S1). In *C. tuca*, these two chromosomes are reduced by 21 and 5 Mb of repetitive sequences, respectively, which likely account for the size differences.

The tilapia genome presents several interchromosomal rearrangements when compared to Neotropical genomes. These include a chromosomal fusion of the Midas cichlid LG7+LG21 (LG7 in tilapia) and complex rearrangements involving four chromosomes, giving rise to tilapia LG23 (LG23, LG24, LG17, and LG15). This chromosome could have resulted from a fusion of LG23 and LG24, and several smaller translocations would have occurred from LG24 to LG17, and LG15 (Fig. 3). Several chromosomes had decreased conserved synteny were interrupted by regions of unrecognized homology (e.g. LG2, LG9, LG22, LG15), leading to a high proportion the tilapia genome with unaligned bases (35%) to both Neotropical genomes. LG10 was too divergent between the joana and tilapia, failing to provide robust estimates of structural divergence in these species.

Several chromosomes remained structurally unchanged between African and American lineages, such as LG4, LG6, LG12, LG13, and LG20. Other chromosomes (e.g. LG5 and LG15) were conserved in Neotropical lineages but rearranged in *O. niloticus* (Fig. 3). Rearrangements specific to Midas cichlids include large inversions in LG8, LG18, and LG19, and duplications in LG4 and LG8. A large section of the Midas cichlid LG3 5′ end, as well as tilapia LG3 3′, lacks clear homology to the other genomes. Recurrent breakpoints, regions with overlap between different rearrangement types on different species, were detected on LG16, LG10, LG17, and LG24.

### Structural Variation in Joanas

**Fig. 4.**
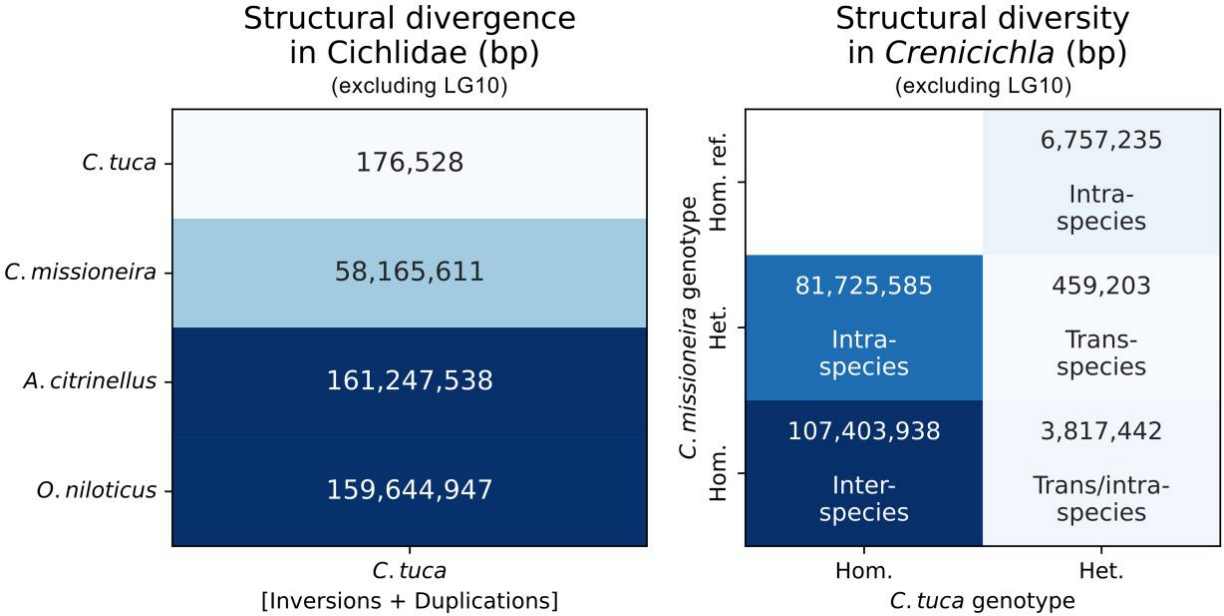
Heatmap of structural variants across major cichlid radiations. Accumulated rearrangement base pairs between different Cichlidae species (left) and within *Crenicichla* (right). For the comparison of Cichlidae, only variant categories comparable between read-based and assembly-based methods were assessed, and chromosome LG10 was skipped due to software limitations (see Methods). Hom. = homozygous; Het. = heterozygous. Hom. Ref. = homozygous reference.

By comparing a subset of equivalent categories of structural rearrangements of read-based (within *Crenicichla*) and alignment-based methods (inversions and duplications between Neotropical and African genomes), the structural divergence between *C. missioneira* and *C. tuca* is ∼1/3 (36%) of the accumulated divergence between *C. tuca* and either *A. citrinellus* or *O. niloticus* (Fig. 4). We also found segregating SVs (heterozygous) within both *C. tuca* and *C. missioneira* (Fig. 4; Table S6). These consist of candidate trans-species polymorphism analyzed in more detail below.

**Fig. 5.**
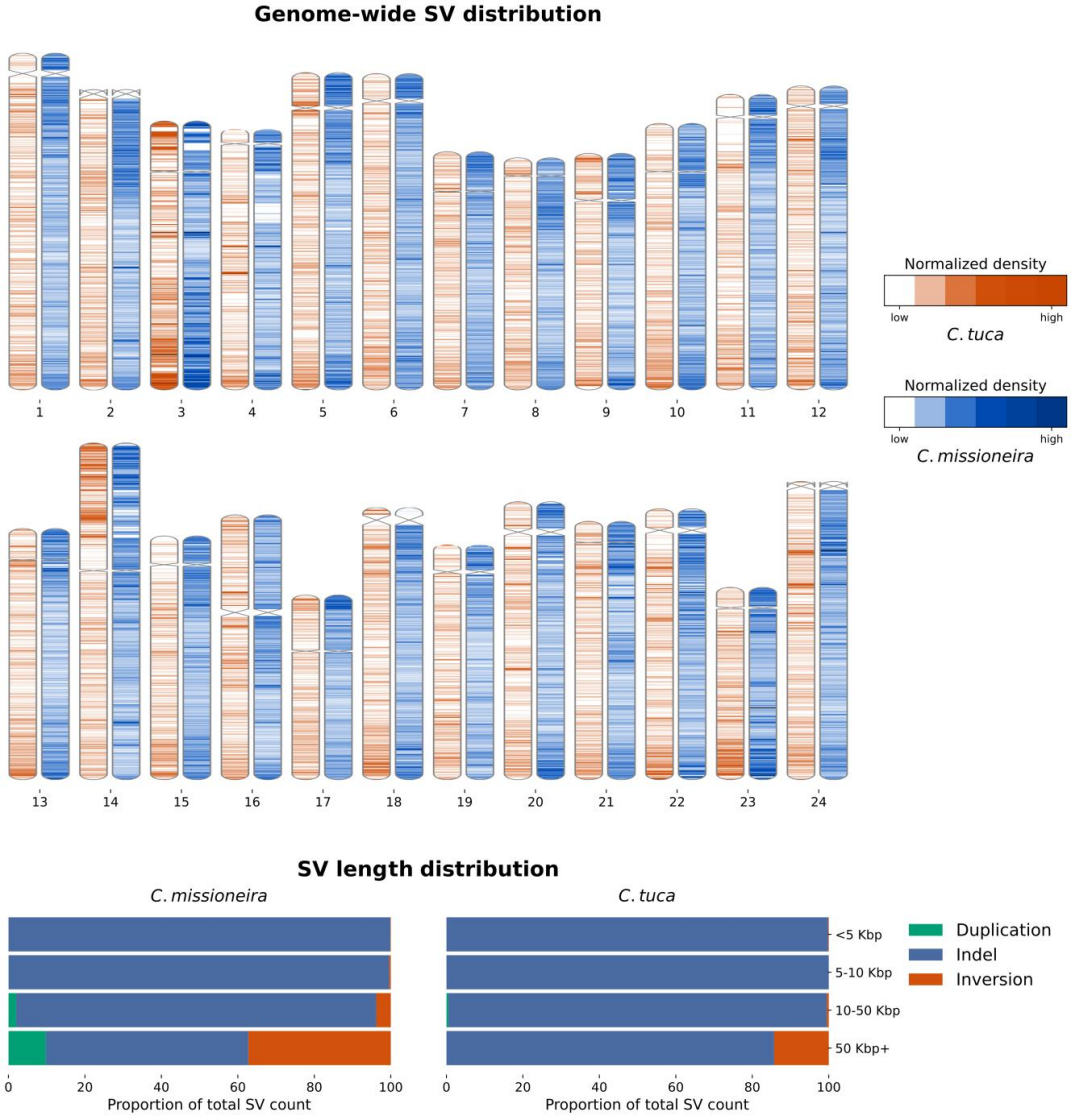
Characteristics of structural variants in *Crenicichla*. Density along chromosomes (top), distribution of rearrangement lengths (bottom). For those chromosomes with unknown centromere coordinates, the larger putative region was selected for plotting.

SVs that were homozygous for alternative alleles in both *Crenicichla* species were considered putative inter-species differences, but some might be polymorphic in populations of these species (Fig. 4). Homozygous SVs were at least 3.6 times more common than heterozygous SVs, a pattern followed by most chromosomes except LG3 and LG23 (Table S4; Fig. S7). SVs exhibited a wide range of sizes, but medium and short indels (<10 kb) represented the great majority of SVs in both species (99%). In contrast, inversions and duplications were less abundant but generally represented longer SVs (Fig. 5). Except for three small inversions (<1,600 bp), TSPs were indels, and 95% of them were shorter than 5 kb.

Heterozygous SVs were distributed across all chromosomes, with some regions presenting a higher density, especially near chromosomal ends. This distribution was similar between *C. tuca* and *C. missioneira*, although there were species- or individual-specific patterns as well (Fig. 5). The distribution of heterozygous SVs was coincident with the distribution of heterozygous SNPs in chromosomes of both species (Figs. S8 and S9). Both ends of chromosome LG3, the p-arm of chromosome LG14, and the distal q-arm of chromosome LG23 harbored an increased density of rearrangements in both species (Fig. 5).

### Functional Enrichment and Repeat Associations of SVs

**Fig. 6.**
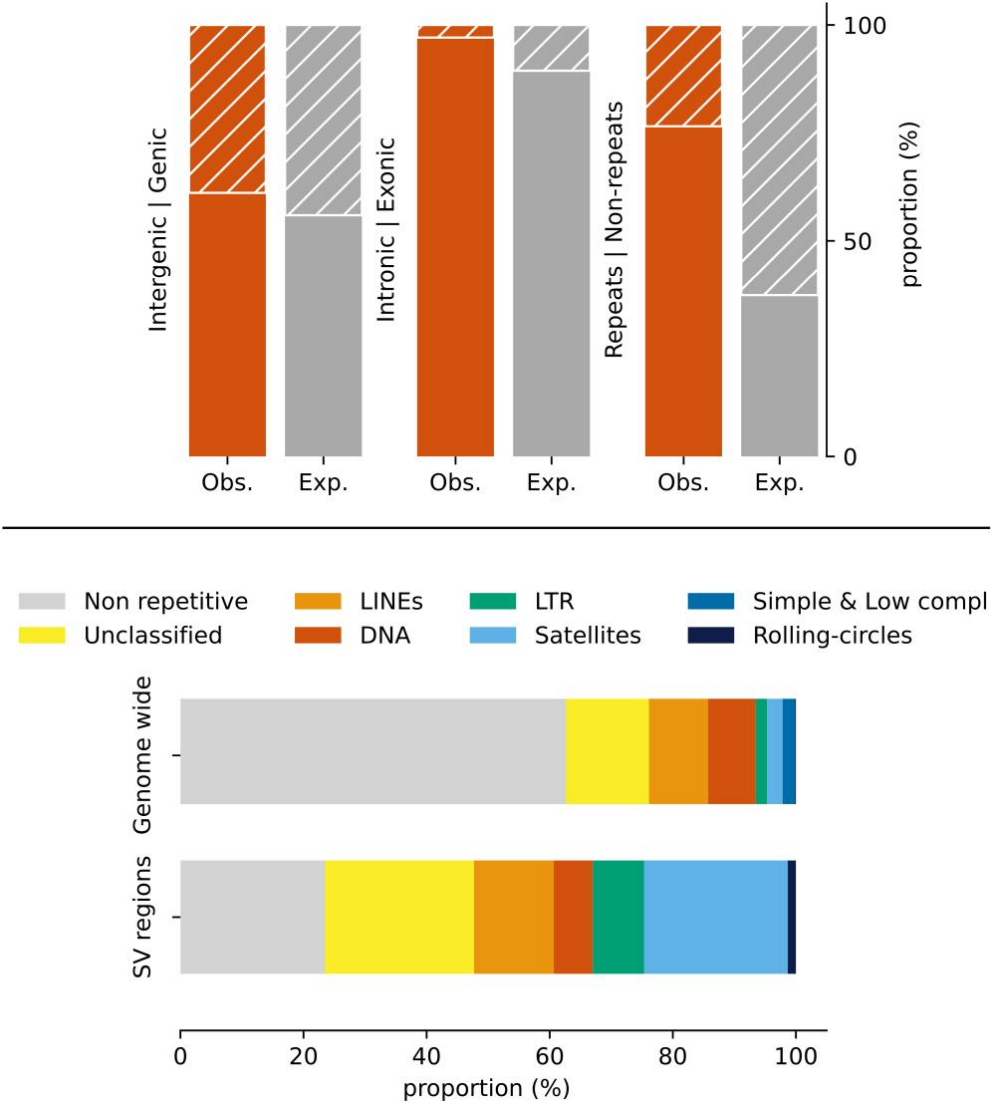
Relationship between genomic features and structural rearrangements. Genome-wide proportions of each feature type were considered as the expected proportions, while observed correspond to proportion of the same features in regions affected by SVs. Proportion of bases affected by structural variants that overlapped at least 50% with a repeat.

Structural rearrangements were slightly enriched in intergenic and intronic regions in comparison to genome-wide proportions (Fig. 6). On the contrary, they were underrepresented in genes and exons and were enriched in repetitive elements (Fig. 6). *C. tuca* SVs were, on average, 14,522 bp from genes (95% conf. interval: 13,618 to 15,486 bp), whereas TSP candidates were significantly closer (95% conf. interval: 10.056 to 12,822 bp), a pattern that remained consistent when outliers were kept. Genes closely located (<500 bp) to polymorphic SVs were enriched in GO terms associated with DNA-binding transcription factors, membrane receptor activity including immune response genes, and chromosome recombination and condensation, among others (Fig. S10). Enrichment was mostly consistent between species for indels, with few species-specific enriched GO terms, and completely diverging for inversions and duplications (Fig. S10).

Repetitive sequences represented 37% of *C. tuca*, and the majority (78%) of all rearranged bases between haplotypes (Fig. 6). Chromosomes LG14, LG22, LG3, and LG23 were the most repetitive, with proportions above 42%. LINEs were the most abundant type of classified repeat element genome-wide, with 134,537 copies, followed by DNA elements, satellites, and lastly LTRs. On the contrary, repeat proportions in structurally variant regions did not show the same distribution (Satellites > LINEs > LTR > DNA), reflecting a differential impact of repeat types on SVs and genotyping biases. In chromosomes LG23 and LG3, there was a high coincidence of chromosomal windows with high SV density and high density of different repeats (Fig. S11). Distal regions of the two arms of chromosome LG3 showed the lowest SV density, which were in fact artifacts due to stringent variant filtering in regions of high repetitiveness. Moreover, there was a decrease in the homozygous/heterozygous ratio in LG14 and LG23 of *C. missioneira* (Fig. 4; Fig. S7).

### Trans-species Polymorphism in Joanas

**Fig. 7.**
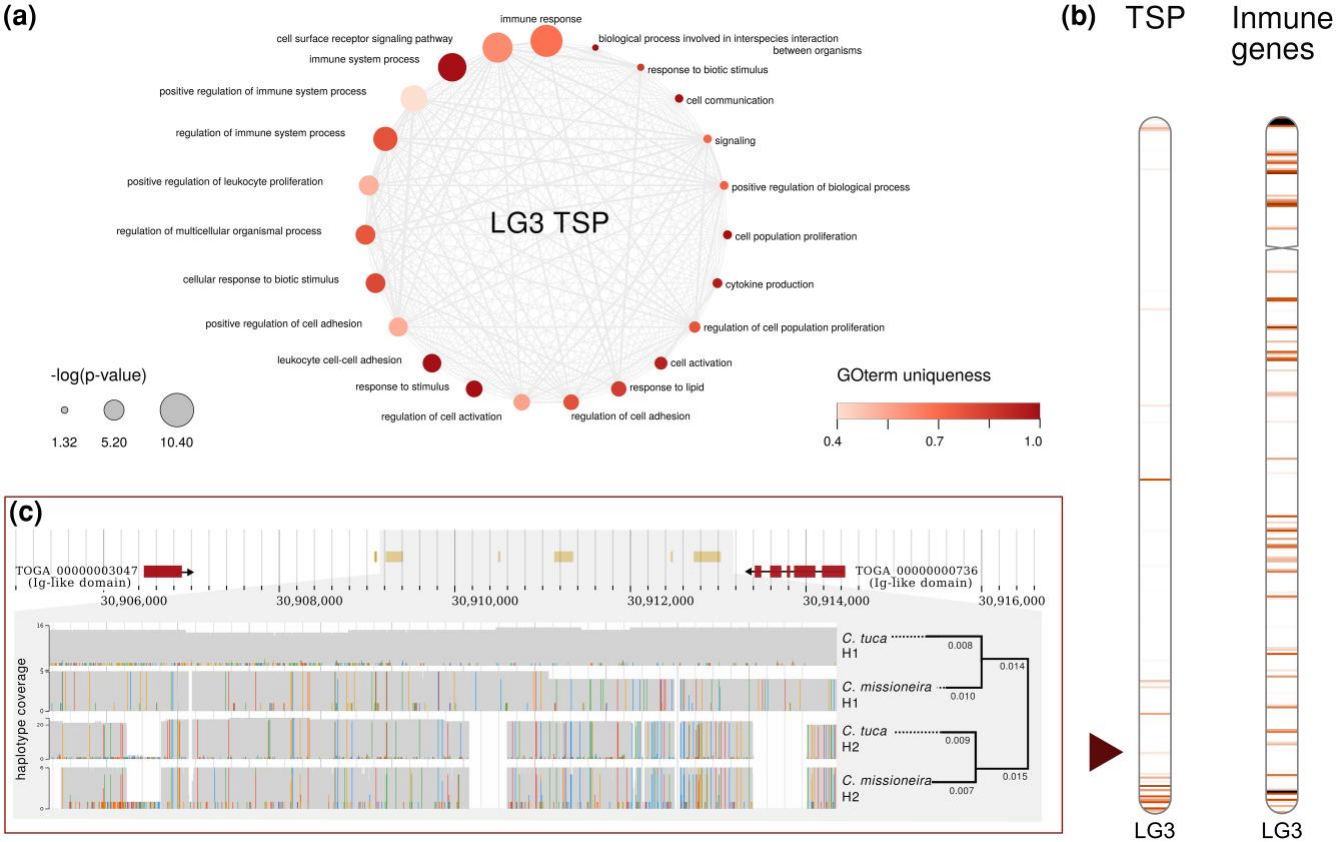
Trans-species polymorphism in an LG3 structural haplotype. a) Enrichment of genes located near TSP. Synteny between haplotypes within and between species (top). b). Distribution of SV TSPs and immune-related genes on LG3. c) Detailed view of a candidate region for trans-species polymorphism and distance trees for the shared haplotypes. Detailed view of the coverage differences indicating polymorphic indels located near two immune-response-related genes, homologous to tilapia's ENSONIG00000008284 and ENSONIG00000035933.

Chromosome LG3 showed twice the number of shared SVs as any other chromosome (132 TSPs; 77 precise, 55 with varying coordinates; Table S5). TSP indels close to (<500 bp) genes were enriched in highly connected GO terms associated with immune response (Fig. 7a, Fig. S10). TSPs are distributed across LG3 (Fig. 7b). We tested the hypothesis of trans-species polymorphism on a candidate region differing by three indels between two immune genes (Fig. 7c). The two structural haplotypes cluster phylogenetically and show an older divergence than genome- and LG3-wide estimates between species using both distance (Jukes–Cantor distance between haplotypes was 0.05 vs. 0.012 genome-wide) and maximum likelihood methods (Note S4). The divergence within structural haplotypes is more recent, but still older than the genome average (Jukes–Cantor distance 0.018 for H1 and 0.016 for H2).

## Discussion

By combining accurate long- and ultralong reads, we assembled the complete genome of a wild-caught *C. Tuca*, thus enabling high-resolution genomics of the remarkable riverine adaptive radiations of joanas in South America. The comparison with other cichlid genomes revealed ample intrachromosomal variation, which, despite karyotypic conservation in *Crenicichla* constitutes a plentiful substrate for altering the genomic landscape of recombination. Comparative genomics further revealed SVs at all levels, indicating chromosomal instability across lineages, as well as intra-, inter-, and trans-species polymorphism within lineages.

### Efficient T2T Assemblies for Non-model Organisms

We obtained a cichlid T2T assembly by combining ONT UL and PacBio HiFi reads without additional scaffolding. Although the technical difficulties associated with obtaining sufficient UHMW-DNA have motivated recent efforts to achieve T2T assemblies without ONT UL, ultra-long reads still exceed other approaches in reconstructing full chromosomes (Cheng et al. 2026) and modeling highly abundant rDNA morphs similar to the human CHM13 T2T assembly (Rautiainen 2024). Here, we show that obtaining UHMW-DNA is not only non-prohibitive but also efficient when fresh samples of species of reasonably sized genomes are available. In our case, the use of fresh blood for on-site UHMW-DNA purification (protocol is provided in the Data Availability section) resulted in suitable input for ONT UL, yielding 9× genome coverage in ultralong reads (>100 kb as per Nurk et al. 2022).

The lobed-lip joana genome presented here is fully contiguous and captures complex repetitive regions, including rDNA clusters (notably 28S), which are typically unresolved in chromosome-level genomes (Table S7). Our genome assembly can be considered complete, or “telomere-to-telomere” (T2T), since: (i) 99.91% of the total sequence is assembled in chromosomes with a 99.99% read mapping rate; (ii) all chromosome sequences are bound by telomeric repeats; (iii) even the most difficult regions comprising putative centromeres and ribosomal DNA could be assembled; and (iv) the different gene predictions are concordant with described gene sets for fishes (Note S2). The remaining unassigned scaffolds represent less than 0.01% of the genome and are composed of repeats and repetitive genes such as immunoglobulins and might represent misassembled contigs or unremoved haplotigs. The final genome assembly showed negligible differences (6% to 7% of assembly size) to the k-mer based genome size estimations, which is concordant with the assembly size range and quality of other cichlids from African (Conte et al. 2019) and Neotropical lineages (Kautt et al. 2020). Future efforts should be directed toward expanding potentially collapsed repeats, completing the whole telomeres of LG10 and LG8, correcting small misassemblies and polishing, and further improving rRNA sequence models by the determination of the correct order and count of the different rRNA morphs.

### SVs Across Different Timescales

We characterized numerous SVs that accumulated since the split between South American, Central American, and African lineages, despite the karyotype stability in chromosome number (Feldberg and Bertollo 1985; Amores et al. 2014). The SVs we detected sum to the growing evidence that several large rearrangements, such as inversions, translocations, and, to a lesser extent, fusions, have accumulated in different cichlid lineages (Conte et al. 2019; Quah et al. 2025).

Consistent with the divergence times between Central and South American lineages (68 to 90 Mya; Schedel et al. 2019) and between African and Neotropical lineages (78 to 106 Mya; Irisarri et al. 2018), collinearity analysis shows that joanas and Midas cichlids share higher synteny than either does with tilapia (Fig. 3). While some chromosomes (e.g. LG13 and LG20) remained largely unchanged, others were highly rearranged (e.g. LG03, LG10 and LG23). In highly divergent chromosomes such as LG10, detection of SVs reached the resolution limit of whole-genome alignment, precluding precise quantification of SVs. Consequently, the amount of SVs between Neotropical and African lineages is underestimated, as LG10, one of the most divergent chromosomes, was excluded (Fig. 4). This is evident in the collinearity patterns, in which LG10 shows moderate rearrangement between Neotropical lineages, but extensive reorganization and numerous breaks in collinearity relative to tilapia.

Neotropical cichlids present a high amount of intrachromosomal rearrangements. On the contrary, no large interchromosomal rearrangements were detected, which explains the conserved chromosome numbers between joanas and Midas cichlids. Inversions were the most abundant type of rearrangement between Neotropical species (in terms of accumulated bp), with most chromosomes presenting at least one inversion. Many of these inversions were exclusive to *A. citrinellus* and absent in *C. tuca* and *O. niloticus*. While these may reflect a higher rate of inversions in Central American cichlids, the potential for assembly artifacts must be considered and independently validated.

Large interchromosomal rearrangements have occurred in the African tilapine lineage, particularly involving their chromosomes LG7 and LG23. Here, we show that the tilapia LG7 fusions involved mainly the chromosomes corresponding to LG7 and LG21 in Neotropical cichlids, and tilapia chromosome LG23 involved the Neotropical chromosomes LG23 and LG24 in addition to several small rearrangements that likely involved translocations from Neotropical LG24 to tilapia's LG15 and LG17. Conte et al. (2019) previously identified these rearrangements by comparing tilapia with a non-cichlid fish (*Oryzias latipes*, medaka). Considering their homology assignments, the Neotropical LG23 and LG24 would correspond to medaka chromosomes 2 and 4, respectively. Our results confirm previous findings and determine that these fusions are exclusive to the tilapia lineage and are absent in Neotropical cichlids. Other interchromosomal changes have been recently found among African cichlids, including fusions giving rise to sex determining chromosome systems (Conte et al. 2019; Behrens et al. 2025), but remain undetected at the cytogenetic level.

The genus *Crenicichla* presented both fixed and segregating SVS. These have been differentially distributed in genic vs. intergenic, exonic vs. intronic, and repetitive vs. nonrepetitive regions in *Crenicichla* genomes. Although biases may be present due to limitations in aligners and variant callers when resolving complex repetitive regions, these results can also reflect selective constraints on coding regions: i.e. most recent structural variation is likely to represent insertions and deletions of satellites, TEs and other repeats, rather than structural variation affecting single-copy coding regions, coinciding with recent findings in Lake Malawi cichlids (Quah et al. 2025). Although less abundant than insertions and deletions, inversions and duplications were also found within *the genus*, and due to their larger size than other SVs, they can potentially affect multiple genes. When closely located to genes, INDELS were enriched in similar functional categories related to expression regulation in both *Crenicichla*, while inversions and duplications affected divergent functional pathways in each species. However, the functional impacts of these SVs remain untested in both African and South American cichlids.

Most structural variation within *Crenicichla* was concentrated at the ends of metacentric chromosomes and the distal ends of the long arms in acro-telocentric chromosomes, coinciding with SNP heterozygosity. In tilapia, chromosomal ends tend to present lower recombination than other chromosomal regions (Conte et al. 2019), a pattern contrary to what was observed in most fishes (Shanfelter et al. 2019). Structural variation presents a dual relationship with recombination. Although different SVs are known to create and occur in regions of low recombination where selection is less effective, they can also be enriched in regions of high recombination due to more frequent errors during meiosis. The patterns of SV and SNP distribution would indicate that chromosomal ends have higher recombination in *Crenicichla*. Other additional information is required to clarify whether the SV distribution in *Crenicichla* correlates with high-recombination regions or if these patterns are driven by alternative evolutionary forces such as selection.

### Structural Instability and Trans-species Polymorphism in LG3

Chromosome LG3 is unusually large, repetitive-rich, and highly rearranged in African cichlids (Conte et al. 2019, 2021). In *C. tuca*, LG3 underwent substantial reduction in size in comparison to *A. citrinellus* by the loss of repetitive DNA. Other relevant characteristics of *Crenicichla* LG3 were: (i) high repetitive content; (ii) high structural and nucleotide variation both between *C. tuca* haplotypes and between *Crenicichla* species; (iii) significant trans-species structural polymorphism; and (iv) lower coalescent rate in *C. tuca*. A similar pattern of high SNP and structural variation, high repetitive content, and lower rate of coalescence was observed in LG23, although the latter was only observed in *C. missioneira*.

Although a fraction of the 132 putative instances of TSP (*n* = 55) on LG3 may represent homoplasy, most are compatible with the notions of ancient TSPs maintained through balancing. Including: (i) coincidence of breakpoints, (ii) deep coalescence, and (iii) enrichment in immune genes. MHC loci and other immune-related loci frequently present alleles that remained polymorphic through several speciation events under balancing selection (Fortier and Pritchard 2025), and due to their old coalescence, these chromosomes can represent hyperdivergent regions (reviewed in Klein et al. 1998; Moya et al. 2025). The high structural divergence of LG3 could also represent hyperdivergent regions in *Crenicichla*, deriving from the presence of immune-related gene clusters. Given that the divergence between haplotype groups exceeded the genome-wide divergence and assuming comparable mutation rates, some of these shared SVs might have been segregating since the split of both radiations 8 Mya (Burress et al. 2023). Long-term maintenance of these haplotypes could be facilitated by the large SVs present in LG3 with potential effects on recombination. Nonetheless, the persistence of such an old polymorphism is improbable under neutrality alone, and balancing selection should be considered for future studies.

### Cytogenomic Integration

Despite the great interest in cichlids, cytogenetic studies are scarce and mainly focused on the South American Heroini tribe (Poletto et al. 2010; Valente et al. 2012; Mendoza-Alfaro et al. 2025). It remains unclear whether the intrachromosomal rearrangements revealed here are observable at the cytogenetic level and whether they have a relationship with the Heroini adaptive radiation. Because many inversions involve the putative centromeres, these structural differences could be traced by cytogenetics. The structural variation observed in *A. citrinellus* can be further tested as new chromosome-level genome assemblies for Heroini become available and encourage cytogenetic investigation within the tribe.

*C. tuca* chromosomes were numbered taking into account *A. citrinellus* genome to allow comparisons between African and Neotropical genomes, a naming scheme that is based on the original linkage maps in tilapia. Despite facilitating comparisons between distant cichlid radiations, this genome-based chromosome nomenclature might hamper integration with cytological observations. Several previous works have highlighted the need to establish nomenclature conventions that integrate genomics and cytogenetics (Lewin et al. 2019; Yamaguchi et al. 2021; Chen et al. 2023). For example, LG3 is the largest metacentric chromosome (1 M) in tilapia's karyotype, while it is not homologous to *Crenicichla* chromosome 1 M, but to a medium-sized acrocentric. Due to the high conservatism in chromosome number and structure, determining the correspondence between cytogenetic karyotypes and genomes would be highly feasible (e.g. Simakov et al. 2022). Since the chromosomes are represented by single pseudomolecules in the *C. tuca* genome, two chromosomes were assigned to the *Crenicichla* karyotypes described by Paiz et al. (2024).

The assembly of the ribosomal clusters and their localization in the assembly graph allowed us to correlate pseudomolecules to additional molecular cytogenetic markers. Based on our findings, *C. tuca* chromosome LG14 corresponds to the largest and NOR-bearing chromosome of the karyotype (1 M; metacentric) and LG5 to chromosome 4ST (subtelocentric). Additional evidence of the correspondence between chromosome LG14 and chromosome 1 M is the presence of interstitial telomeric sequences, which are thought to reflect an origin via chromosomal fusion and were reported previously using cytogenetic methods (Frade et al. 2019). Our estimated centromeric indexes (Table S1) and repetitive markers for most chromosomes (Fig. S12) offer a roadmap for assigning genomic assemblies to physical chromosomes in other Neotropical cichlids, particularly for the similarly sized medium chromosomes.

### Leveraging Evolutionary and Conservation Genomics in Biodiversity Hotspots

While obtaining complete assemblies is increasingly feasible, their autonomous production in the Global South is still limited. Our approach for obtaining a T2T assembly using on-site UHMW-DNA extraction (see Data Availability) is particularly well suited to settings in which access to fresh specimens is not limiting. It satisfies several desired properties for building genomic capacity in the Global South (Lawniczak et al. 2022), being cost-, computationally-, and logistically efficient by relying on only two sequencing technologies and circumventing challenges associated with sample preservation and shipment. In addition, it adds value to genomic research in biodiversity-rich regions by potentially increasing the biological significance of specimens used for reference genome building. While we built a reference from a specimen from the type location to better represent the population originally used in species description, studies could extend this principle to use holotypes as targets for reference genome building, creating the first “hologenotypes.”

The Iguazu joanas are under significant threat due to operating and planned hydroelectric power plants in the Rio Iguazu basin. Lobed-lipped joanas inhabit areas already impacted by hydroelectric development or threatened by proposed small-scale facilities (e.g. the Rio Guarani). These are thought to already have reduced the habitable area to one-fourth of its original size (Piálek et al. 2019). While the common Iguazu joana (*C. iguassuensis*) is widespread in the Lower Iguazu basin, Lobed-lip joanas are strictly associated with shallow rapids and are considerably less abundant (Lucena and Kullander 1992; Piálek et al. 2015; Varella et al. 2023). Our results support these concerns as individual heterozygosity is lower in *C. tuca* compared to *C. missioneira*, which could result from the smaller historical population sizes associated with the dependency on rheophilic habitats (Note S3). We highlight the need for targeted field surveys to inform conservation efforts in the Iguazu joanas, which can now be performed at high resolution thanks to the availability of this complete reference genome (e.g. characterization of homozygosity runs, environmental DNA, scans for local adaptation loci).

## Conclusions

This study highlights the presence of SVs at all levels in cichlids. At higher phylogenetic scales, large structural rearrangements accumulated between Neotropical and African species, some of which resulted in changes to both chromosome number and morphology. Between South American lineages, there were no fusions or fissions between chromosomes, but inversions were prevalent. Despite their roughly identical karyotypes, joanas exhibit diverging genomic architectures with SVs that were both fixed between species and segregating within the genus, forming trans-species polymorphism. The present study establishes a foundation for future population-level research to assess the adaptive impact of these variants and aid conservation of the joana adaptive radiations. Our approach enables efficient T2T assembly, facilitating genome production in the Global South while increasing the biological significance of reference genomes.

## Materials and Methods

### Sampling, Morphological Identification, and DNA Extraction

The reference assembly was obtained by combining ultra-long Oxford Nanopore (ONT) and PacBio sequencing technologies for a single wild-caught female individual of *C. tuca* (individual tag CBFH 000195). This specimen was captured using a hook and line baited with worms at the Iguazu River main channel, just above the escarpment at “Garganta do Diabo” (“Devil's Throat”), Iguazu Falls, inside the “Parque Nacional do Iguaçu (PNI)” in Brazil, coordinates −25.692998, −54.432864. This locality, in the opposite margin of the locality of paratypes MLP10819, corresponds to the lowermost limit of the distribution of the species in the Iguazu basin. After being captured, the specimen was photographed alive, euthanized using tricaine, and, after having blood and tissues collected, was fixed in formaldehyde solution (4%) and preserved in ethanol solution (70%). Besides the presence of lobed-lips, this specimen is readily identified as *C. tuca* by color pattern features, viz. presence of only one to three unocellated blotches on the anterior portion of the flanks, below upper lateral line, instead of several dark blotches along the flanks; presence of a yellow-orange ground coloration with large orange dots on the cheek and opercular series; dark suborbital stripe reduced and decomposed into a few widely separated dots. Three other species of *Crenicichla* were captured on the same site: *Crenicichla tapii*, *C. iguassuensis*, and *C. tesay*. The specimen of *C. tuca* was identified as female by the sexual dimorphic feature of having a dark band along the dorsal fin as well as ripe mature oocytes.

Ultra-high molecular weight DNA (UHMW-DNA) extraction was performed for Nanopore UL sequencing using Circulomics Nanobind Kit with UHMW DNA for nucleated blood according to the manufacturer but with slight modifications to adapt it for a field setting (Field-based UHMW-DNA extraction protocol, see data availability). Briefly, blood was extracted by heart puncture after anesthesia (MS-222) using a 1 mL syringe and a 21 G needle flushed with 0.5 M ethylenediaminetetraacetic acid (approximately 5 μl), and transferred to a blood collection microtube containing ethylenediaminetetraacetic acid to avoid coagulation prior to DNA extraction. A sample of 20 μl of blood was processed in less than 24 h for DNA extraction. For PacBio sequencing, high molecular weight DNA (HMW-DNA) was extracted using an adapted phenol:chloroform protocol (Sambrook and Russell 2006; Kim et al. 2021) from ethanol-preserved liver tissue of the same individual. An additional wash step was performed to rehydrate the liver tissue following (Kim et al. 2021), and tissue homogenization was done using a glass Dounce homogenizer on ice, followed by lysis with 20 μl proteinase K (20 mg/μl) and 4 μl of RNase A (100 mg/μl) in a vertical rotor (9 rpm). In both HMW- and UHMW-DNA extraction, mechanical shearing of DNA molecules during pipetting was avoided by gentle handling and use of wide-bore tips.

*Crenicichla missioneira* was sequenced with UL ONT using the same methods stated for *C. tuca*, for a single wild-caught male individual (CBFH00251, specimen deposited in the collection of the Laboratorio de Genetica Evolutiva—CONICET-UNaM under accession LGEP1165) from Arroyo Melo of the Uruguay River basin (−27.417778, −54.7019444).

### Sequencing and Genome Assembly

PacBio library preparation and HiFi sequencing were performed by Maryland Genomics (US) on a Revio system. Oxford Nanopore library preparation was performed using the Ultra-Long DNA Sequencing Kit V14 (SQK-ULK114) with wash-reload steps to increase output, and sequencing was performed on an R10.4.1 PromethION flow cell (FLO-PRO114M) by Nanopore Canada (Montreal, Canada). For a general assembly workflow, see Fig. S13.

HiFi reads were used for genome parameter estimation with *k* = 21 and *k* = 31 (genome size, heterozygosity, repetitiveness) with Genomescope2 (Ranallo-Benavidez et al. 2020; Fig. S14). The quality of the sequencing reads was assessed using Nanoplot before and after trimming using cutadapt and porechop_abi (Martin 2011; Bonenfant et al. 2023; De Coster and Rademakers 2023).

Genome assembly was obtained using hifiasm, combining both read technologies using the Ultra Long integration option (Cheng et al. 2024). The primary assembly was used for downstream analyses. Duplicated haplotigs and artifactual contigs including unassigned repeats were assessed and removed using purge_haplotigs (Roach et al. 2018). Resulting contigs were aligned to the *A. citrinellus* genome (GCA_013435755.1), which shares an overall conserved karyotype 2n = 48, and visualized with D-genies (Cabanettes and Klopp 2018) and numbered according to their linkage groups (LGs) for consistency. Chromosomes that were apparently fragmented into 2 or more contigs were highlighted (LG6 and LG14). We first scaffolded these with ntLink (Coombe et al. 2023) using ultra-long ONT reads (>100 kb) and then visualized the read coverage of the junction. Manual steps involving visualization of the assembly were required to completely scaffold the LG14, which was found split into different contigs by the localization of the rRNA (Note S1). Contigs belonging to LG14 were joined together to a model of the rRNA cluster (see below) separated by 1000 Ns.

The mitochondrial genome was assembled using MitoHiFi (Uliano-Silva et al. 2023). The mitochondrial hifiasm contig (originally named “unassigned_1”) was removed and replaced with MitoHiFi assembly, since both showed high similarity (>99%), but the former was a concatemer (Fig. S15).

Complementary approaches were used to assess assembly quality, continuity, and completeness of the raw, purged, and scaffolded assemblies: BUSCO analysis; Kat k-mer completeness (Mapleson et al. 2017); Inspector assessment for base and structural quality (Chen et al. 2021); Blobtools and NCBI FCS workflow for contamination (Challis et al. 2020; Astashyn et al. 2024).

### 45S rRNA Cluster Assembly and Characterization

To obtain a consensus sequence of the 45S rRNA cluster, Ribotin (Rautiainen 2024) was run in hifiasm mode using HiFi and UL ONT libraries, with manually selected tangles (Figs. S16 and S17) from the assembly graph and a morph size of 8,000 bp. The obtained consensus was annotated for rRNAs with barrnap (Seemann and Booth 2018) and trimmed/reorganized to match the standard rRNA 5′ to 3′ order (18S–5.8S–28S). Ribotin was run once again with the modified consensus as a reference to build a new consensus and retrieve different morphs oriented 5′ to 3′. Repeats were searched in the consensus visualizing self-dotplots using FlexiDot (Seibt et al. 2018). All obtained morphs were used to build a pangenome graph with PGGB (Garrison et al. 2024). Visualization of the built variant graph and all other assembly graphs, together with the localization of rRNA genes and repeats associated with the cluster, was done with Bandage (BandageNG fork; Wick et al. 2015). Copy number of each morph was estimated as the morph coverage divided by haplotype sequencing depth following (Rautiainen 2024). To build a model of the rRNA cluster, the different morphs were repeated as many times as estimated copies and joined together in arbitrary order.

### Coalescent and Heterozygosity Estimates

To estimate genome-wide heterozygosity, HiFi and ONT reads of *C. tuca* and ONT reads of *C. missioneira* were aligned to the reference genome with minimap2, and alignment files were quality filtered (mapQ >30). SNP variant calling was performed using DeepVariant, and VCF files were filtered with bcftools to keep high-quality heterozygous SNPs (variant score >20, genotype = 0/1 or 1/2 and custom coverage cutoffs: Note S3). The number of heterozygous SNPs was calculated per 100 kb windows and visualized with RIdeogram (Hao et al. 2020). Changes in the coalescent rate were assessed per chromosome using msmc2 with default parameters (Schiffels and Wang 2020), using the filtered variants file, a mappability mask generated with mosdepth (maxCov > depth > minCov; Pedersen and Quinlan 2018), and using the satellite bed file as a negative mask. Ne was estimated by transforming the scaled coalescent rate assuming panmixia, a 3-year generation time (pers. observations and aquarists’ estimates) and a 3.5e−9 mutation rate (Malinsky et al. 2018). Chromosome- and genome-wide distances between species were measured with Jukes–Cantor, considering the homozygous differences between species and the mappable genomic bases.

### Structural Rearrangements in Cichlid Genomes

Chromosome scaffolds of *C. tuca*, *A. citrinellus* (GCA_013435755.1), and *O. niloticus* (GCA_001858045.3) were compared by aligning with minimap2 (asm20 presets), followed by detecting SVs with SyRI (Goel et al. 2019). Given the ancient divergence between American and African lineages, and SyRI requirements of an equal number of pseudochromosomes for comparison, *O. niloticus* chromosomes resulting from interchromosomal rearrangements were manually split, based on whole-genome alignments from D-genies and homology estimations from Fixchr. Based on the same evidence, *O. niloticus* and *A. citrinellus* chromosomes were reversed-complemented when required for comparing the same strand. Resulting rearranged and syntenic regions, together with different feature tracks, were plotted with plotsr (Goel and Schneeberger 2022).

Given the low divergence between *Crenicichla* genomes and the draft nature of the *C. missioneira* genome assembly (Note S6), SVs between *C. tuca* and *C. missioneira* were called using a read-based approach. Nanopore reads of both samples were aligned using minimap2, variants were called with Sniffles2 (Smolka et al. 2024), and merged using Jasmine (Kirsche et al. 2023). SVs were quality filtered with custom scripts by removing those SVs with low support (<mean coverage/4), no breakpoint coverage, or too high breakpoint coverage due to mapping of repeat-derived reads as follows. SRs were removed if coverage at one end exceeded twice the coverage of the opposite breakpoint or represented twice the mean read coverage. Homozygous reference calls were removed from both *C. tuca* and *C. missioneira* vcf files, while homozygous variant calls (1/1) in *C. tuca* were considered as assembly or miscalling errors. Shared SVs were estimated based on the supporting vector information of Jasmine output. Polymorphic and overlapping SVs in both species were considered precise if their start and end coordinates were concordant, differing by less than 10 bp. A Generalized Linear Mixed Model (GLMM) with a negative binomial distribution was employed to test differences in the distance to the nearest gene between TSPs INDELs and INDELs exclusive to *C. tuca*. To account for chromosome-specific gene densities, chromosomes were included as random effects. The differences were tested for the whole dataset as well as by removing outliers with the IQR method.

The density of rearrangements was estimated in 100 kb windows using bedtools coverage (Quinlan 2014) and visualized with RIdeogram. Estimation of the intersection between genes, repeats, and intergenic features with SRs was performed with custom scripts using pybedtools (Dale et al. 2011), for which only intrachromosomal SRs were considered. An SV was considered to map to a repeat if at least 50% of its bases overlapped with any repeat. We calculated the proportion between the observed number of bases that intersected between SVs and the different features and the total number of SV bases, and compared with the background genomic proportion in *C. tuca* (e.g. repeat bases/total genomic bases). To compare both assembly-based and read-based estimates, we summarized relevant information from the software outputs, restricting the analysis to comparable variant types and skipping LG10, which presented a major and divergent inversion in the *C. tuca* × *O. niloticus* comparison, preventing a successful SyRI run due to homology detection limitations.

### Gene and Repeat Annotation

Satellite DNA was annotated using SRF (Zhang et al. 2023) and soft-masked from the assembly with RepeatMasker (Smit 2004; Fig. S19). Then, other types of repeats were annotated using RepeatModeler2 (Flynn et al. 2020) and LTR finder (Xu and Wang 2007) integrated in EarlGrey (Baril et al. 2024), using Dfam database (Storer et al. 2021). Manual curation of repeat *consensi* was performed by manually visualizing structural and protein features following Goubert et al. (Goubert et al. 2022; Note S5). The inclusion of repetitive gene families in the repeat library was avoided by searching all repeat consensus against Pfam (Mistry et al. 2021) and manually filtering the sequences containing homology to known multigene families and no TE-related features. When possible, *consensi* classified as “unknown” by EarlGrey were classified into TE types based on hits with TE-related proteins and structural features (e.g. terminal inverted repeats for DNA/TIRs). When chimeric *consensi* were detected because of over-extension with EarlGrey, these were replaced with their ReapeatModeler2 shorter counterparts. The final repeat library was used to soft-mask the genome with RepeatMasker.

Gene annotation of the soft-masked genome was initially tested with three different tools Note S2; Fig. S18). Finally, TOGA (Kirilenko et al. 2023) was chosen as the main annotation method and run to infer gene annotations and assign orthology to *O. niloticus* genes. This species was chosen as a reference for the annotation as it has been annotated by different methods with multiple sources of evidence, and there is isoform data available. As TOGA results include probably lost or inactive genes, gene predictions were filtered to keep only intact, probably intact, and uncertain lost genes, while lost and missing genes were removed. The completeness and other statistics of the resulting gene models and annotations were assessed using BUSCO (Actinopterygii db_10) (Manni et al. 2021), OMark (Nevers et al. 2024), and Agat (Dainat 2024). Ribosomal genes (28S, 18S, 5S, and 5.8 rRNA) were predicted using Barrnap and eukaryotic Hidden Markov models (Seemann and Booth 2018). Genes were functionally annotated by finding the functional annotations of tilapia homologous genes and enriched with g:Profiler, using Tilapia genomic background and filtering for the top five highlighted terms of each category. Alternatively, proteins were annotated with eggnog mapper.

All descendant terms of “immune system process” (GO:0002376) were retrieved with the ontologyIndex R package, and these terms were searched in eggnog annotations to obtain coordinates of immune-response-related genes.

### *Crenicichla* LG3 TSP Haplotypes

SNP phasing and read happlotaging were performed with WhatsHap (Martin et al. 2022) and ONT reads. Haplotype 1 (H1) and Haplotype 2 (H2) sequences were obtained by splitting the BAM file by PS tag and obtaining a single consensus sequence per haplotype. Resulting haplotypes were aligned with Muscle, Jukes–Cantor distances and distance-based neighbor-joining tree were obtained with MEGA, excluding indels.

### Centromeres, Telomeres, and Chromosome Assignments

Centromeres are composed of tandemly repeated satellites that result in a decreasing linguistic sequence complexity and sequence entropy. These last two genomic statistics were calculated in sliding windows of size 10 kb using Nessie (Berselli et al. 2018). Contiguous windows (covering over 100 kb) that showed a decrease in both complexity and entropy were marked as putative centromeres following Brekke et al. (2023), which colocalized with satellites (Fig. S19). On the other hand, the presence of the standard vertebrate telomeric sequence (TTAGGG) in chromosomes was assessed using TIDK search in windows of 1 kb (Brown et al. 2025). Telomeres were considered present if any of the five distal windows presented a density 10 times higher than the chromosomal baseline, in reverse or forward orientations. A read-based analysis was performed to confirm that all chromosomes were assembled to their ends, using UL reads mapping to the last 30 kb of both the 5′ and 3′ termini. Reads were reverse complemented when necessary, and the density of telomeric repeats was estimated in 10 kb windows from read ends using TIDK (Note S1).

We evaluated the correspondence between genomic sequences and cytogenetic chromosomes based on the localization of previously reported cytogenetic markers such as the large ribosomal subunit/NORs, the small ribosomal subunit 5S, interstitial telomeric probes, and chromosome morphologies. We ordered chromosomes harboring putative centromeres and telomeres in two groups, collapsing the morphological categories of Levan et al. used in *Crenicichla* cytogenetics (Levan et al. 1964; Paiz et al. 2024): (i) those with centromeric index (CI) 25 to 50% corresponding to metacentric and submetacentric chromosomes; and (ii) those with a CI 0 to 25% corresponding to subtelocentric and acrocentric/telocentric chromosomes. CI was estimated by taking the middle coordinate of the putative centromere region and the chromosome size for those chromosomes deemed “complete” (with both centromeric and telomeric inferred regions). The karyotypes considered for comparison are *C. iguassuensis* and *C.* sp. “big lips” from the Iguazu River (Mizoguchi et al. 2007; Paiz et al. 2024).

## Permits

*C. tuca* was collected under permit 75043 (SisBio, Brasil). *C. missioneira* was collected under permits 09/2023 (Ministerio de Ecología, Argentina) and 9950 to 16-2023 (Instituto Misionero de Biodiversidad, Argentina).

## Supplementary Material

evag179_Supplementary_Data

## Data Availability

All genomic data including assembly and raw reads were deposited in GenBank (Bioproject PRJNA1266708). All code used for assembly, annotation, and plotting is found in https://github.com/mylena-s/assembly_project/tree/CtucaBranch. The field protocol for UHMW-DNA extraction from fresh nucleated blood is available at the protocol section of the laboratory website (https://www.henninglab.com/protocols). Additional files such as gene predictions, repeat libraries, and several annotation tracks were deposited in Figshare 10.6084/m9.figshare.29070893.
