# Supplementary material for: A T2T Genome of the Lobed-Lip Iguazu Joana (Crenicichla tuca) Reveals Intrachromosomal Rearrangements and Trans-species Polymorphism in Cichlid Fishes
Source: Genome Biol Evol. 2026 Aug 11;18(8):evag179. doi: 10.1093/gbe/evag179 (PMC13458148; doi:10.1093/gbe/evag179)
Supplement: Supplementary file 1 [file evag179_supplementary_data.pdf]

Supplementary Material

Table of Contents

Supplementary figures......1
Supplementary figure S1. 2
Supplementary figure S2. 2
Supplementary figure S3. 3
Supplementary figure S4. 4
Supplementary figure S5. 5
Supplementary figure S6. 6
Supplementary figure S7. 7
Supplementary figure S8. 8
Supplementary figure S9. 9
Supplementary figure S10. 10
Supplementary figure S11. 11
Supplementary figure S12. 12
Supplementary figure S13. 13
Supplementary figure S14. 13
Supplementary figure S15. 14
Supplementary figure S16. 15
Supplementary figure S17. 16
Supplementary figure S18. 16
Supplementary figure S19. 17
Supplementary tables......17
Supplementary table S1. 17
Supplementary table S2. 20
Supplementary table S3. 21
Supplementary table S4. 22
Supplementary table S5. 23
Supplementary table S6. 24
Supplementary table S7. 26
Supplementary notes......27
Supplementary note S1. 27
Supplementary note S2. 30
Supplementary note S3. 31
Supplementary note S4. 33
Supplementary note S5. 35
Supplementary note S6. 39

Supplementary figures

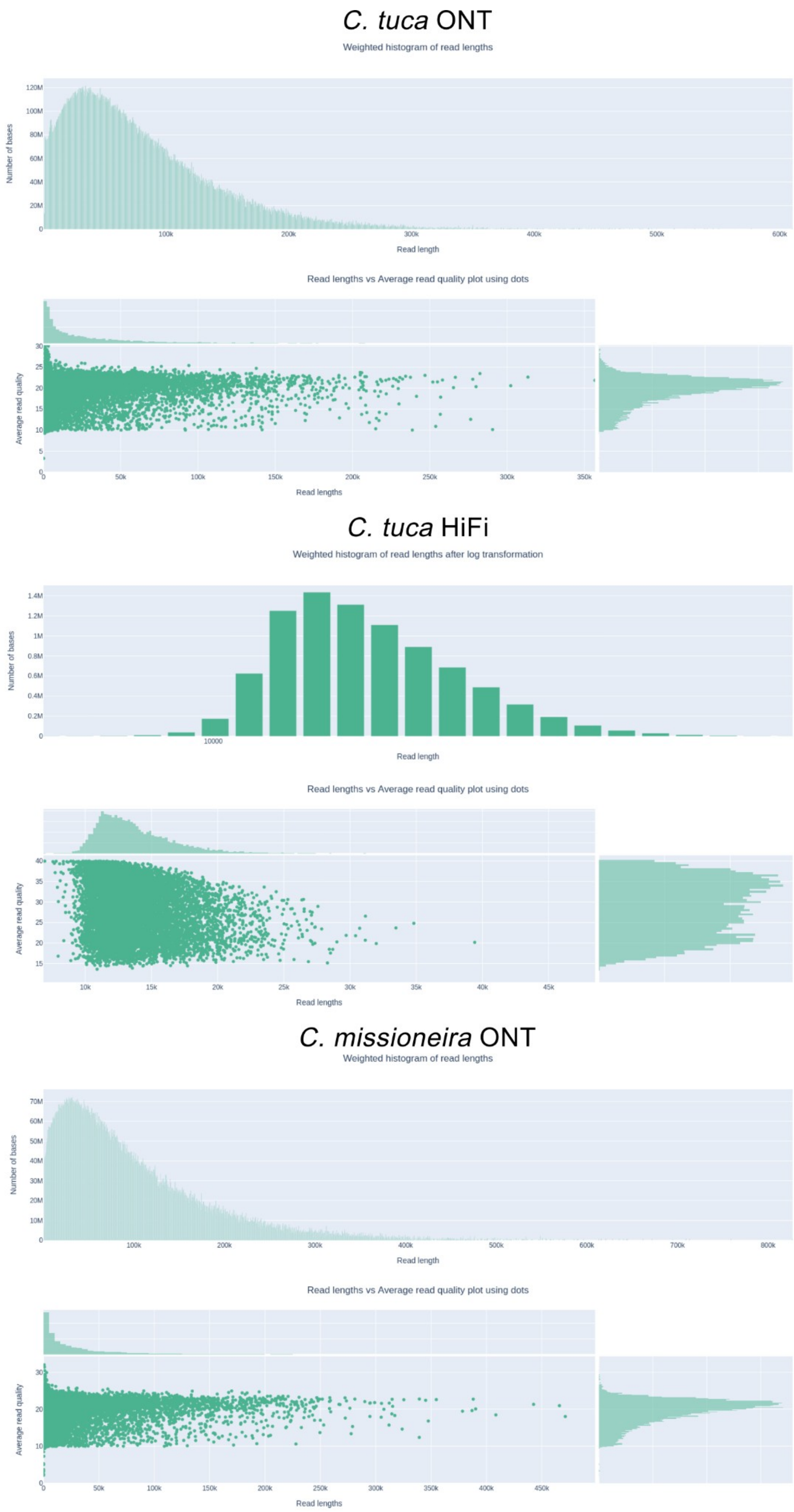

Supplementary figure S1. Read quality of sequencing results.

Supplementary Material

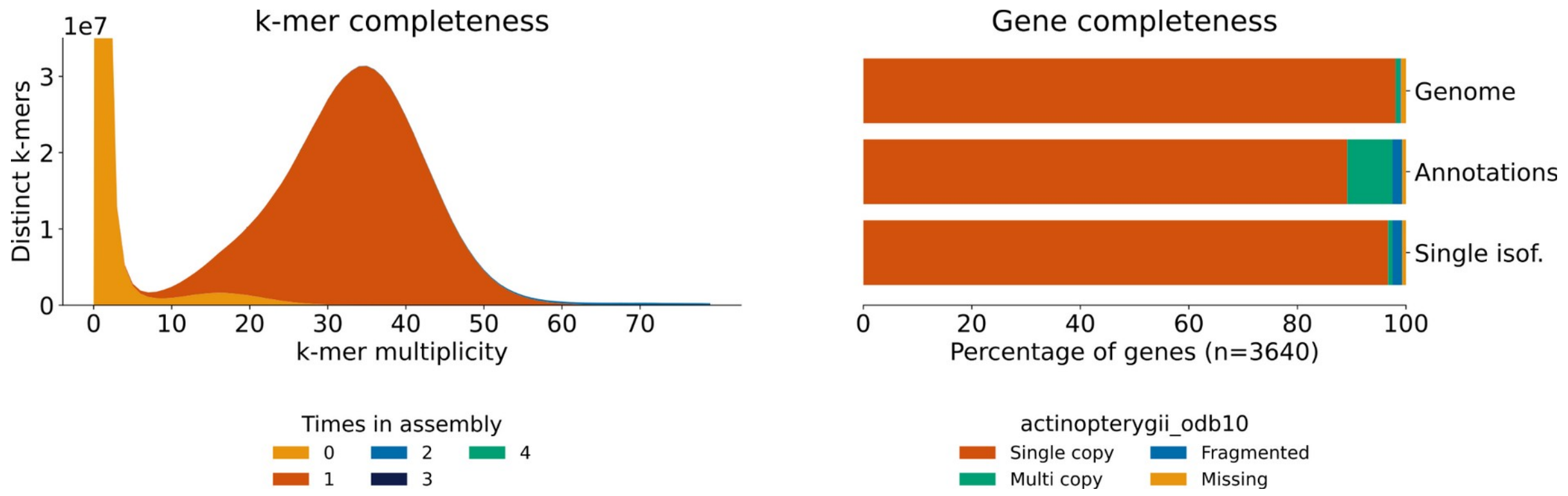

Supplementary figure S2. Assembly statistics of the 31-contig assembly. Kmer completeness (right) and BUSCO gene completeness assessment (left) of assembly and GALBA annotations.

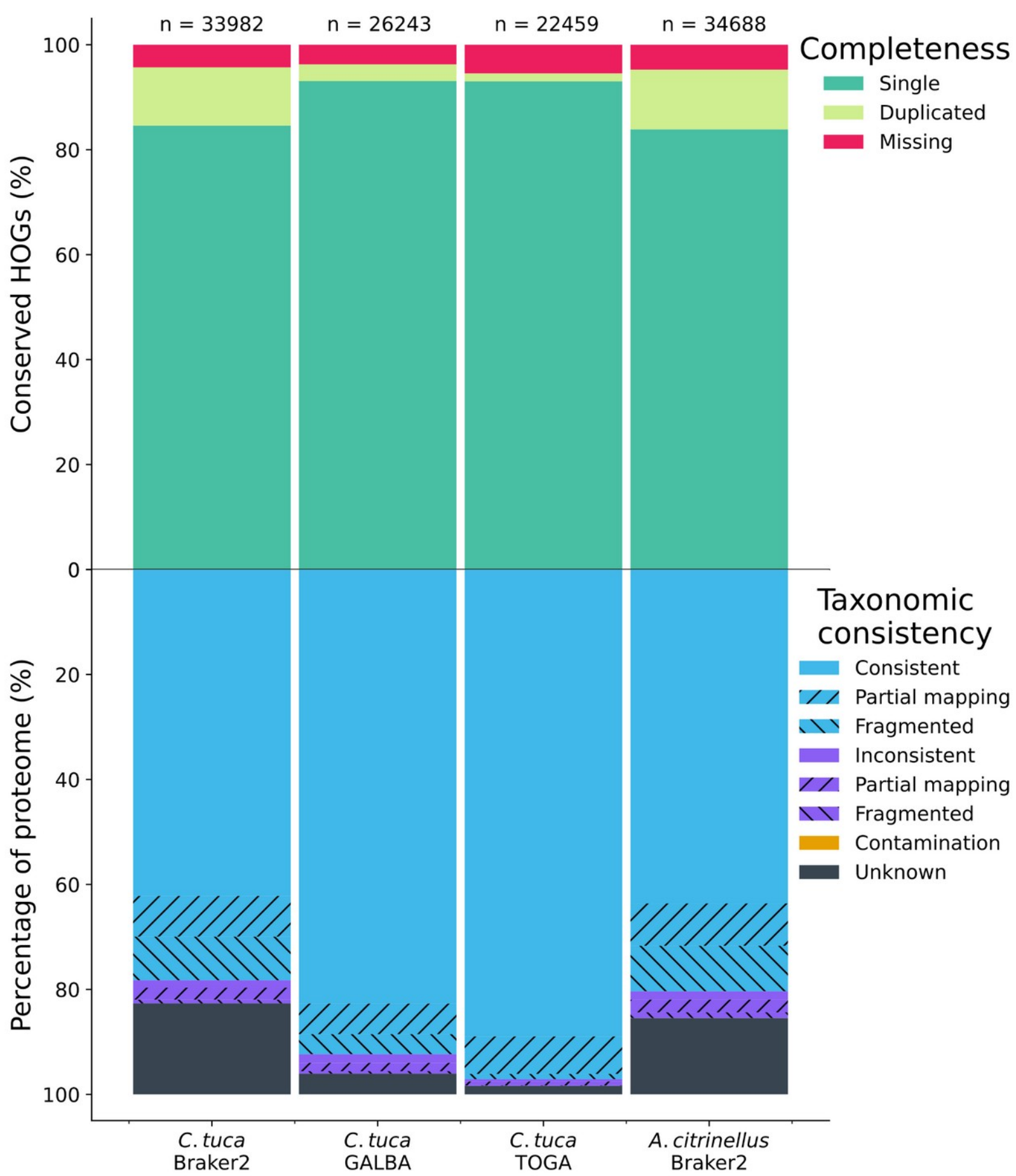

Supplementary figure S3. OMark assessment of C. *tuca* (31-contigs assembly) and *Amphilophus citrinellus* gene annotations using 17.922 Cichlidae conserved HOGs.

Supplementary Material

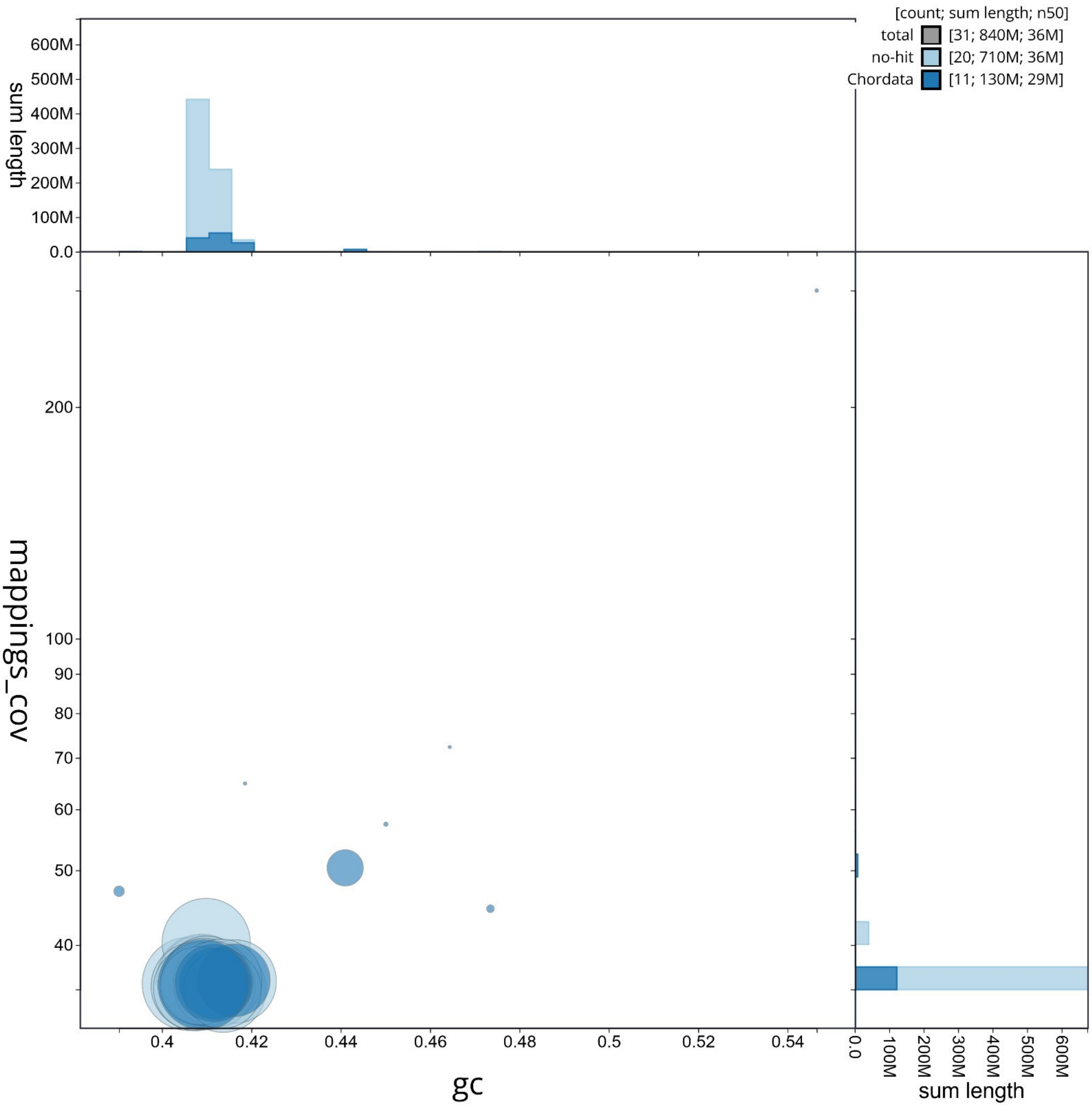

Supplementary figure S4. Blobtools taxonomy and coverage assessment. No contamination was detected.

Supplementary Material

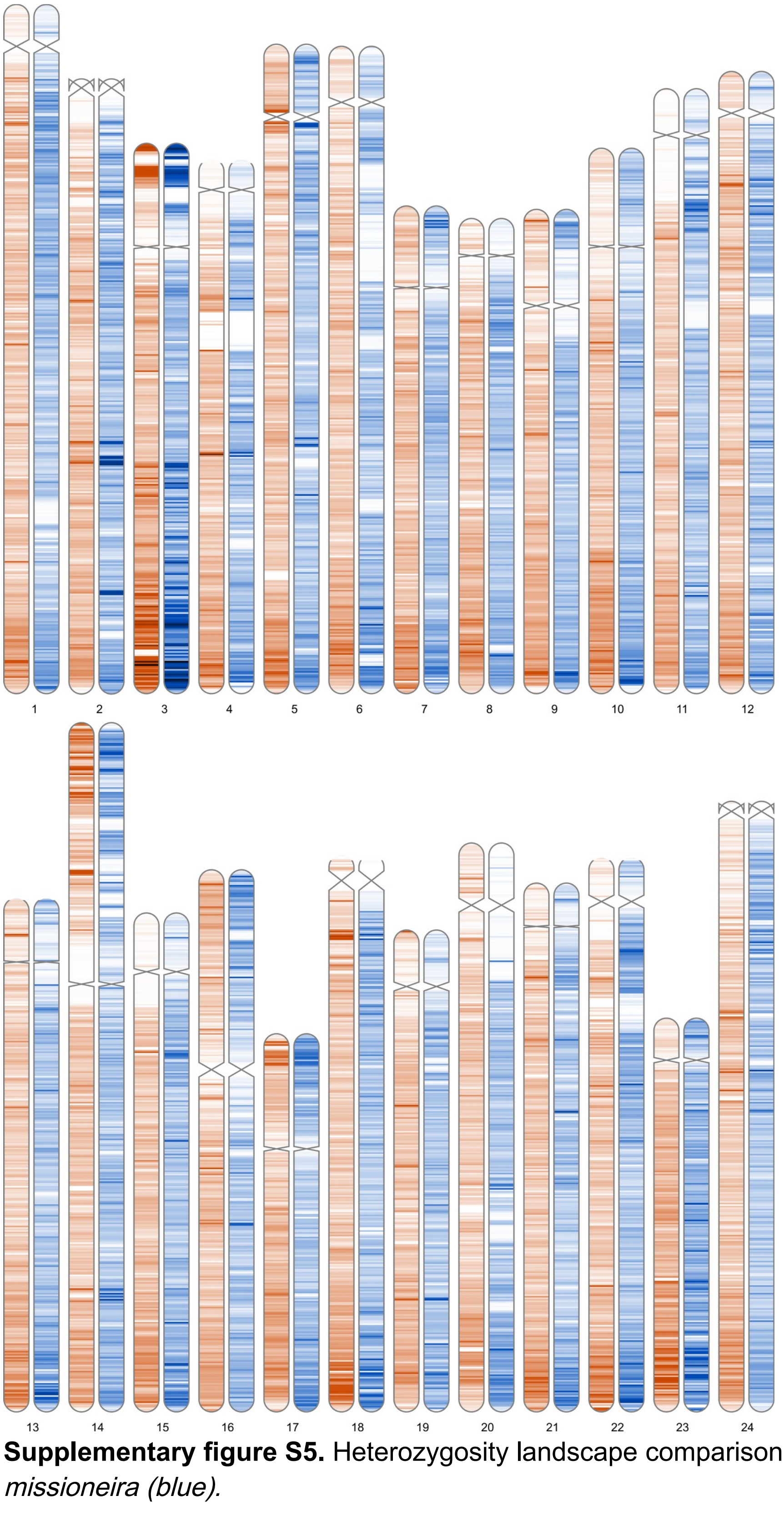

Supplementary figure S5. Heterozygosity landscape comparison between *C. tuca (red)* and *C. missioneira (blue).*

Supplementary Material

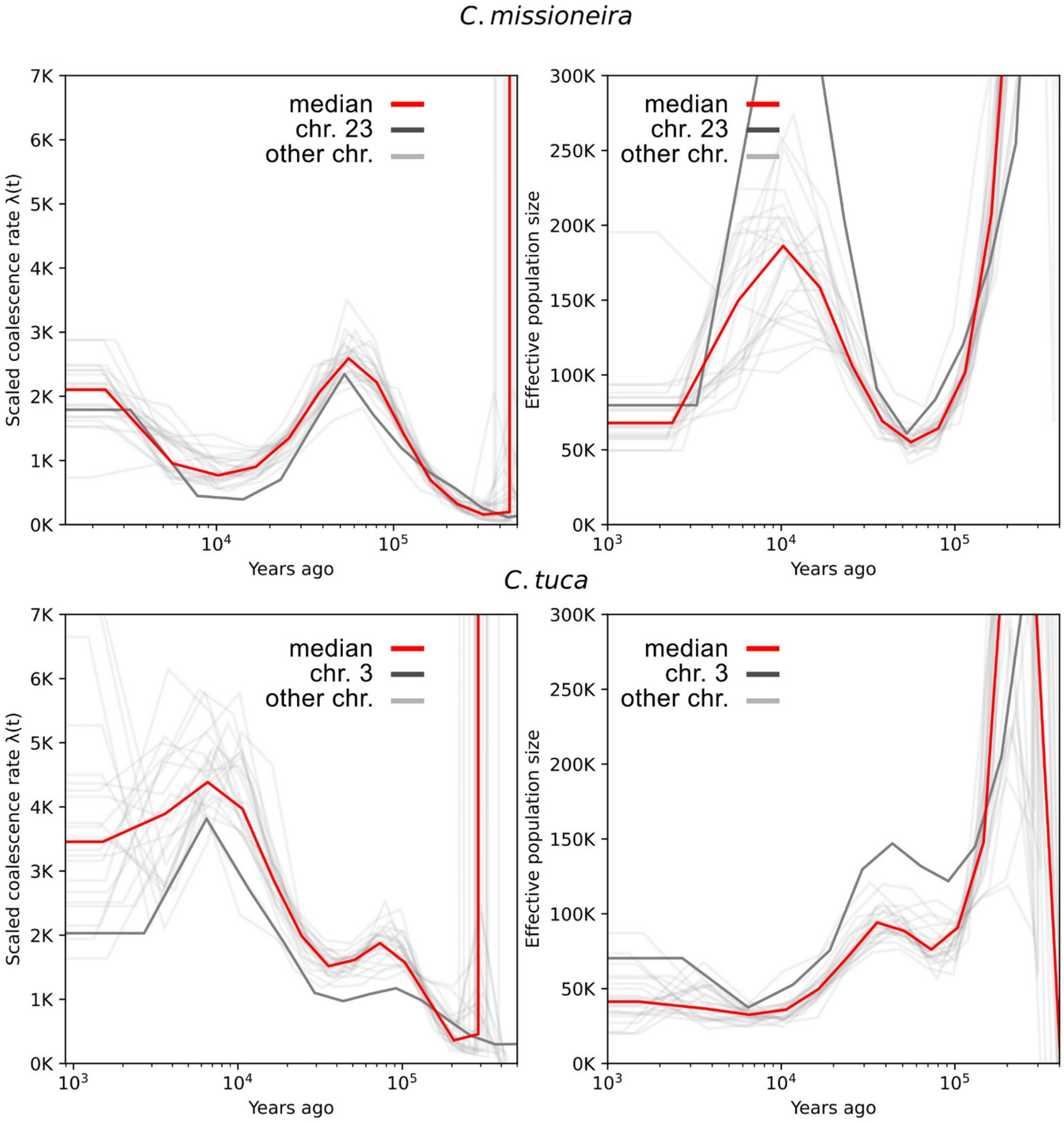

Supplementary figure S6. Coalescent rates for each chromosome estimated by multiple sequentially Markovian coalescent (MSMC) and historical demographic fluctuations estimated by assuming a 3 years generation time, 3.5e-9 mutation rate and panmixia.

Supplementary Material

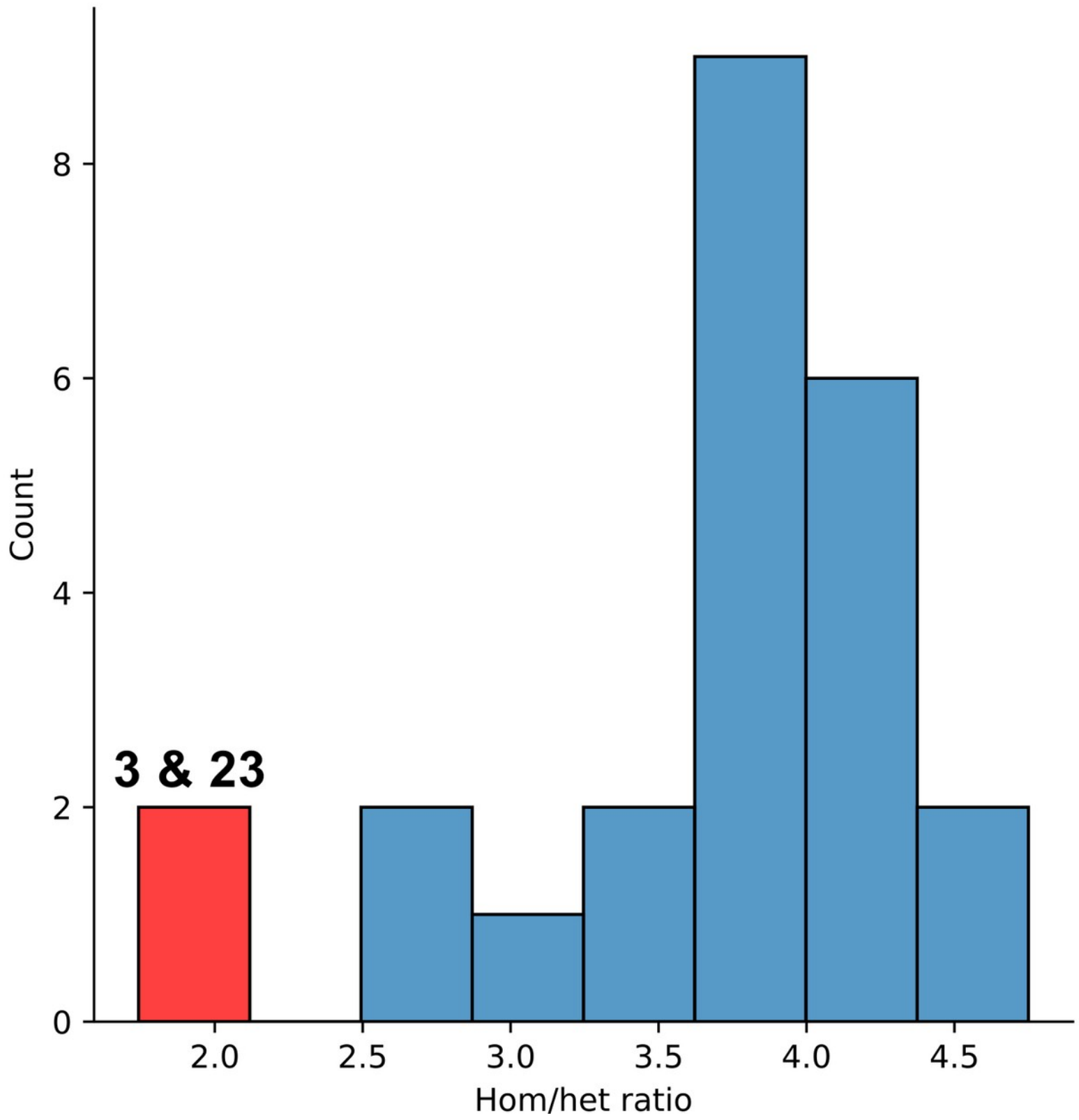

Supplementary figure S7. Distribution of the ratio between the normalized count of homozygous variants to heterozygous in *Crenicichla missioneira*. Chromosomes LG3 and LG23 are shown in red.

Supplementary Material

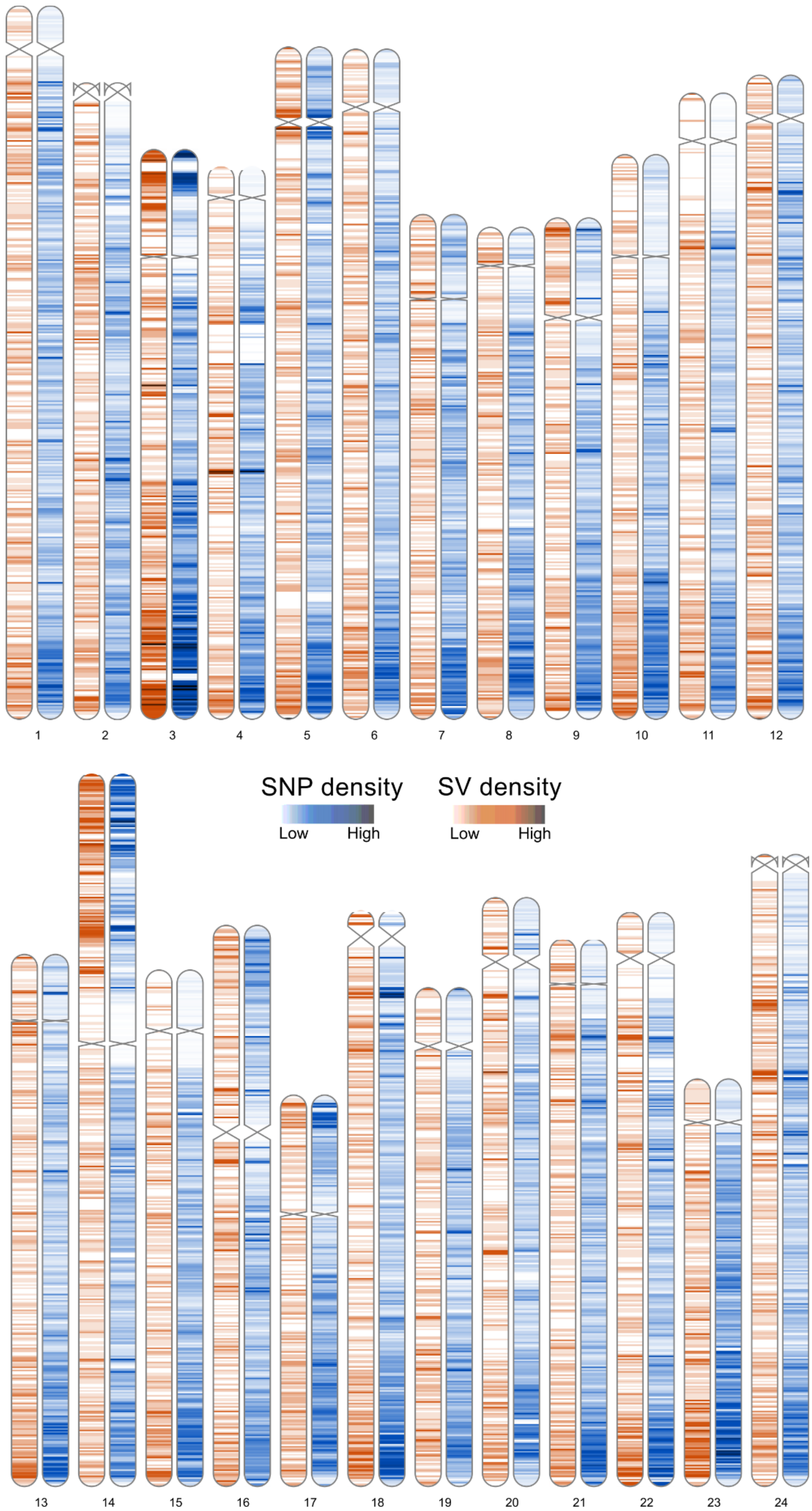

Supplementary figure S8. SV and SNP density across C. *tuca* chromosomes (LG).

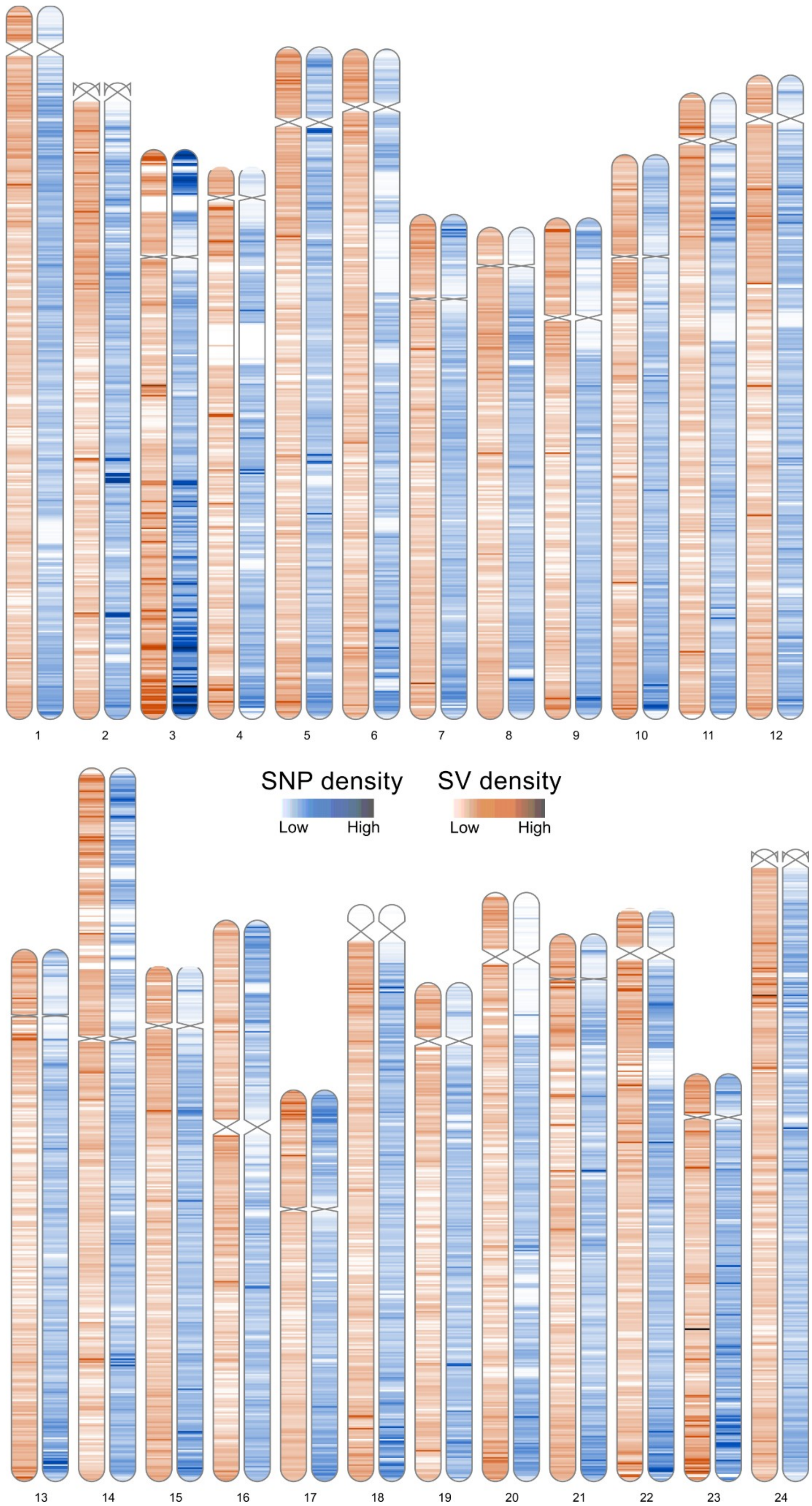

Supplementary figure S9. SV and SNP density across *C. missioneira* chromosomes (LG).

Supplementary Material

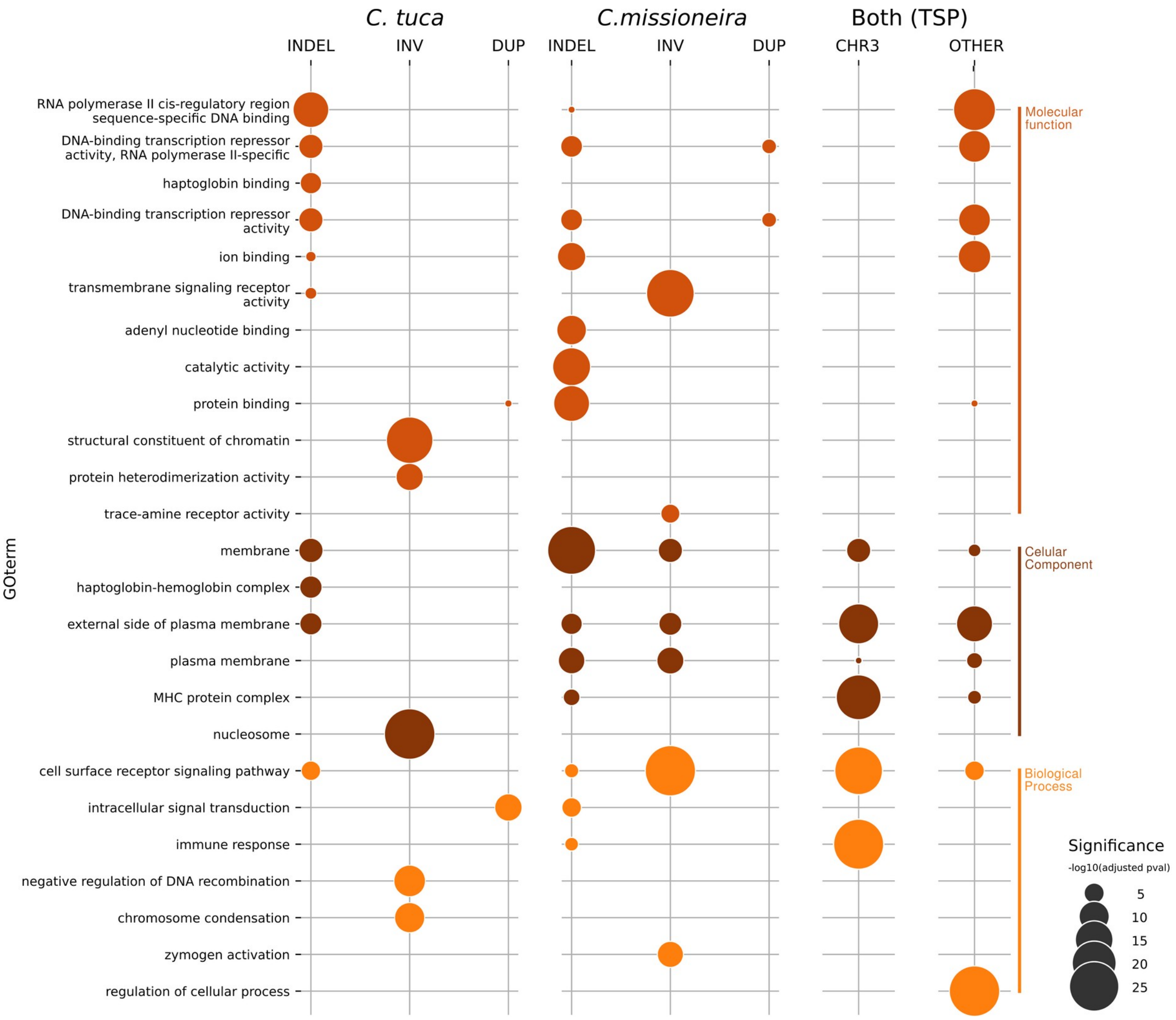

Supplementary figure S10. Functional enrichment of genes that are located closely to polymorphic SVs. Based on 1,303 (8% of svs) and 5,454 (13% of SVs) SVs close to genes in *C. tuca* and *C. missioneira* respectively.

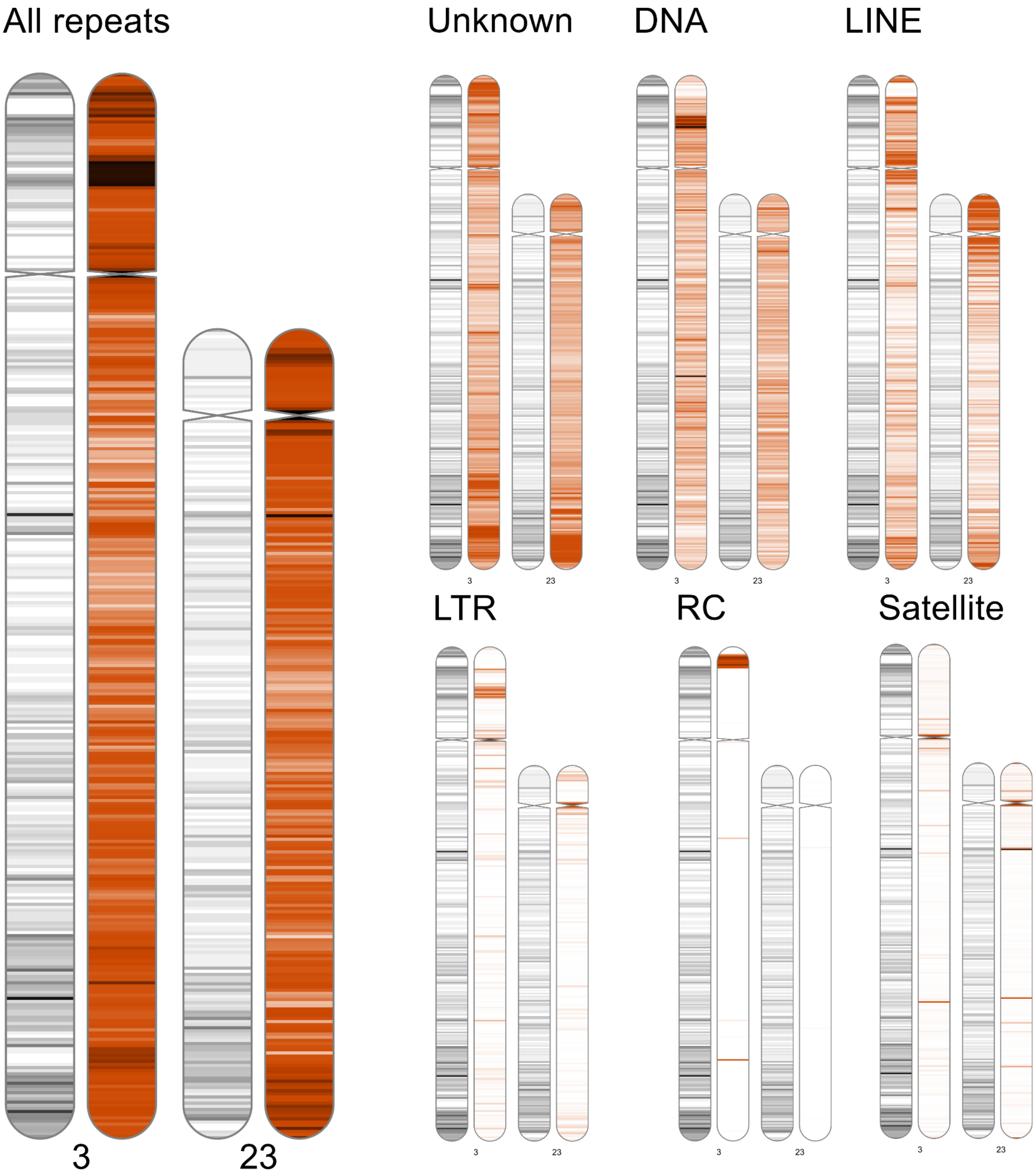

Supplementary figure S11. Distribution of SVs and repeats in *C. tuca* chromosomes LG3 and LG23. In gray, density of estimated polymorphic SVs; in red, density of different repeat types.

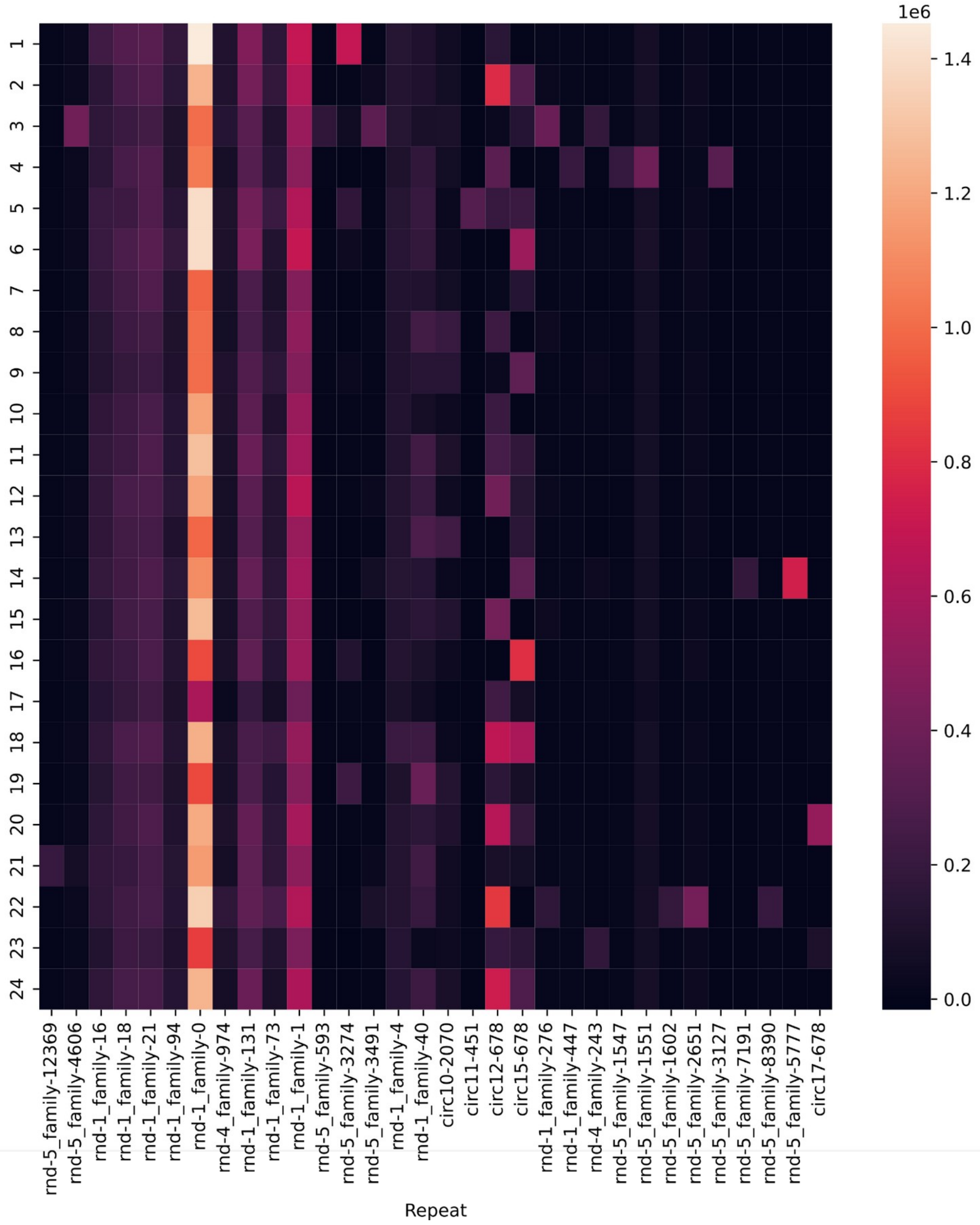

Supplementary figure S12. Heatmap of repeat abundance in each chromosome (LG). Repeats with more than 150,000 accumulated bases in at least one chromosome were plotted. These were selected for their potential use as cytogenetic markers. More information on Supplementary Note S5 and Table S1.

Supplementary Material

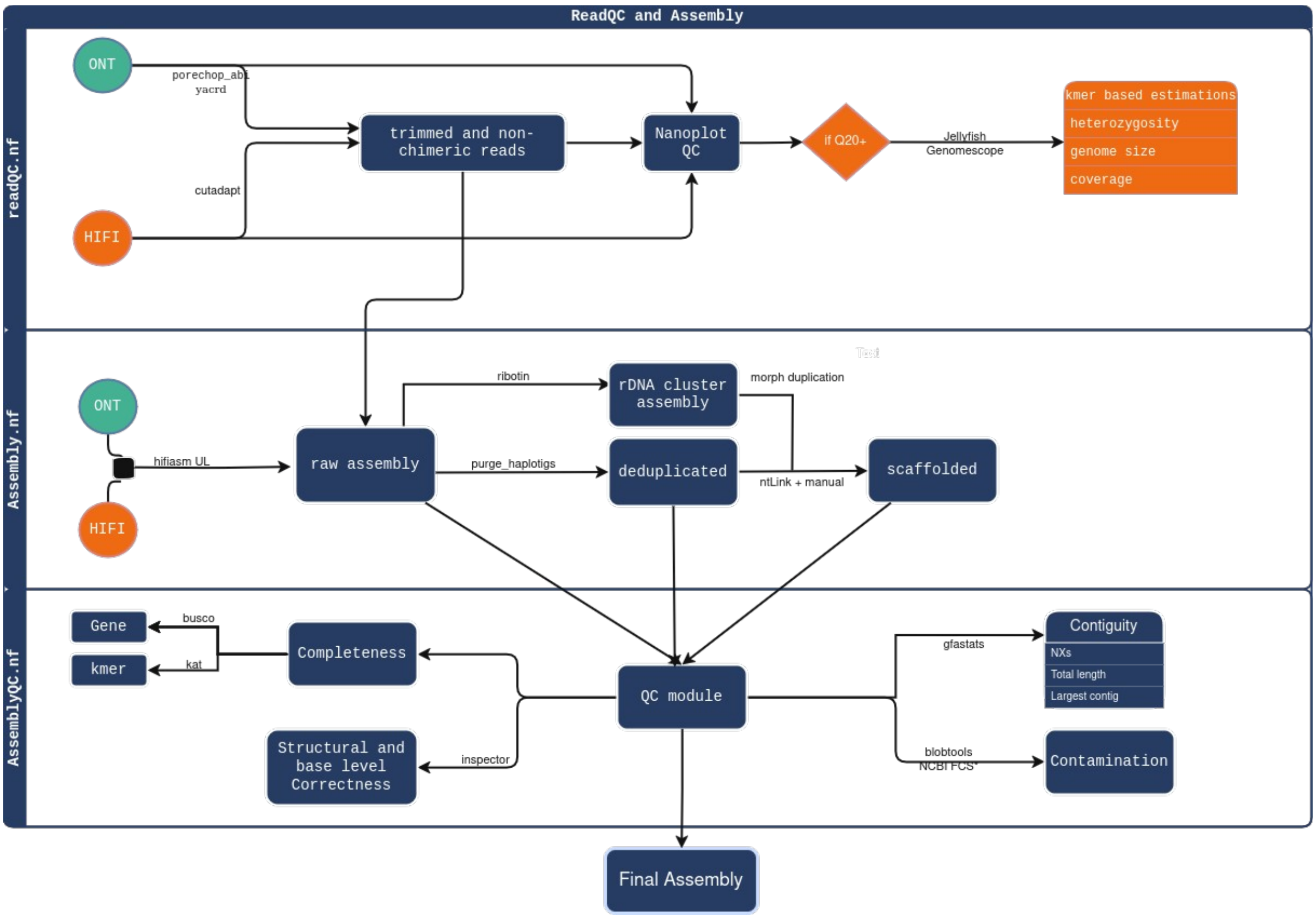

Supplementary figure S13. Assembly workflow.

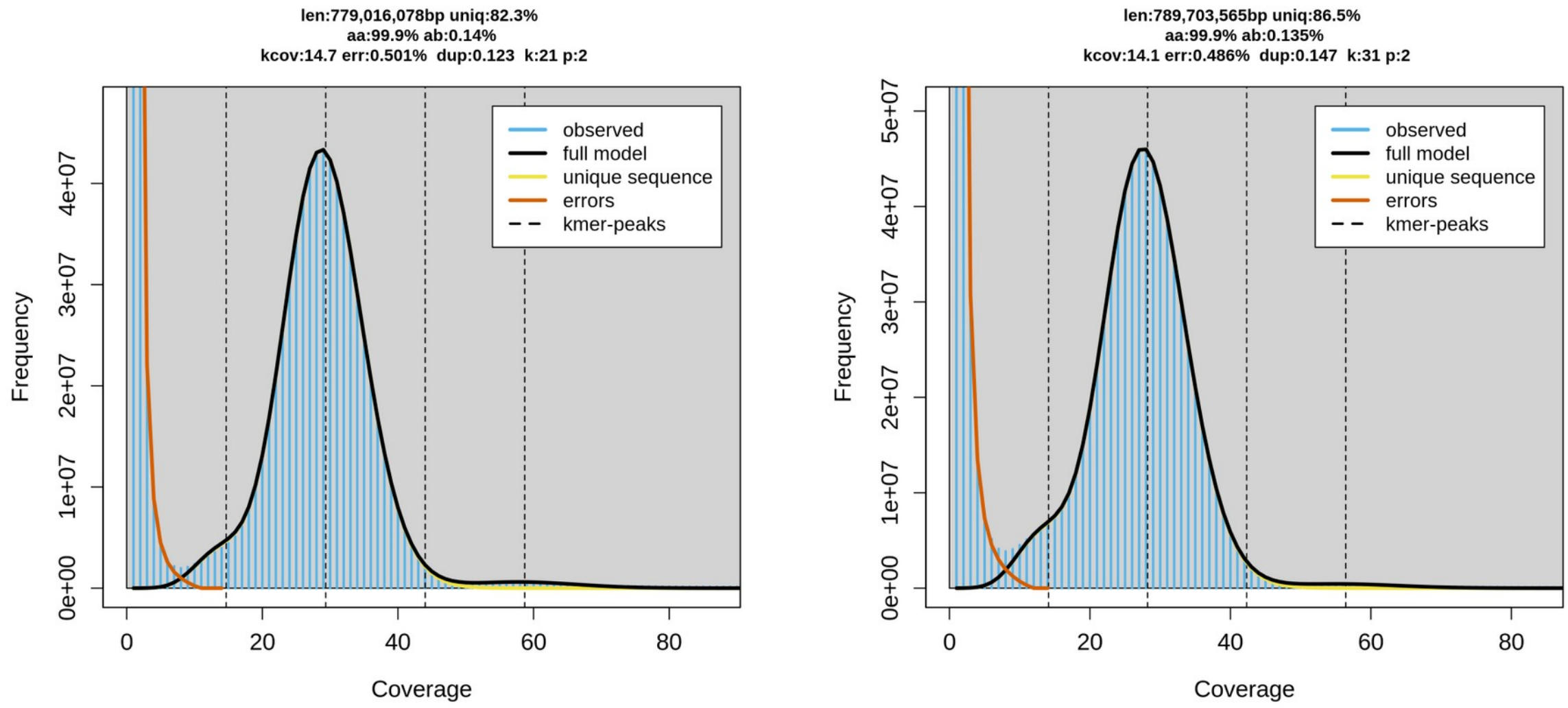

Supplementary figure S14. Genomescope profile including genome size estimates and heterozygosity. Run with kmer =21 (right) and kmer = 31 (left). Both models had an adequate fit to the kmer spectra.

Supplementary Material

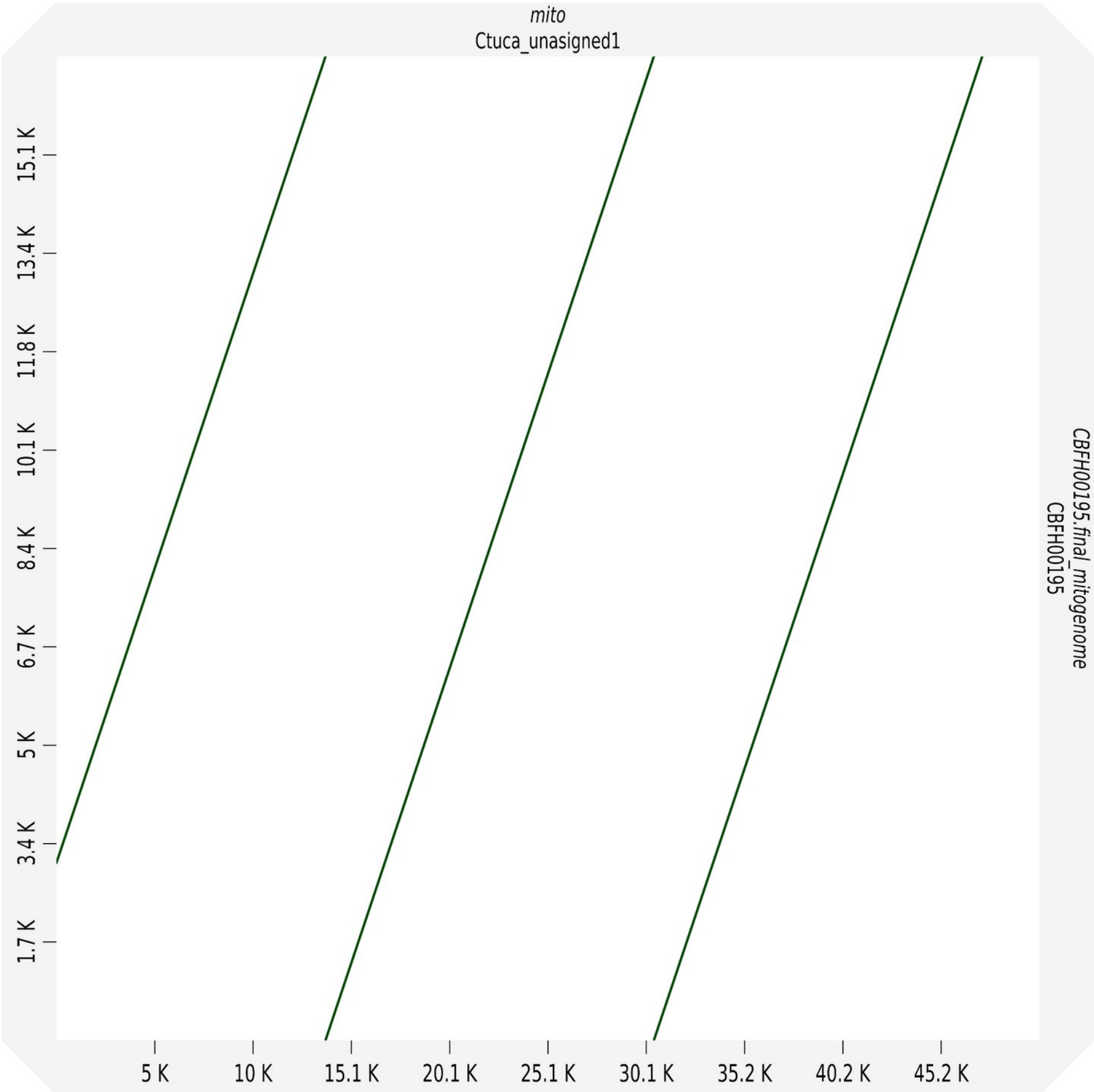

Supplementary figure S15. Detection and removal of artifactual mitochondrial contig.

Supplementary Material

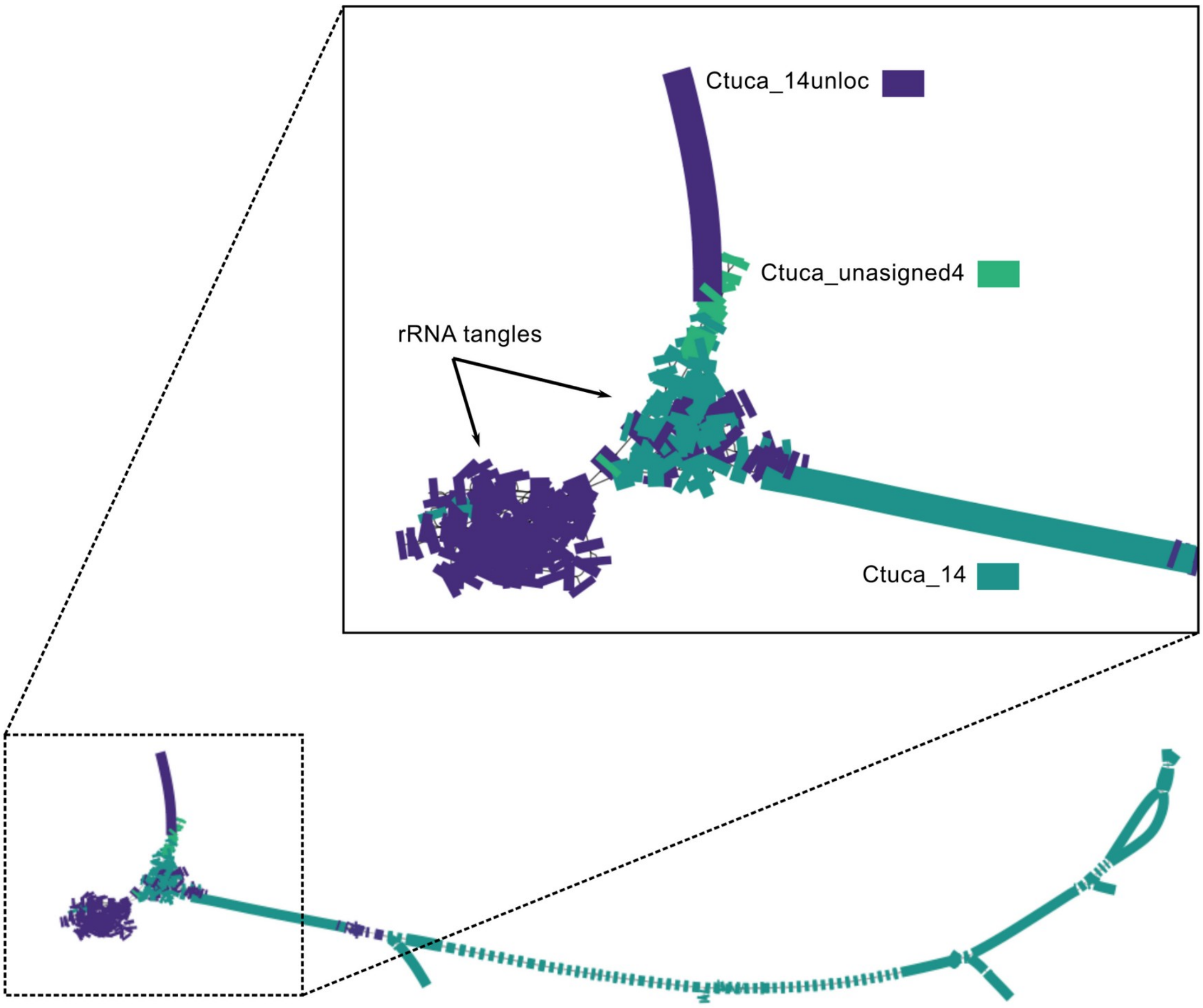

Supplementary figure S16. Visualization of LG14 assembly graph. Nodes in the hifiasm assembly graph (processed unitigs) were annotated with Dgenies association table.

Supplementary Material

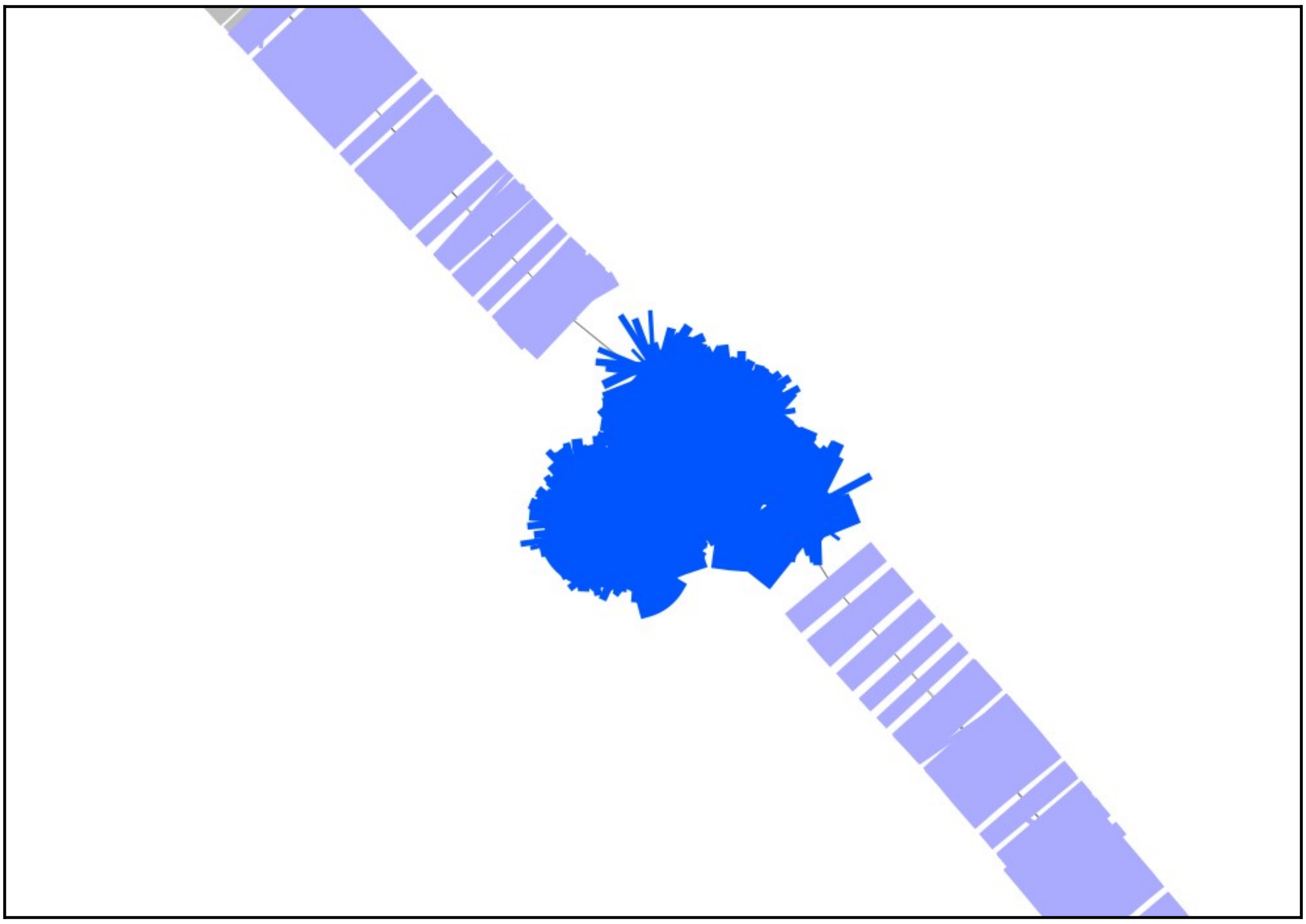

Supplementary figure S17. Tangles selected for ribosomal assembly with Ribotin.

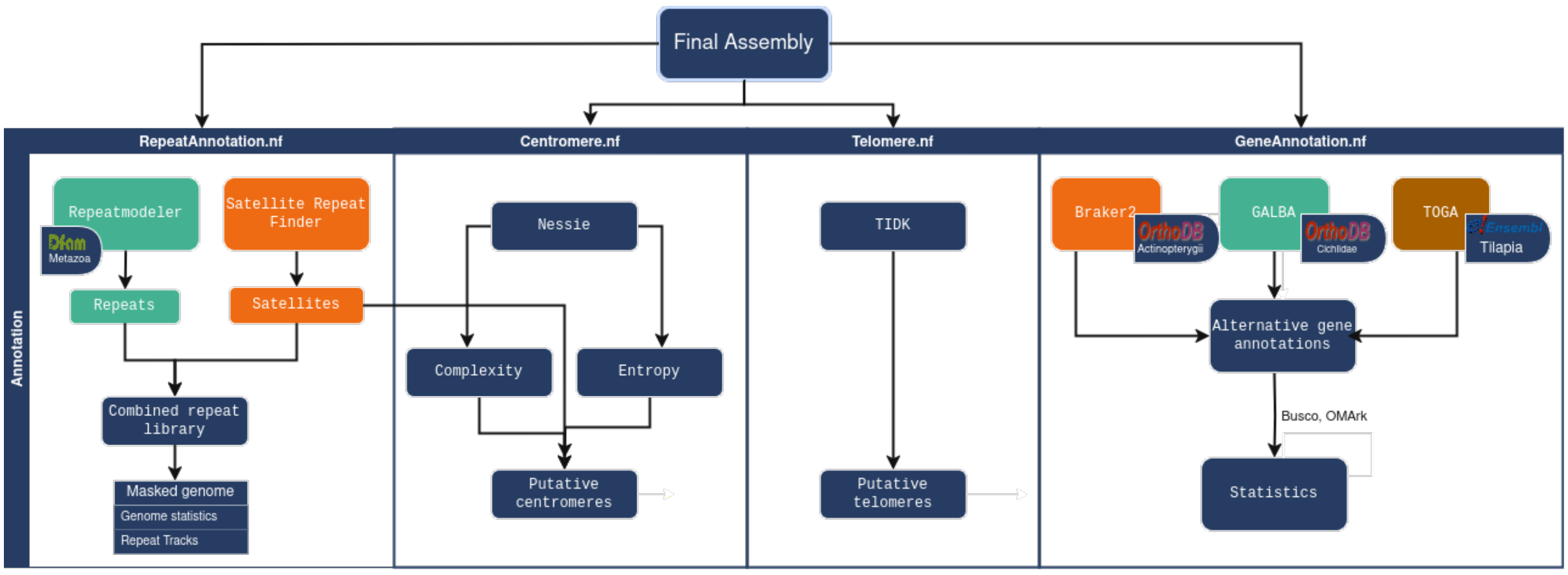

Supplementary figure S18. Annotation workflow.

Supplementary Material

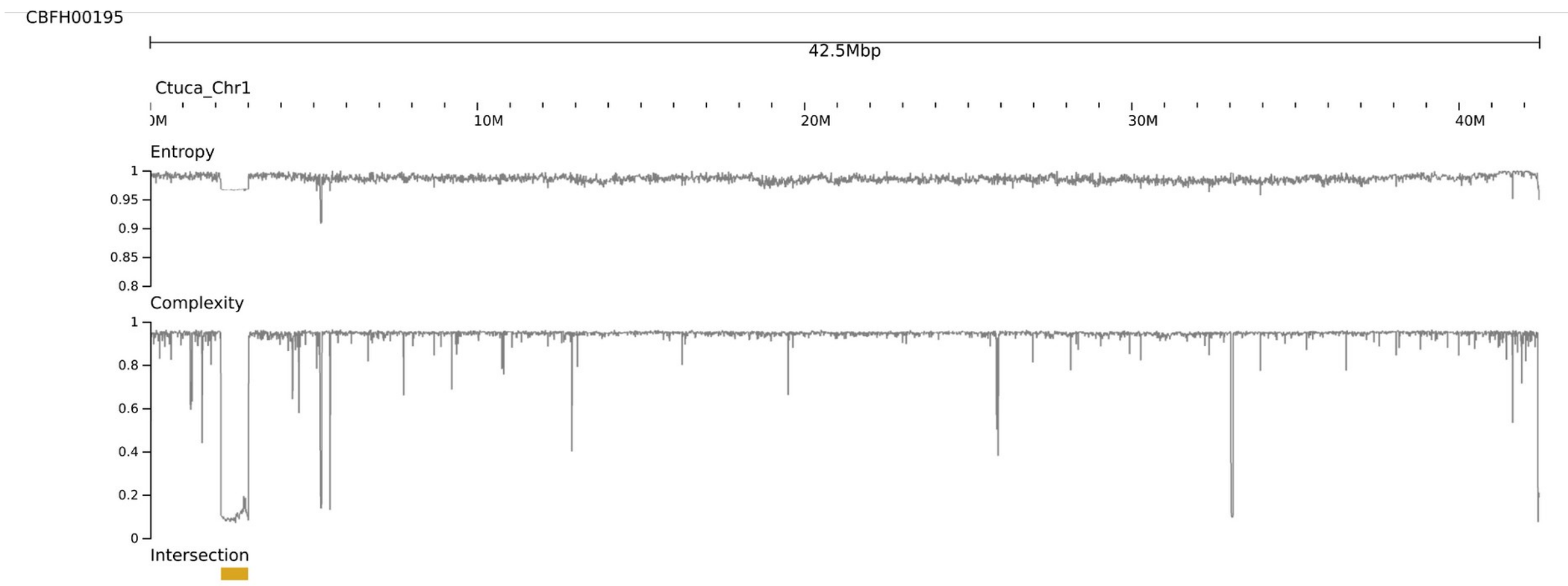

Supplementary figure S19. Example of centromere inference for LG1 from decreases in Linguistic complexity and sequence Entropy.

Supplementary tables

Supplementary table S1. Genomic characteristics of *Crenicichla tuca* pseudochromosomes. Assignment of chromosomes based on homology, morphology and cytogenetic markers. Differences to *Amphilophus citrinellus* chromosomes.

**Chrom. (LG)**	**Chr. assignment**		**Telomere**	**Centromere**	**Centromeric index**	**Morphology**	**Length (bp)**	**GC (%)**	**Cytogenetic marker**	**Repeat content**		**Differences to *A. citrinellus* (pb)**	
	A. citrinellus	Karyotype								**(%)**	**Length**	**Total length (excluding Ns)**	**Repeat length**
1	1	-	both	single region	6.1	A/T	42,463,988.00	40.6	Rnd-5_family-3274 #LTR/Pao	36.14	15,345,049.00	-579,840.00	-655,828.00
2	2	-	both	single region	1.5	A/T	37,898,467.00	40.7		37.77	14,313,411.00	366,977.00	37,123.00
3	3	-	both	single region	18.8	A/T	33,906,831.00	41.6	Rnd-1_family-276 #Unknown; rnd-5_family-4606 #DNA/CMC-EnSpm	42.96	14,565,284.00	-27,888,462.00	-21,476,815.00
4	4	-	both		5.8	A/T	32,949,993.00	41.3	Rnd-5_family-1551 #Unknown	37.62	12,398,172.00	-432,141.00	-182,215.00
5	5	4ST	both	multiple regions	-	-	40,011,898.00	40.9	5S	37.35	14,945,369.00	450,675.00	513,508.00
6	6	-	both	single region	8.6	A/T	39,885,017.00	40.9	-	37.43	14,927,572.00	-3,086,076.00	-2,486,050.00
7	7	-	both	single region	16.7	A/T	30,046,237.00	40.8	-	35.57	10,689,141.00	-2,519,877.00	-1,981,622.00
8	8	-	both	single region	7.8	A/T	29,276,336.00	41.2	-	36.05	10,553,529.00	-564,296.00	-656,573.00
9	9	-	both	single region	19.9	A/T	29,836,682.00	41.4	-	37.26	11,115,720.00	-2,320,593.00	-1,743,781.00
10	10	-	both	single region	19.9	A/T	33,604,528.00	40.9	-	35.78	12,025,629.00	-2,029,793.00	-1,740,587.00
11	11	-	both	single region	7.7	A/T	37,269,310.00	41.1	-	35.73	13,315,044.00	-2,977,306.00	-2,211,152.00
12	12	-	both	multiple regions	-	-	38,335,167.00	40.9	-	35.15	13,475,759.00	-481,612.00	-350,710.00
13	13	-	both	single region	12.5	A/T	33,643,704.00	40.9	-	34.07	11,463,368.00	82,293.00	227,351.00

NA= not applicable; - = undetected; A/T = acrocentric, telocentric; M/SM = metacentric, submetacentric; * not possible to calculate CI but morphology estimated based on cytogenetic assignment

Chrom. (LG)	Chromosome assignment		Telomere	Centromere	Centromeric index	Morphology	Length (bp)	GC (%)	Cytogenetic marker	Repeat content		Differences to *A. citrinellus* (pb)	
	A. citrinellus	Karyotype								Percentage	Length	Total length (excluding Ns)	Repeat length
14	14	1M	both; interstitial	multiple regions	*	M/SM	45,114,477.00	41.6	5.8S, 18S, 28S; rnd-5_family-5777 #LINE/L1-Tx1; rnd-5_family-7191 #Unknown	43.28	19,527,166.00	3,273,981.00	2,546,258.00
15	15	-	both	single region	11.8	A/T	32,657,047.00	41.2	-	38.2	12,474,388.00	-1,126,196.00	-982,517.00
16	16	-	both	single region	36.9	M/SM	35,484,944.00	40.8	-	35.67	12,659,287.00	-1,352,007.00	-1,188,510.00
17	17	-	both	single region	30.4	M/SM	24,750,365.00	41.2	-	32.59	8,064,829.00	-1,234,770.00	-1,076,520.00
18	18	-	both	single region	4.6	A/T	36,467,619.00	41.0	-	38.58	14,070,557.00	-1,052,242.00	-913,345.00
19	19	-	both	single region	11.7	A/T	31,540,754.00	41.2	-	34.42	10,856,609.00	742,339.00	394,102.00
20	20	-	both	multiple regions	-	-	37,237,399.00	41.2	circ17-678	36.66	13,650,050.00	126,108.00	273,311.00
21	21	-	both	single region	8.2	A/T	34,613,853.00	40.9	rnd-5_family-12369	33.89	11,730,898.00	-60,922.00	-291,068.00
22	22	-	both	single region	8.0	A/T	36,299,731.00	41.4	Rnd-5_family-2651 #Unknown	43.06	15,633,098.00	2,143,054.00	2,007,632.00
23	23	-	both	multiple regions	-	-	25,772,004.00	41.6	-	42.5	10,955,814.00	-6,382,640.00	-5,738,987.00
24	24	-	both	single region	1.6	A/T	39,965,629.00	40.9	-	36.85	14,727,093.00	1,699,672.00	1,130,492.00
MT	MT	NA	NA		NA	NA	16794	46.4	-	2.11			
unasigned2	-	-	-		-	-	543130	39.0	Multiple repeats (121); inmunoglobulin	45.72			
unasigned5	-	-	-		-	-	95322	45.0	Satellites (4)	99.97			
unasigned6	-	-	-		-	-	58529	41.9	rnd-5_family-3168; circ23-2210;	99.98			

NA= not applicable; - = undetected; A/T = acrocentric, telocentric; M/SM = metacentric, submetacentric; * not possible to calculate CI but morphology estimated based on cytogenetic assignment

Supplementary table S2. Genetic distance between *C. tuca* and *C. missioneira*.

**Chromosome (LG)**	**Homozygous SNPs**	**Callable sites**	**Uncorrected distance**	**Jukes–Cantor distance**
1	468406	38255193	0.0122	0.0123
2	421955	33404168	0.0126	0.0127
3	347971	28403168	0.0123	0.0124
4	331774	27239000	0.0122	0.0123
5	434300	35395065	0.0123	0.0124
6	453232	35534029	0.0128	0.0129
7	346507	27419926	0.0126	0.0127
8	332466	26193867	0.0127	0.0128
9	316351	26197871	0.0121	0.0122
10	384734	30663157	0.0125	0.0127
11	397551	32901675	0.0121	0.0122
12	419365	33998231	0.0123	0.0124
13	368817	30446081	0.0121	0.0122
14	456467	36981803	0.0123	0.0124
15	356388	28709024	0.0124	0.0125
16	376298	31223620	0.0121	0.0121
17	291463	22856622	0.0128	0.0129
18	380177	31080819	0.0122	0.0123
19	326832	27958024	0.0117	0.0118
20	389964	32647827	0.0119	0.0120
21	374138	31197187	0.0120	0.0121
22	375379	30151471	0.0124	0.0126
23	293273	22676867	0.0129	0.0130
24	430411	34842948	0.0124	0.0125
**Total**	**9074219**	**736377643**	**0.0123**	**0.0124**

Supplementary table S3. Count and length distribution of different SV types in *Cichlidae*. Note that variant types are not comparable across. methods.

Species	Method	Type	Count	Length distribution					Total Length
				min.	Q1	Q2	Q3	max.	
C. tuca* x *C. tuca	**read-based**	**DUP**	1						26,770
		**INDEL**	16,110	1	1	55	213	207,661	15,059,992
		**INV**	24	95	364	929	2,325	113,659	150,728
		**Total**	16,135						
C. missioneira* x *C. tuca	**read-based**	**DUP**	37	59	1,831	12,289	31,780	4,939,936	5,691,069
		**INDEL**	189,456	1	1	1	186	4,890,336	136,196,121
		**INV**	286	63	540	1,331	3,242	18,385,888	52,595,967
		**Total**	189,779						194,483,157
C. tuca* x *A. citrinellus	**assembly-based**	**DUP**	12,710	14,132	7,937,950	23,105,555	29,788,929	44,976,474	17,651,612
		**INV**	268	83,091	7,404,812	20,469,251	29,112,405	42,716,521	152,070,663
		**TRANS**	6,993	14,132	7,503,098	24,252,163	31,420,018	45,027,539	54,799,094
		**Total**	19,971						224,521,369
A. citrinellus* x *O. niloticus	**assembly-based**	**DUP**	899	5,966	10,373,032	16,043,085	28,331,165	61,632,422	4,697,014
		**INV**	178	12,151	8,117,581	22,095,160	30,511,971	60,599,365	153,244,460
		**TRANS**	9,250	267	9,559,442	28,039,434	33,841,980	62,217,656	27,221,648
		**Total**	10,327						185,163,122

Supplementary table S4. Total count of structural variants per chromosome, discriminating homozygous and heterozygous genotypes. Normalization was performed by chromosome length.

**Chromosome (LG)**	***C. tuca***		***C. missioneira***				
	**Heterozygous**		**Heterozygous**		**Homozygous**		**Hom / het ratio**
	**Raw count**	**Normalized***	**Raw count**	**Normalized**	**Raw count**	**Normalized**	
**1**	650	1.50E-05	1840	4.30E-05	7334	1.73E-04	4.0
**2**	604	1.60E-05	1788	4.70E-05	6873	1.81E-04	3.8
**3**	1465	**4.30E-05**	3506	**1.03E-04**	6109	**1.80E-04**	**1.7**
**4**	519	1.60E-05	1516	4.60E-05	5182	1.57E-04	3.4
**5**	751	1.90E-05	1951	4.90E-05	7162	1.79E-04	3.7
**6**	612	1.50E-05	1541	3.90E-05	7320	1.84E-04	4.8
**7**	512	1.70E-05	1384	4.60E-05	5504	1.83E-04	4.0
**8**	464	1.60E-05	1197	4.10E-05	5314	1.82E-04	4.4
**9**	514	1.70E-05	1285	4.30E-05	5229	1.75E-04	4.1
**10**	556	1.70E-05	1547	4.60E-05	6229	1.85E-04	4.0
**11**	521	1.40E-05	1557	4.20E-05	6503	1.74E-04	4.2
**12**	695	1.80E-05	1839	4.80E-05	6770	1.77E-04	3.7
**13**	583	1.70E-05	1434	4.30E-05	5854	1.74E-04	4.1
**14**	1015	2.20E-05	2679	5.90E-05	7575	1.68E-04	2.8
**15**	504	1.50E-05	1658	5.10E-05	6131	1.88E-04	3.7
**16**	671	1.90E-05	1591	4.50E-05	6155	1.73E-04	3.9
**17**	407	1.60E-05	1050	4.20E-05	4380	1.77E-04	4.2
**18**	692	1.90E-05	1849	5.10E-05	5898	1.62E-04	3.2
**19**	496	1.60E-05	1317	4.20E-05	4967	1.57E-04	3.8
**20**	601	1.60E-05	1483	4.00E-05	6369	1.71E-04	4.3
**21**	548	1.60E-05	1611	4.70E-05	6125	1.77E-04	3.8
**22**	681	1.90E-05	2487	6.90E-05	6338	1.75E-04	2.5
**23**	701	**2.70E-05**	2406	**9.30E-05**	4777	**1.85E-04**	**2.0**
**24**	735	1.80E-05	2021	5.10E-05	7144	1.79E-04	3.5
Total	15497	N/A	42537	N/A	147242	N/A	N/A
Mean	N/A	1.85E-05	N/A	5.11E-05	N/A	1.76E-04	3.65

N/A = Not applicable

Supplementary table S5. Count of shared and exclusive structural variants per chromosome. * Indicates putative trans-species polymorphism.

Chromosome (LG)	*C. tuca*		*C. missioneira*			
	Heterozygous		Heterozygous		Homozygous	
	Exclusive	Shared	Exclusive	Shared *	Exclusive	Shared
**1**	447	203	1,814	26	7,155	177
**2**	401	203	1,760	28	6,691	175
**3**	**1,075**	**390**	**3,373**	**132**	**5,842**	**258**
**4**	319	200	1,472	42	5,020	158
**5**	529	222	1,922	29	6,962	193
**6**	423	189	1,523	18	7,137	171
**7**	359	153	1,359	25	5,373	128
**8**	331	133	1,175	20	5,197	113
**9**	359	155	1,260	25	5,098	130
**10**	374	182	1,527	20	6,064	162
**11**	373	148	1,537	20	6,373	128
**12**	501	194	1,810	29	6,602	165
**13**	399	184	1,408	26	5,695	158
**14**	779	236	2,636	43	7,377	193
**15**	348	156	1,636	22	5,995	134
**16**	457	214	1,562	29	5,966	185
**17**	275	132	1,042	8	4,253	124
**18**	487	205	1,808	41	5,730	164
**19**	338	158	1,301	16	4,824	142
**20**	411	190	1,449	34	6,213	156
**21**	381	167	1,584	27	5,981	140
**22**	481	200	2,442	45	6,180	155
**23**	469	232	2,345	60	4,604	172
**24**	531	204	1,977	44	6,977	160
**Total**	**10,847**	**4,650**	**41,722**	**809**	**143,309**	**3,841**

Supplementary table S6. Comparison of *C. tuca* and VGP cichlid assemblies.

**Species**	**Accession**	**Sequencing technology (coverage)**	**N contigs**	**N scaffolds**	**Genome length (pb)**	**Gaps (pb)**	**Unplaced (pb)**	**28S**		**5S**	
								**Total copies (n. contigs)**	**Copies in the largest cluster**	**Total copies (n. contigs)**	**Copies in the largest cluster**
Amphilopus citrinellus	GCA_013435755.1	PacBio Sequel CLR; Bionano Genomics; Phase Genomics HiC	8,623	1,391	961,214,450	30720055	111,835,131	no complete copies	no complete copies	850 (24)	696
Archocentrus centrarchus	GCF_007364275.1	PacBio Sequel I; 10X Genomics linked reads; Bionano Genomics DLS; Arima Genomics Hi-C. (76X unespecified)	926	188	932930362	16903477	104,587,794	2 (2)	1	219 (42)	36
Rhamphochromis sp. 'chilingali'	GCA_963969265.1	PacBio (64X) and Arima2 Hi-C	503	81	921167628	84400	1,992,477	4 (4)	1	1227 (19)	1009
Aulonocara stuartgranti	GCA_965226115.1	ONT Duplex (18X); HiC	53	22	945366440	4300	0	43 (5)	38	1365 (18)	1085
Crenicichla tuca	this work	Pacbio Hifi (30X); Nanopore UL (30X)	29	27	839,728,961	2000*	696,981	48 (3)	45	820 (24)	694

Supplementary notes

Supplementary note S1. Genome assembly and manual curation

Assembly and duplicate purging:

Assembly with Hifiasm produced 74 sequences totaling 841 Mbp. After duplicate purging, 33 sequences remained (839 Mbp). These were renamed based on their correspondence to *A. citrinellus* chromosomes or labeled as unassigned.

To verify the duplicate purging step, dotplot alignments of *Purge_haplotigs* were manually examined. Most removed contigs were marked as "REPEATS," aligning with high coverage to *Ctuca_14_unloc* (29 sequences) and, to a lesser extent, *Ctuca_14* (30 sequences). This suggests that many misassemblies were associated with the rRNA cluster on this chromosome. Additional *Purge_haplotigs* repeats corresponded to: Ctuca_19, (1 seq.), Ctuca_15 (1 seq.), Ctuca_21 (2 seq.), Ctuca_8 (2 seq.). Six remaining sequences were identified as uncollapsed haplotypes (haplotigs) of Ctuca_4, Ctuca_6, Ctuca_24, Ctuca_2, Ctuca_8 and Ctuca_19.

Scaffolding of fragmented chromosomes:

To further refine the assembly, NtLink was used to scaffold Ctuca_6 and Ctuca_14_unloc with ultralong ONT reads, reducing the genome to 31 sequences (839 Mbp). At this step, chr 14 remained fragmented in more than two parts due to the presence of repeats, especially rDNA arrays. It consisted of: (i) the main scaffold (38 Mbp) corresponding to a raw Hifiasm contig; (ii) a ~6 Mb scaffold generated from the scaffolding of two smaller contigs; and (iii) two short unassigned contigs (*unassigned_3* and *unassigned_4*). . We found copies of 18S, 5.8 and 28S in the main contig 14 (7), scaffold 14_unloc (11) and unasigned_4 (2). Gene and repeat annotations were carried on in this assembly and later transferred to the final assembly with minimap2 and liftoff.

To reconstruct the correct order of the rRNA-bearing scaffolds and the rRNA cluster, the Hifiasm assembly graph (p_utg) was visualized in BandageNG and annotated with contigs from the 31-sequence assembly (Supplementary figure S16). rRNA copies located at the ends of Ctuca_14 and Ctuca_14_unloc were trimmed and replaced with 1,000 Ns. The Ctuca_14 contig was then joined to the rRNA cluster model, and both were connected to Ctuca_14_unloc in an inverted and complemented orientation, with Ns maintained as spacers. Ctuca_unassigned4 was removed from the assembly because it was found repeated multiple times in tandem within Ctuca_14_unloc, suggesting either a misassembled repeat or an uncollapsed haplotig. Similarly, unassigned3, a contig composed mainly of low-complexity repeats, was excluded as it was fully covered by Ctuca_14. This manual curation and scaffolding increased the length of chromosome 14 from 38,232,034 bp (Hifiasm contig) to 45,114,477 bp.

The final assembly was represented by a count of 27 nuclear sequences and the mitochondrial genome. The remaining unassigned contigs contained inmunoglobulins (unasigned2), and completely by repeats (>99.9 repeat content, unasigned5 and unasigned6).

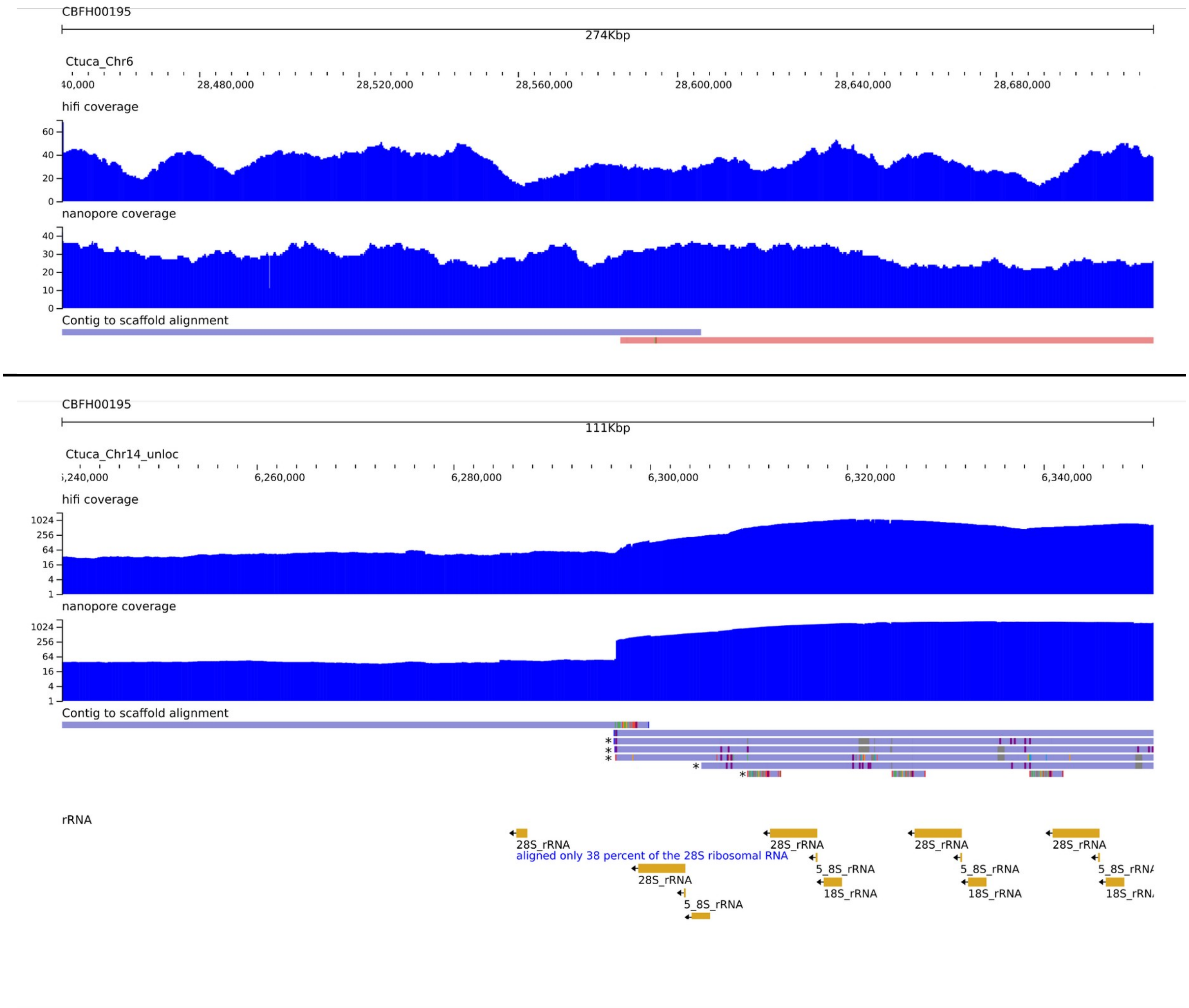

Supplementary figure S20. Visualization of scaffolding of Scaffold 6 (above) and Scaffold 14_unloc (below). Asterisks denote supplementary alignments of contig to Scaffold 14_unloc. Note the high coverage (log scale) of a region corresponding to the rRNA cluster near the scaffolding site.

Assessment of chromosome ends:

To assess whether all chromosomes were assembled to their ends, we recovered UL reads mapping to the last 30 kb of both the 5′ and 3′ termini. Reads were reverse complemented when necessary, and the density of telomeric repeats was estimated in 10 kb windows from each read end using TIDK. For each chromosome end, the mean density of telomeric repeats and its 95% confidence interval were plotted (Supplementary figures S21 and S22). A clear peak was observed in the first windows of all chromosomes, corresponding to telomeres and generally spanning up to five windows (~50 kb). In some chromosomes, an adjacent region with lower but detectable telomeric content extended rarely beyond ten windows; these were considered subtelomeric regions, followed by a marked decline toward the genome-wide background density of telomeric repeats. We complemented this approximation by running TIDK directly on pseudochromosomes as described in the main text.

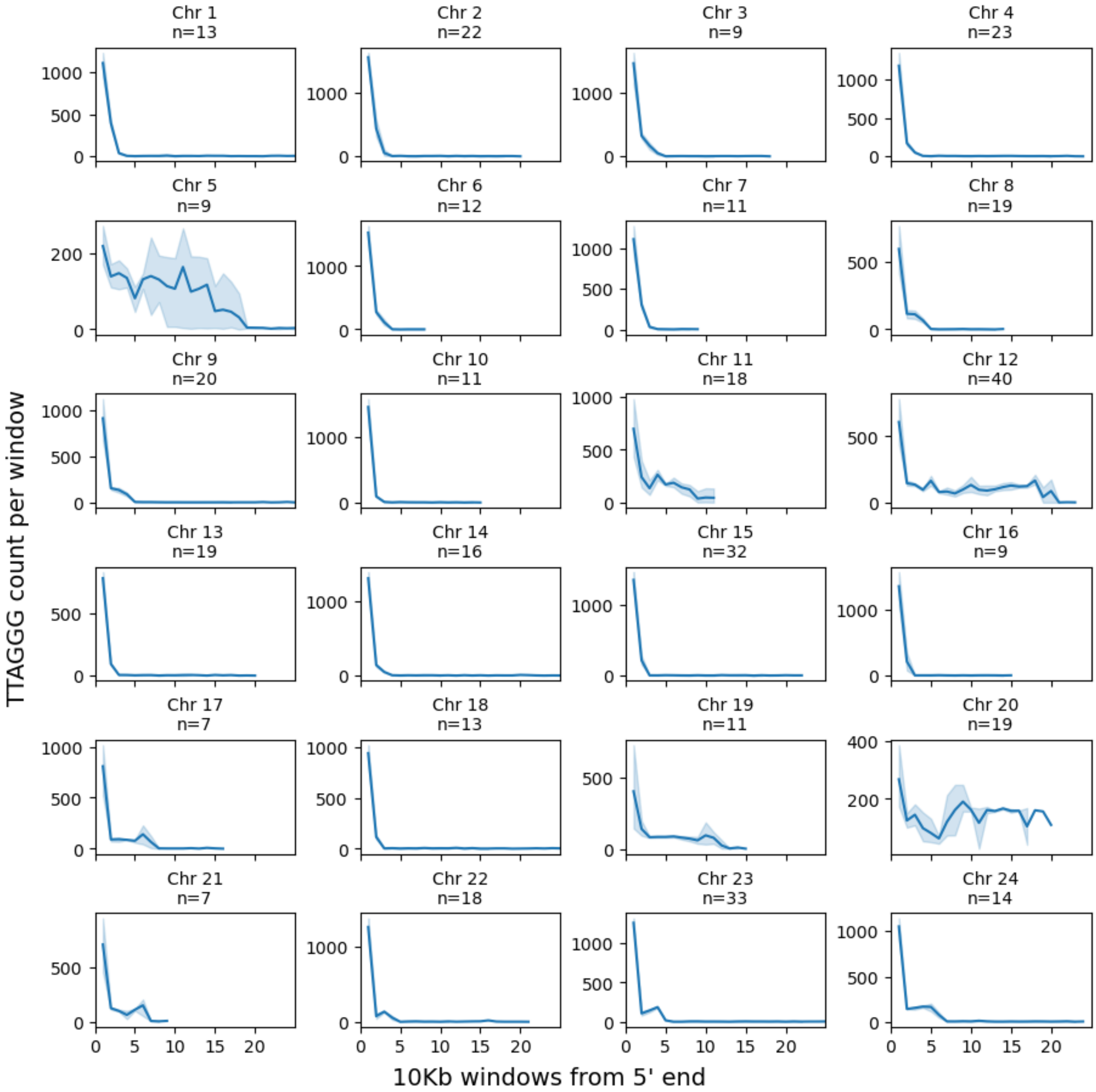

Supplementary figure S21. Distribution of telomeric repeats on Nanopore UL reads covering all 5' chromosome ends.

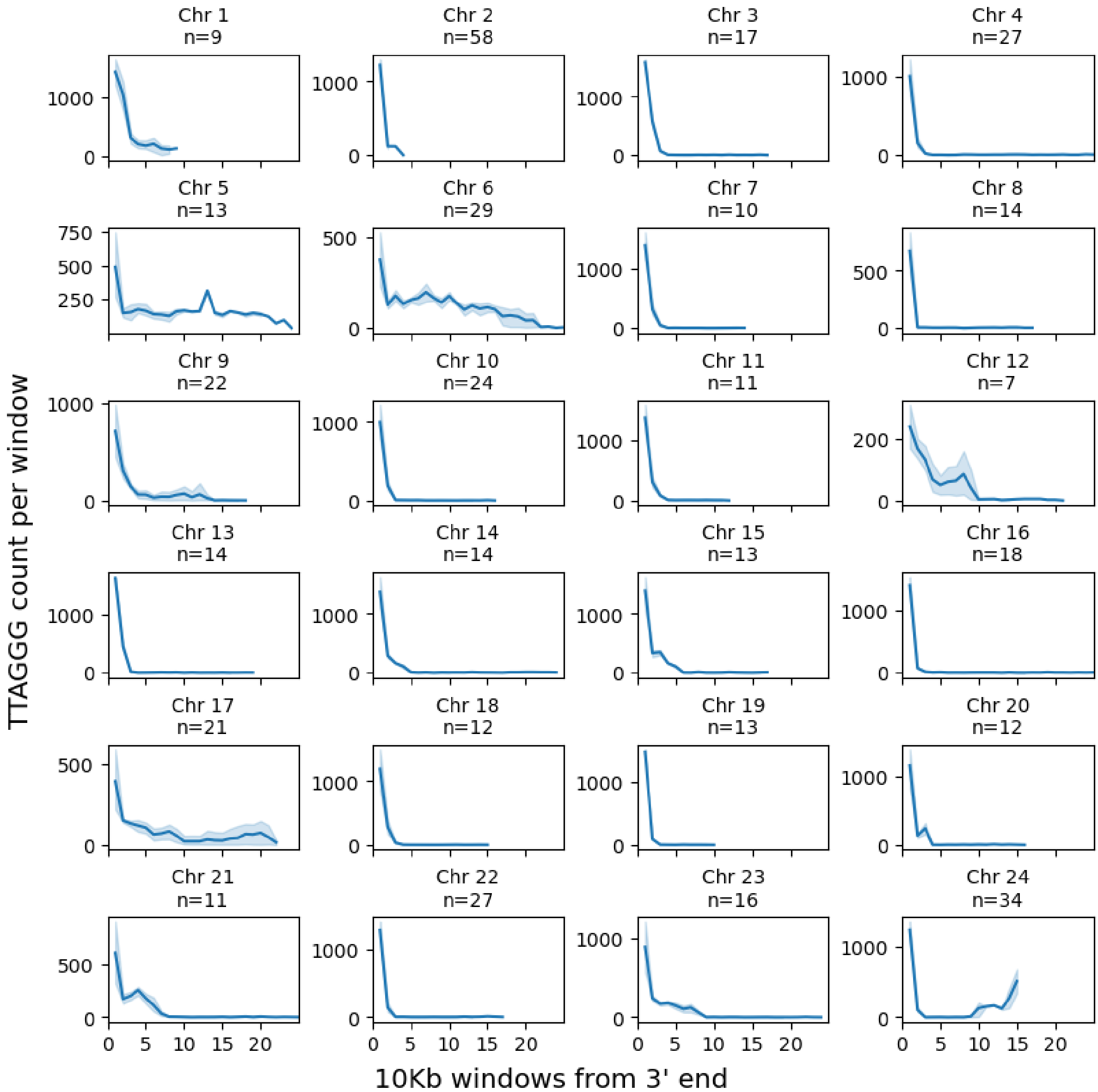

Supplementary figure S22. Distribution of telomeric repeats on Nanopore UL reads covering all 3' chromosome ends.

Supplementary note S2. Gene annotations

Methods and results

The *C. tuca* genome was annotated with different homology-based methods, with TOGA and GALBA tools outperforming Braker. BRAKER2 (Brůna et al., 2021) was run including all described Actinopterygii proteins in Orthodb v.11 (n=3 million) (Kuznetsov et al., 2023)). On the other hand, GALBA (Brůna et al., 2023) was run using Cichliformes proteins in Orthodb v.11. Lastly, TOGA (Kirilenko et al., 2023) was used to infer gene annotations and assign ortology to *Oreochromis niloticus* genes. There were no indicatives of contamination in annotations and these were concordant to other fish gene sets (Supplementary figure S3).

Gene annotation with Braker resulted in 33,966 genes with mono:multi exon ratio of 0.16: GALBA resulted in 26,240 genes and a mono:multi ratio of 0.13; TOGA predictions included 22455 Intact, probably

intact and uncertain lost genes and showed a 0.097 mono:multi ratio. All annotations showed a high BUSCO score and high OMARK completeness (Supplementary figures S2 and S3).

Nevertheless, there is an increase of “unknown” and unexpected duplicated genes (n=5890, n=1971, respectively) in Braker annotations, in comparison with other fish genomes and to GALBA and TOGA annotations (Supplementary figure S3). Manual inspection of these showed that they are rather short (N50/L50 = 153bp/1581), and frequently represented exons of incomplete gene predictions (Supplementary figure S23). On the contrary, TOGA presented a slightly higher number of lost genes (5.45%) and a lower number of duplicates (1.45%) in comparison to GALBA (3.73% missing, 3.10% duplicated; Supplementary figure S3).

As mentioned above, the annotation step was performed in an intermediate assembly version and later transferred to the final assembly. The transfer of gene annotations from the 31-sequence assembly to the final assembly resulted in a decrease of 4 galba, 5 toga and 16 braker genes, from 26243, 22459, 33982, genes respectively. The transfer had no impact on busco and other completeness assessments.

A. citrinellus reannotation and software choice

A certain degree of variation is expected from the comparison of annotations performed with heterogeneous tools (Weisman et al. 2022). To test whether the increase of gene counts was due to the software choice, we performed re-annotation of the *A. citrinellus* genome with Braker, with the repeat-masked genome (custom library), which resulted in an approximate gene count (34,688) and unknown percentages to *C. tuca*’s braker annotations, and contrasting to gene counts reported for other cichlids. These observations highlight the importance of using homogeneous annotations when carrying on comparative analysis (Supplementary figure S3; [Weisman, Murray, and Eddy 2022)].

Since there is no RNAseq available at the moment to discard putative artifacts, and due to the incomplete predictions observed in Braker’s results, we preferred *C. tuca* TOGA’s annotations over the others for all downstream analysis, because it has the advantage of simultaneously predicting genes, assigning orthology and estimating the rate of missing exons and genes. It is important to note that genes and isoforms classified as probably lost or lost were previously removed from TOGA annotations, which could bias completeness estimates for this software.

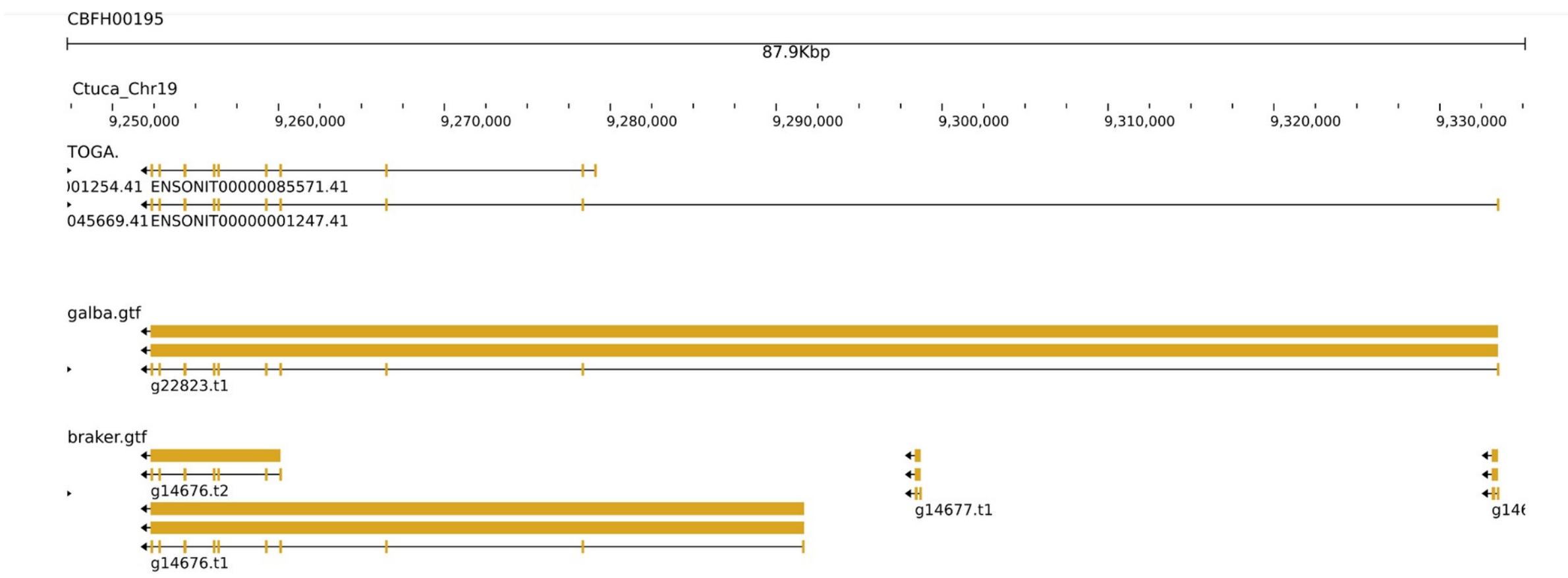

Supplementary figure S23. Comparison of Galba, TOGA and Braker2 annotations.
Braker2 software tends to split genes and produce shorter annotations.

Supplementary note S3. Heterozygosity

Methodology, filtering.

Reads were mapped and variants were called using DeepVariant. Only SNPs with a mapping quality >30 were retained. Minimum and maximum depth thresholds were adjusted based on library-specific mean coverage and dispersion (Supplementary table 4).

The overall heterozygosity rate was similar between HiFi and ONT platforms for *C. tuca* (Supplementary table S8). In both species, chromosomal patterns of SNP heterozygosity were largely comparable to SV heterozygosity, such as elevated heterozygosity on chromosomes 3 and 23, and heterozygosity hotspots for both SV and SNP (Supplementary figures S8 and S9). We found low SNP heterozygosity in the short arms of many acrocentric chromosomes. The analysis of different sequencing platforms yielded consistent results for SNP heterozygosity.

Coalescent rates

Heterozygous SNPs together with mappability masks were used to estimate the coalescent rate for haplotypes of each chromosome of both species (Supplementary figure S5). We observed a lower scaled coalescent rate for *C. missioneira* than *C. tuca*. Assuming panmixia, 3 years generation time (personal observations), and a 3.5e-9 mutation rate (Malinsky et al., 2018), this difference in coalescent rate would mean larger historical population sizes for *C. missioneira* than *C. tuca*. Nevertheless, other processes that we hypothesized relevant for *Crenicichla*, have proven to affect coalescent rates other than population size, such as assortative mating and population structure (Mazet et al., 2016).

Supplementary table S8. Comparison of SNP heterozygosity.

	**Callable sites**	**SNPs only**	**Min. depth**	**Max. depth**
C. tuca HIFI	820370295	744863 (0.09%)	10	50
C. tuca ONT	814902895	701451 (0.09%)	15	40
C. missioneira ONT	736377643	1023817 (0.14%)	7.5	30

Supplementary note S4. Structural comparison of LG3 haplotypes

Haplotypes of LG3 of *C. tuca* and *C. missioneira* were recovered from the hifiasm raw assemblies. First, haplotypic assemblies were mapped to C. tuca final assembly, LG3 mapping contigs were extracted and visualized with Jbrowse2. Synteny maps between haplotypes and between haplotypes and the primary assembly were obtained using Dgenies, and visualized on Jbrowse. *C. tuca* primary assembly (reference) harbors the first haplotype version of LG3, while haplotype 2 was fragmented in 4 contigs. Higher fragmentation was observed on *C. missioneira*, in which H1 and H2 were represented in 20 and 21 contigs respectively. Despite fragmentation, we could indentify conserved synteny within haplotype blocks (contigs). *C. tuca* haplotypes differed by two low complexity blocks in the p-arm (Supplementary Figure S24A, 1 and 2), with many duplicated and inverted alignments between haplotypes. One of these blocks is absent in C. missioneira while the other appears to be present in both its haplotypes in the same orientation than *C. tuca* H2 but apparently differs in copy number, and appears to be duplicated in *C. missioneira* H2. There was a region with few large duplications distally on q-arm of C. tuca H1, lying close to the zoomed region in the main article (Supplementary Figure S24A. 3). The same region was not found on *C. missioneira* but the detection of presence/absense of these regions is hindered by assembly fragmentation.

Finally, many short inverted duplicated alignments were found between p- and q- arm distal regions in all comparisons (darker and blue diagonal lines in Supplementary Figure S24A). Interestingly, the raw assembly graph of LG3 formed a circular structure, reflecting the existence of shared sequences between both chromosomal ends (SUpplementary Figure S24B).

The phylogenetic clustering of LG3 haplotypes in the candidate region for TSP was additionally tested with Maximum Likelihood, considering the General Time Reversible model (GTR) and the best fitting model according to model selection (Tamura 3-parameter) in MEGA. The trees with the highest log are shown on figure S25. Both trees share the same topology as Neighbour Joining, but show slight differences on branch lengths.

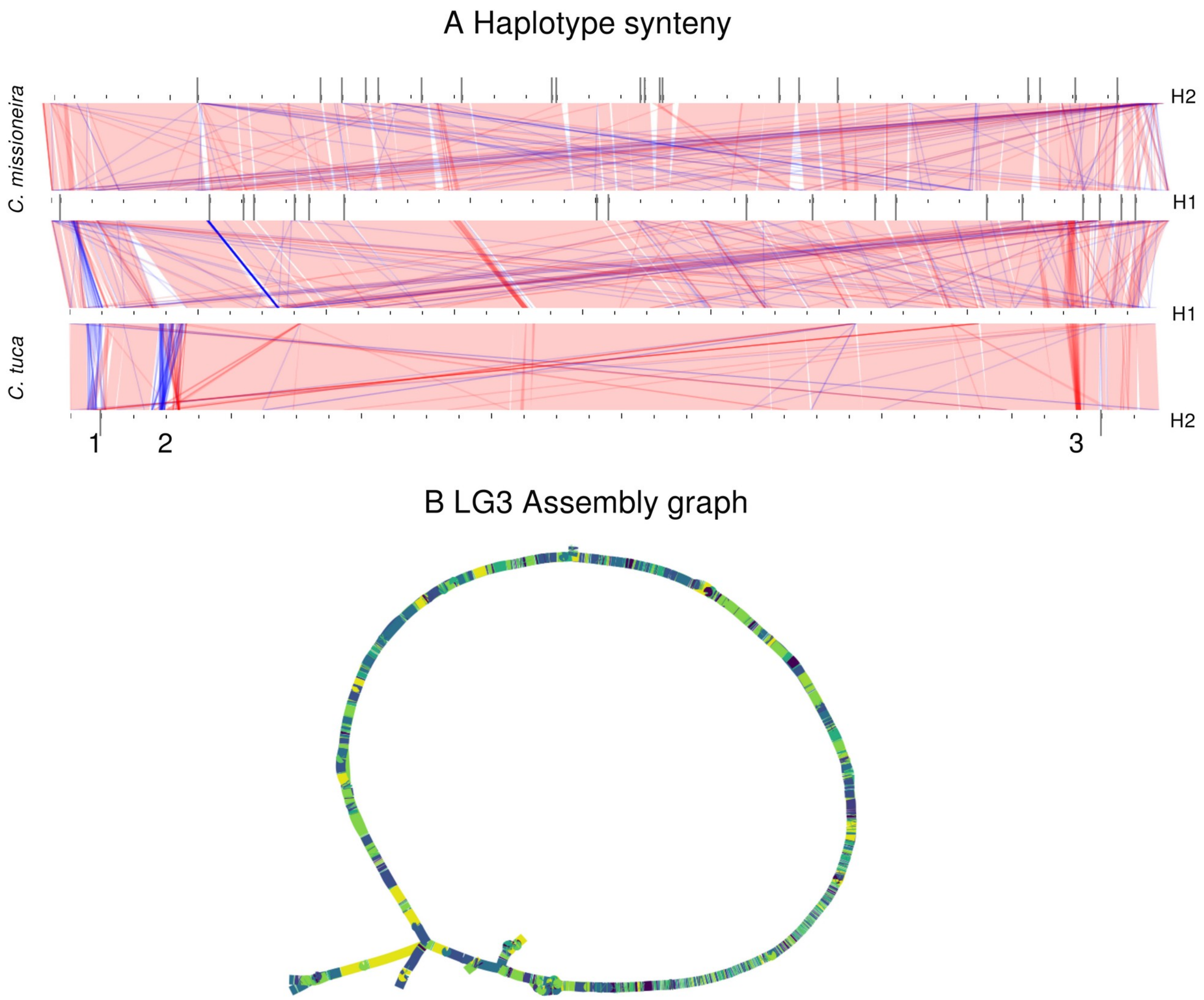

Supplementary Figure S24. Structural complexity of LG3 haplotypes. A. Synteny between C. missioneira and C. tuca haplotypes. B. C. tuca LG3 assembly graph.

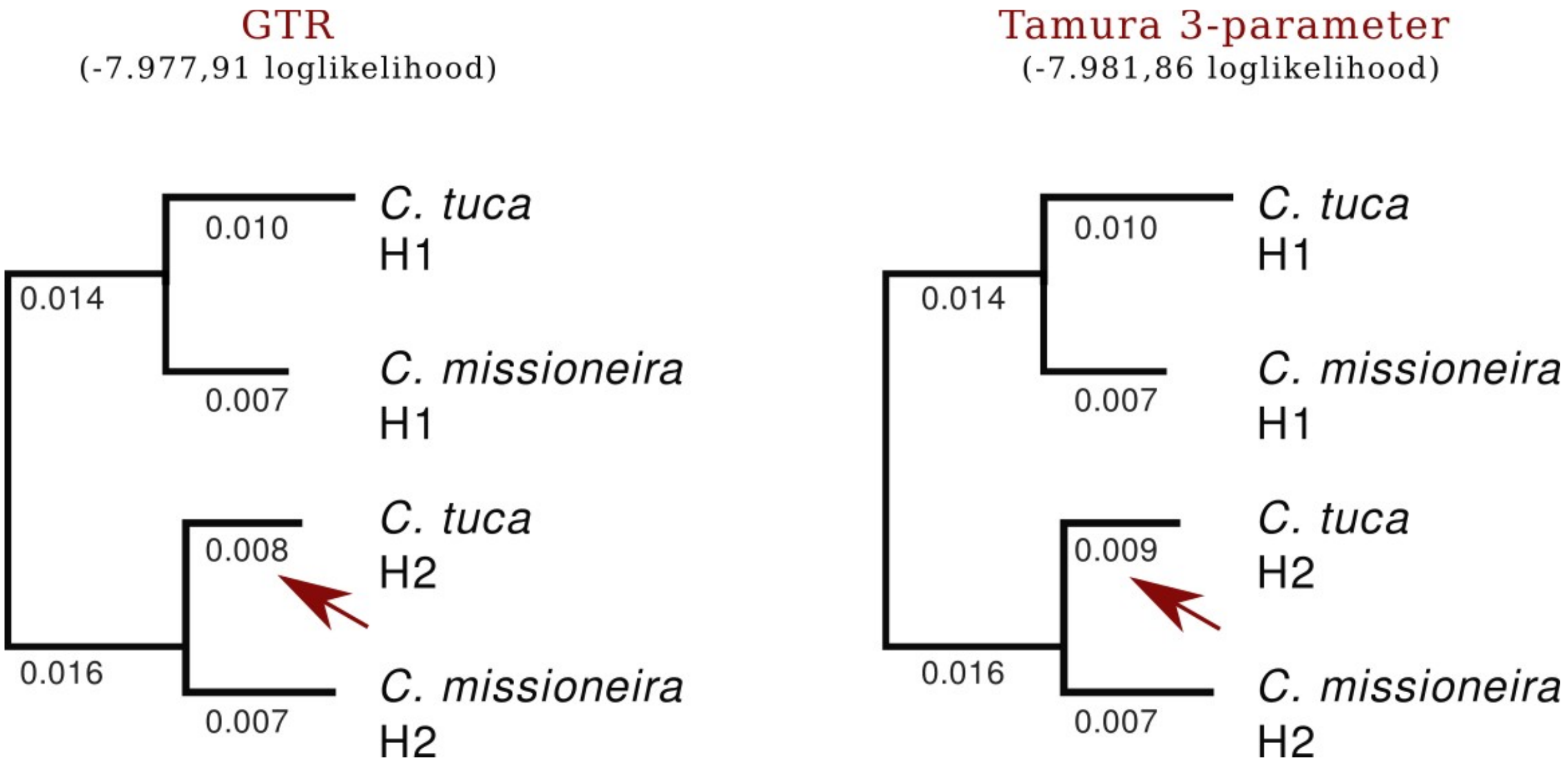

Figure S25. Haplotype tree reconstruction. The trees are drawn with branch lengths (shown next to the branches) computed using the Maximum Likelihood method and measured in the number of substitutions per site. A. using GTR model, B. considering Tamura 3P. The arrows indicate branch length differences.

Supplementary note S5. Manual TE curation

This note outlines the steps taken to manually curate the collection of transposable elements (TEs) obtained with EarlGrey and RepeatModeler2. The process builds upon the suggestions from Goubert et al. (Goubert et al., 2022) and uses some of the scripts published there. Nevertheless, no manual extension was performed, but rather assessed the automatic extension produced by EarlGrey. Custom code and integration can be found on TEcuration.sh script on github.

1. **Pfam Database Search**: The initial step involved scanning the sequences against the Pfam protein family database using the pfam_scan.pl tool. Protein hits were then classified based on their relationship to TEs based on bibliographic and database searches. Custom categories included: TE-associated; Binding-related; TE_domain; Inconclusive; Not_associated. Repeats with hits with repetitive genes and no TE-related features (see below) were removed.
2. **TE-aid Analysis:** TE-aid was employed to assess structural features of consensus sequences and the degree of representation of the sequences (by visualizing depth and depth per divergence interval). Based on this, I classified elements within the "keep" and "remove" categories. Sequences categorized for removal typically had a low number of hits or exhibited a chimeric consensus sequence. Moreover, combining Pfam results and TE-aid structural and functional results, when possible, unknown sequences were classified (Supplementary figure S26).
3. **Replacement of chimeric sequences (Supplementary figure S27):** Sequences classified for removal were further inspected with TE-aid within the RepeatModeler output. In some cases, the original RepeatModeler consensus sequences were reinstated because they were more representative of the genomic copies than the over-extended EarlGrey output.
4. **Clustering redundant sequences:** Cd-hit tool was used to cluster sequences with high similarity within the refined library using the following parameters:

   * Sequence identity threshold: 95% (-aS 0.95 -c 0.95)
   * Global alignment (-G 0)
   * Local alignment (-g 1)

* Minimum word length (-b 500)

5. **Filtering Low-Copy repeats**: Finally, consensus sequences with a reduced number of copies (2 or less hits with minimum similarity of 80% and coverage exceeding 0.5) were removed.

Finally, we used RepeatMasker script RM2Bed.py with overlap resolution “lower_divergence” to generate .bed for repeats. Abundance and divergence from consensus were estimated with RepeatMasker script “calcDivergenceFromAlign” and visualized per chromosome (Supplementary figure S28, S29). Abundance of the different repeats was visualized in a heatmap per chromosome, and those repeats with more than 150000 accumulated bases in at least one chromosome were selected as potential cytogenetic markers for future research (Supplementary figure S).

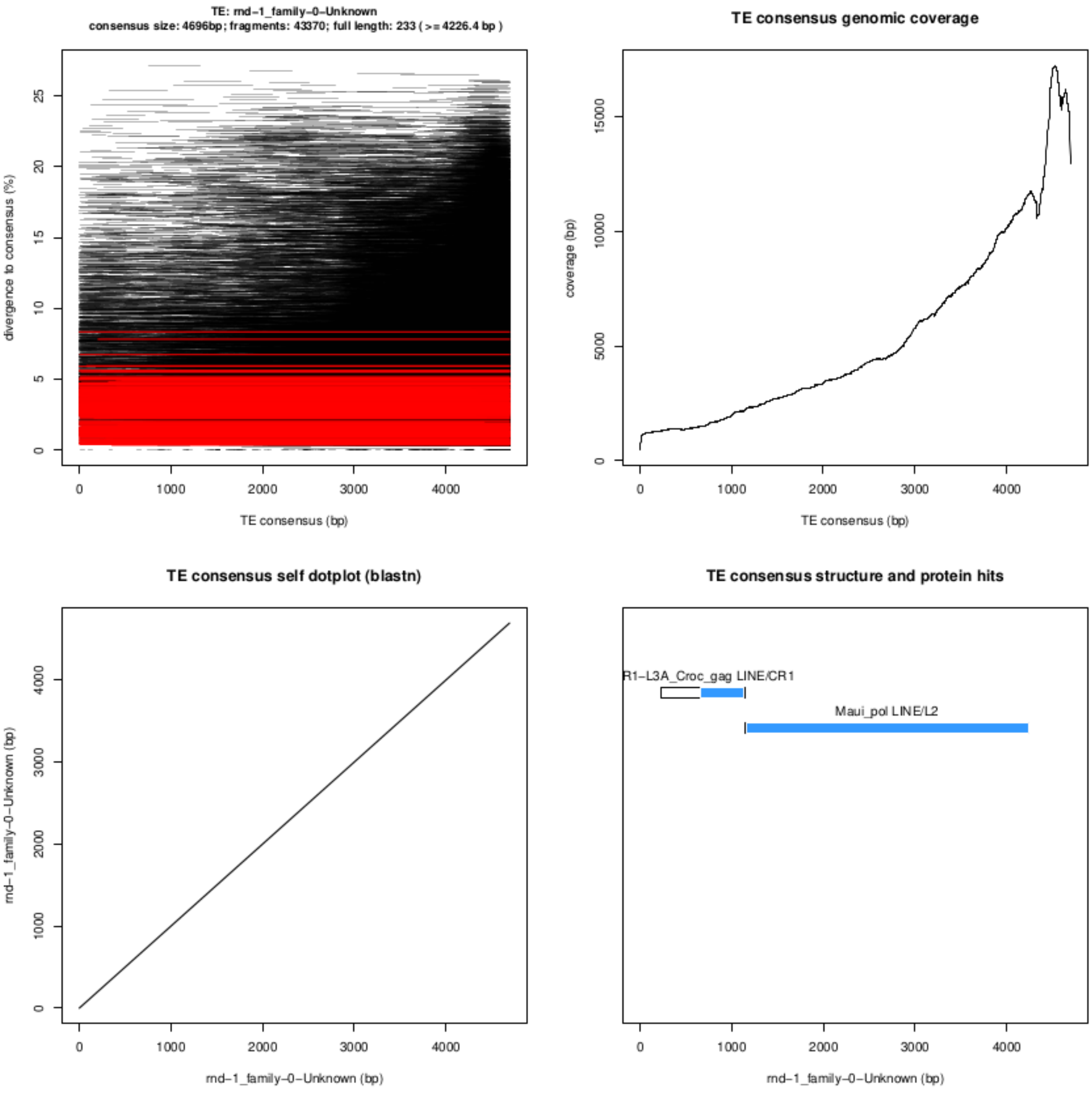

Supplementary figure S26. Examples of a manually classified TE.

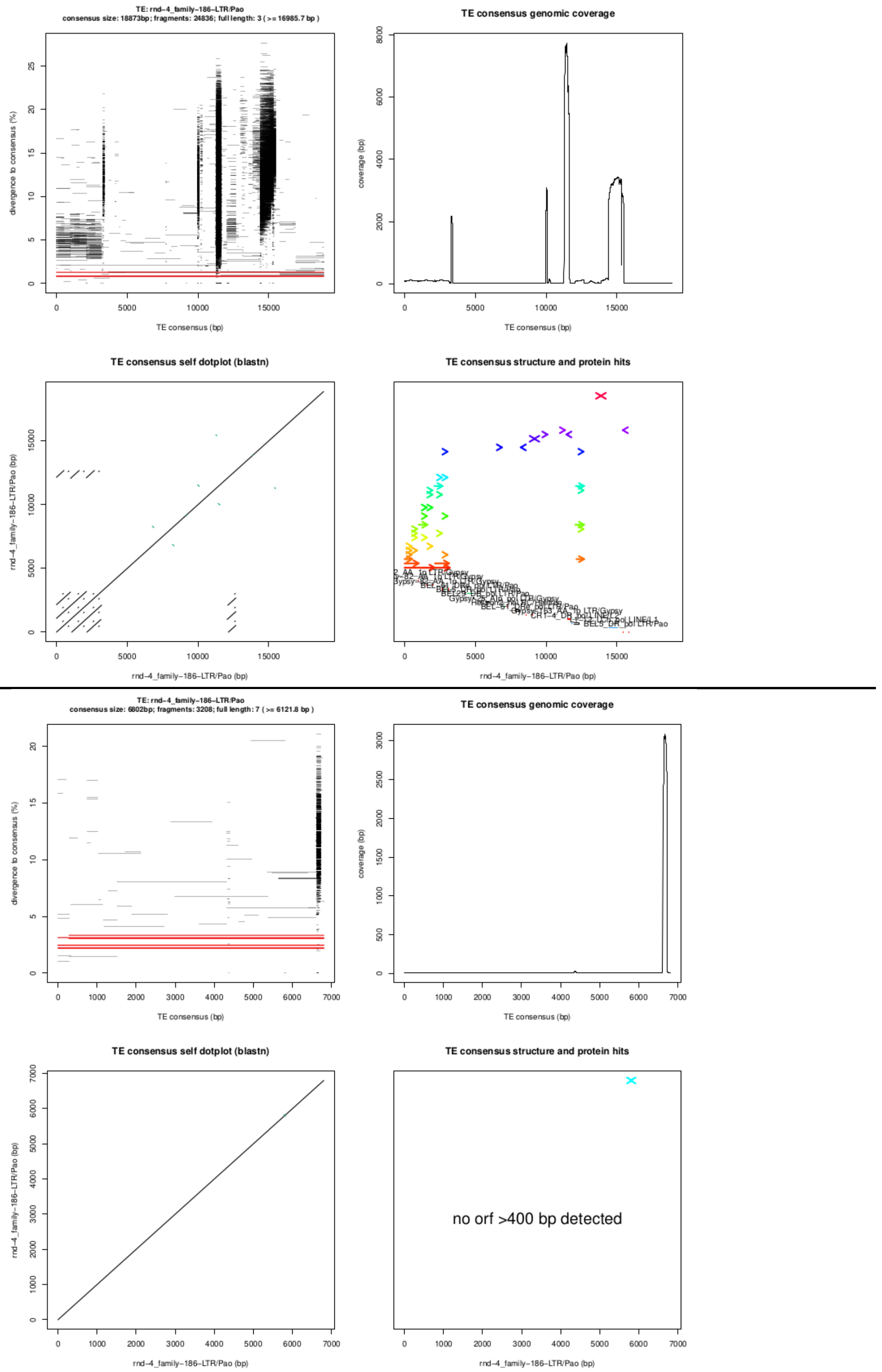

Supplementary figure S27. Example of EarlGrey chimeric over-extended repeats (above) which was manually replaced by the RepeatModeler consensus (below).

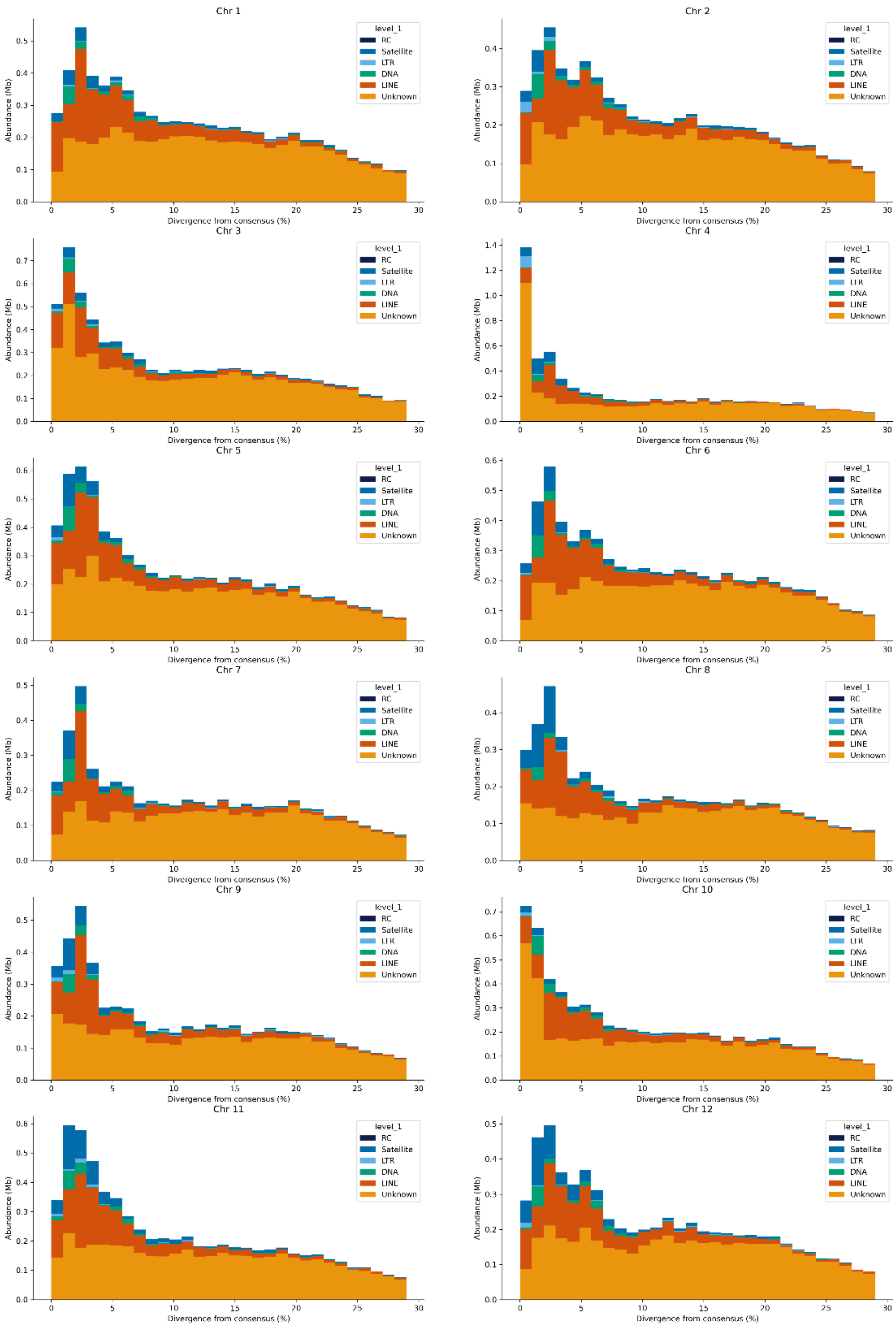

Supplementary figure S28. Repeat Landscape of chromosomes 1-12

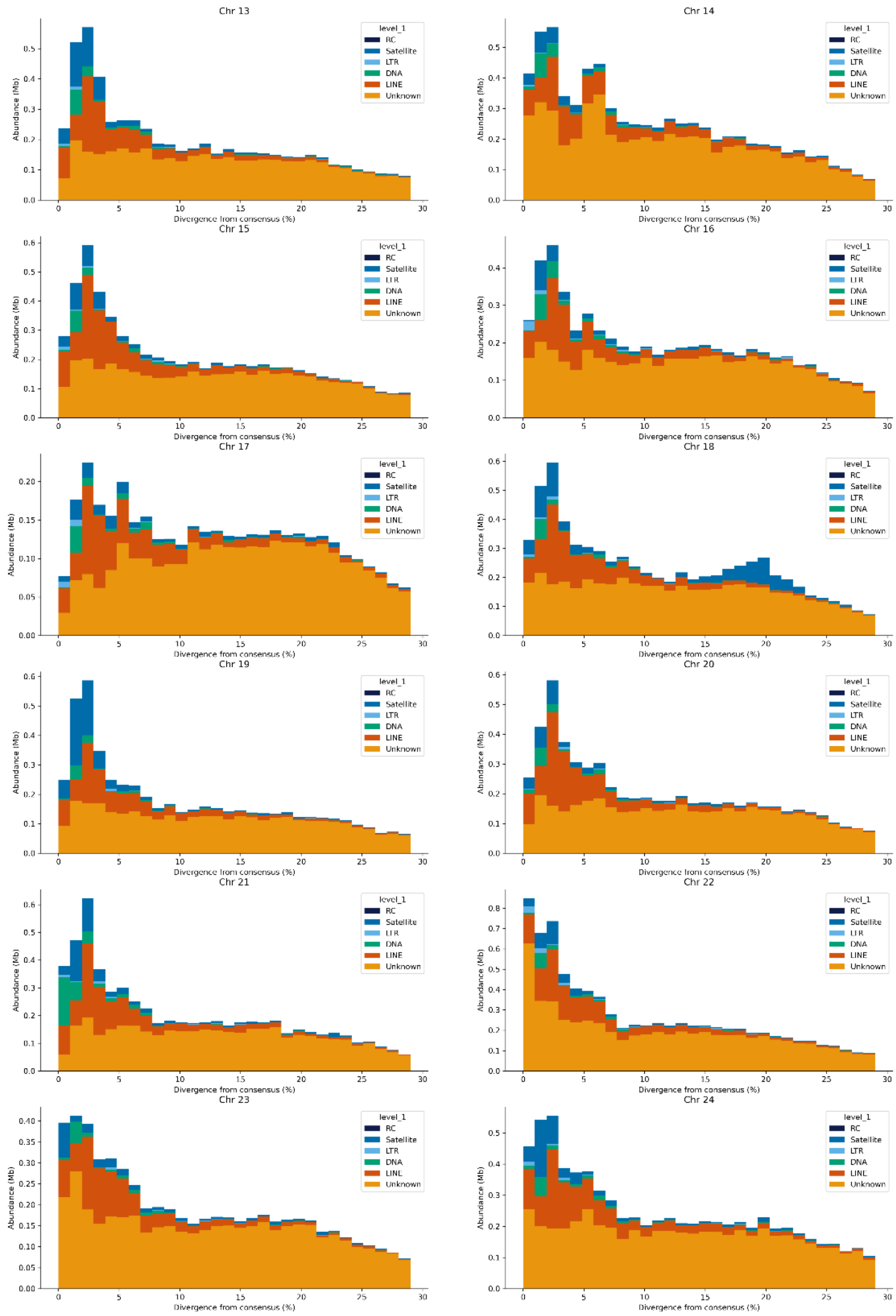

Supplementary figure S29. Repeat Landscape of chromosomes 13-24

Supplementary note S6. *C. missioneira* assembly

We conducted a preliminary assessment of genome assembly performance for *C. missioneira*. ONT reads were trimmed and assembled using hifiasm in ONT mode. Misassemblies were corrected with Tigmint, haplotigs and duplicated repeats were removed with purge_haplotigs, and scaffolding was performed using ntLink. Assembly quality was evaluated using the same methods and Nextflow workflow as for *C. tuca*.

Multiple assemblies were generated, with the final scaffolded version showing the highest contiguity (N50 = 17 Mbp; 162 contigs) and the lowest duplication rate. We assessed the continuity of the genome assembly by whole genome alignment to *C. tuca*'s genome, using minimap2 and visualizating with dgenies. However, all *C. tuca* chromosomes were fragmented into at least two contigs, with some highly fragmented (e.g., Chr20 split into 28 contigs; Supplementary figure S30). The assembly was nearly complete based on read mapping (99.73%) and BUSCO completeness (99% complete genes), but it exhibited lower base and structural accuracy compared to *C. tuca* (QV = 32 vs. 40), with a high split-read mapping rate (6.17%) and at least 425 structural errors. Due to these limitations, and with improvements to the *C. missioneira* assembly expected, we chose to perform structural variant calling by read mapping to the *C. tuca* reference rather than relying on assembly-based comparisons.

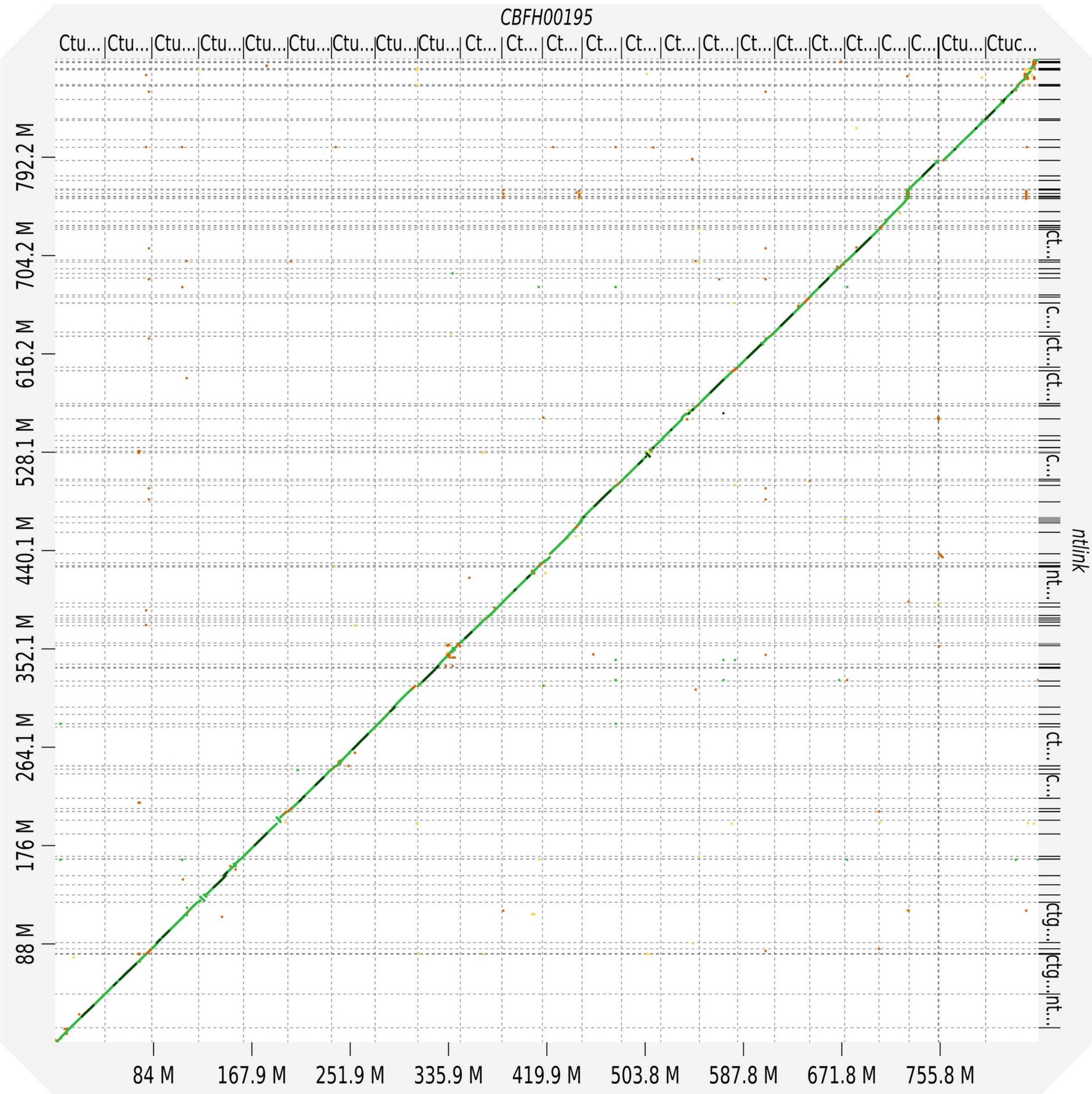

Supplementary figure S30. Whole genome alignment between *C. tuca* and *C. missioneira* assembly.
